# The Potential Impact of Edible Fruit Extracts on Bacterial Nucleases in Preliminary Research—In Silico and In Vitro Insight

**DOI:** 10.3390/ijms26041757

**Published:** 2025-02-19

**Authors:** Łukasz Szeleszczuk, Malwina Brożyna, Bartłomiej Dudek, Marcin Czarnecki, Adam Junka, Monika E. Czerwińska

**Affiliations:** 1 Department of Organic and Physical Chemistry, Medical University of Warsaw, Banacha 1, 02-097 Warsaw, Poland; 2 Platform for Unique Models Application, Department of Pharmaceutical Microbiology and Parasitology, Faculty of Pharmacy, Wroclaw Medical University, Borowska 211, 50-556 Wroclaw, Poland; malwina.brozyna@umw.edu.pl (M.B.); bartlomiej.dudek@umw.edu.pl (B.D.); adam.junka@umw.edu.pl (A.J.); 3 Department of Infectious Diseases, Liver Diseases and Acquired Immune Deficiencies, Wroclaw Medical University, Koszarowa 5, 51-149 Wroclaw, Poland; marcin.czarnecki@umw.edu.pl; 4 Department of Biochemistry and Pharmacogenomics, Medical University of Warsaw, Banacha 1, 02-097 Warsaw, Poland; 5 Centre for Preclinical Research, Medical University of Warsaw, Banacha 1B, 02-097 Warsaw, Poland

**Keywords:** bacterial biofilm, Cornelian cherry, Japanese quince, gut microbiota, sea buckthorn

## Abstract

The extracts from fruits of *Chaenomeles japonica* (Thunb.) Lindl. ex Spach (CJE), *Cornus mas* L. (CME), and *Hippophaё rhamnoides* L. (HRE) are known inhibitors of a variety of eukaryotic hydrolases, engaged in the digestion of fats and polysaccharides. However, there are no data on their potential interaction with the bacterial hydrolases participating in the replication of microbial nucleic acids. This analysis predicted the interaction of the most abundant constituents of HRE, CJE, and CME with the bacterial nucleases. The analysis covered the molecular docking of isorhamnetin glycosides, procyanidins C1 and B2, epicatechin, loganic acid, and cornuside with bacterial enzymes (*Escherichia coli* endonuclease 1, colicin E9, and ribonuclease H; or *Staphylococcus aureus* thermonuclease and nuclease SbcCD). The suggested complexes have been subjected to molecular mechanics with generalized Born and surface area solvation (MM/GBSA) calculations. The second aim was the in vitro evaluation of the influence of the CJE, HRE, and CME on the metabolic activity of bacterial biofilm of selected microbial strains, as well as fibroblasts (L929) and adenocarcinoma intestinal cells (Caco-2) toxicity. Among all extracts, CME showed the most relevant effect on the survival of planktonic cells and biofilm of *E. coli* and *Pseudomonas aeruginosa*. As a result of in silico studies, most virtual hits were predicted to inhibit the proteins under investigation, except for procyanidin C1. Further research on the direct interaction of phytochemicals and selected enzymes in vitro is required and challenged.

## 1. Introduction

The global rise in antibiotic resistance marks the dawn of a post-antibiotic era, underscoring the urgent need for novel antimicrobials that can effectively combat pathogenic bacteria while sparing microbial species that are beneficial to human homeostasis. Traditional antibiotics, although potent against pathogens, often indiscriminately eradicate beneficial microbes that constitute humans’ regular microbiota. It is particularly well-documented regarding bacteria of the large intestine. The eradication during antibiotic therapy can lead to dysbiosis, a state linked to various health complications, such as increased susceptibility to infections, inflammatory diseases, and impaired immune function [1]. On the one hand, the gut microbiota (GM) participates in developing digestive system diseases. On the other hand, any unfavorable changes in the body result in changes in the microbiota composition and properties. Thus, the development of selective antimicrobial agents that preserve the balance of GM is of paramount importance.

The human gastrointestinal (GI) tract hosts a complex community of microorganisms, collectively known as the gut microbiota. It is integral to various physiological processes, including digestion, nutrient absorption, and immune modulation. Beneficial strains like *Bifidobacterium* and *Lactobacillus* play a crucial role in maintaining gut homeostasis, whereas pathogens such as *Escherichia coli* and *Staphylococcus aureus* can form resilient biofilms, contributing to chronic infections and resistance to conventional antibiotics [1,2]. The challenge lies in selective targeting these pathogenic biofilms without harming the homeostatic/probiotic bacteria that are essential for human health. Microbes in the GI tract prevent pathogenic colonization by competing for attachment sites or nutrient sources [1]. The concept that the nutrient landscape dictates which organisms can successfully colonize and persist in the gut was first proposed in Rolf Freter’s nutrient niche theory [3].

In this context, natural compounds derived from edible fruits offer a promising avenue for selective antimicrobial therapy. Fruits such as *Chaenomeles japonica* (Thunb.) Lindl. ex Spach (Japanese quince; CJ), *Cornus mas* L. (Cornelian cherry; CM), and *Hippophaë rhamnoides* L. (sea buckthorn; HR) are rich in bioactive compounds, including flavonoids, proanthocyanidins, and iridoids [4,5]. These compounds have demonstrated inhibitory effects on various eukaryotic hydrolases, such as *α*-amylase and lipase [4,5]. However, their potential interactions with bacterial nucleases, key enzymes in bacterial DNA replication and repair, remain underexplored.

Nucleases play a critical role in the stability and resilience of bacterial biofilms [6]. These enzymes facilitate the degradation of extracellular DNA, which is considered one of the major components of the biofilm matrix, thereby enabling the biofilm’s formation and maintenance. Inhibition of these nucleases could disrupt biofilm integrity, rendering the bacteria more susceptible to antimicrobial agents [7]. Hence, targeting bacterial nucleases represents a strategic approach to combat biofilm-associated infections while preserving beneficial microbiota. So far, only a few reports highlighted the potential inhibition of human nucleases such as 5′-nucleotidase and human flap endonuclease-1 by phenolic compounds [8,9], as well as some interactions between flavonoids and nucleic acids have been considered [10,11,12]. The effects of green tea polyphenols such as gallic acid, (-)-epicatechin gallate, and (-)-epigallocatechin gallate on angiogenin (ANG) 4-induced angiogenesis and RNase A were established. In addition to inhibiting the ribonucleolytic activity of ANG, green tea polyphenols exhibited anti-angiogenic properties [13,14,15]. The effect of an increasing number of phenolic hydroxyl groups on enhancing the inhibition process compared to the parent compounds catechin and epicatechin was proven to be substantial for inhibitory activity [16]. According to our knowledge, there is a study showing that flavonoids, such as (-)-epigallocatechin, myricetin, and quercetin, inhibited *E. coli* DNA polymerase I (EC 2.7.7.7) and T7 bacteriophage RNA polymerase (EC 2.7.7.6), but these enzymes belong to the class of transferases. In both DNA and RNA synthesis, (-)-epigallocatechin and quercetin inhibited enzyme reactions by non-competitive and mixed-type inhibition, respectively, concerning template DNAs. Myricetin inhibited DNA and RNA polymerase reactions by mixed- and competitive-type inhibition [17].

In this preliminary research, we focused on two points linked with the influence of plant extracts on potential gut microbiota shaping. To begin with, this study aims to investigate the interaction between fruit-derived phytochemicals and bacterial nucleases using in silico molecular docking techniques. We selected nucleases from both *E. coli* (colicin E9, endonuclease 1, and ribonuclease H) and *S. aureus* (thermonuclease and nuclease SbcCD subunit C) for this analysis. The chosen nucleases are involved in critical processes of DNA replication, DNA repair, and biofilm formation, making them ideal targets for our investigation. Through molecular docking studies, we evaluated the binding affinities and interaction profiles of various compounds, including isorhamnetin glycosides, loganic acid, cornuside, proanthocyanidins (C1 and B1), and epicatechin, with the selected nucleases. These compounds, previously identified in the fruits of Japanese quince [4,18], Cornelian cherry [5], and sea buckthorn [4], exhibit diverse chemical structures and biological activities, which could influence their interactions with bacterial enzymes. The potentially bioactive metabolites of these extract constituents after gastrointestinal digestion were followed previously in vitro [4,19]. On the other hand, the data on their antimicrobial properties are limited. For this reason, the in vitro tests of CJE, CME, and HRE were performed on selected, pathogenic (*E. coli*, *S. aureus*, *Pseudomonas aeruginosa*, *Candida albicans*) or probiotic (*Lactobacillus reuteri*) strains.

The anticipated outcomes of this study will lay the groundwork for further in vitro and in vivo investigations into the selective antimicrobial properties of these fruit-derived compounds. By elucidating the potential of these natural products to inhibit bacterial nucleases, this research seeks to contribute to developing new antimicrobial strategies that safeguard the beneficial gut microbiota while effectively targeting pathogenic bacteria in the post-antibiotic era.

## 2. Results and Discussion

**Figure 1:**
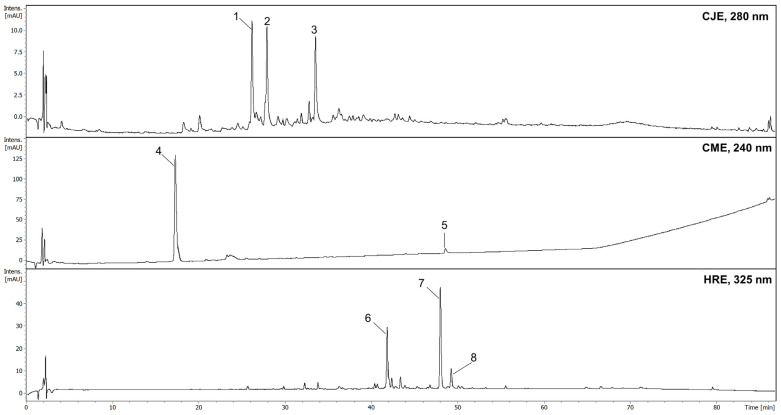
HPLC chromatograms of extracts: CJE—aqueous extract from fruits of *Chaenomeles japonica*; CME—ethanolic (60%) extract from fruits of *Cornus mas*; HRE—aqueous extract from fruit of *Hippophaё rhamnoides*; 1—procyanidin B2; 2—epicatechin; 3—procyanidin trimer; 4—loganic acid; 5—cornuside; 6—isorhamnetin-3-*O*-*β*-D-glucosyl-7-*O*-*α*-L-rhamnoside; 7—isorhamnetin-3-*O*-*α*-L-rhamnosyl-(1→6)-*β*-D-glucoside (isorhamnetin-3-O-rutinoside); 8—isorhamnetin-3-*O*-*β*-D-glucoside.

A metabolite profiling and dereplication of the three fruit extracts were performed by HPLC using an MS and UV detector. We identified compounds from the classes of proanthocyanidins in CJE, iridoids in CME, and flavonoids in HRE (Figure 1). In particular, the most abundant constituents of CME and HRE were previously isolated and described [5,20]. In some regions of Europe, Japanese quince and sea buckthorn fruits are usually used as additives for teas, while tinctures are more often prepared from the fruits of Cornelian cherry. For this reason, we studied the aqueous extracts of Japanese quince and sea buckthorn fruits and the aqueous-ethanolic extract of Cornelian cherry. Based on the spectral data, we established the presence of proanthocyanidin trimer in CJE, which is in agreement with the previous results of Turkiewicz et al. [18]. Considering that procyanidin C1 is a trimer and was detected in the extract of *C. japonica* by Turkiewicz et al. (2022), it was included in further computational studies.

The goal of this study was to assess the potential inhibition of bacterial nucleases by the constituents (mainly phenolic compounds) of the three fruit extracts and to obtain insight into their mode of action using in silico studies. We were further interested in the relationship between phenolic-rich food and the microbiome. Therefore, within the current research, such interactions were first considered using molecular modeling techniques. Considering that novel in silico techniques are excellent approaches to assessing plant-derived products and enzyme interactions, they should be the first step of study design, followed by laboratory experiments. According to our knowledge, molecular modeling is likely to be the first step for screening for inhibitory activity of phenolic compounds on a wide range of bacterial hydrolases, in particular, nucleases representing the classes of endo- and exonucleases participating in bacterial DNA replication or repair, or degradation of RNA (RNases). The algorithms and computational tools allowed us to dock phenolic compounds as potentially useful to the pharmaceutical industry and support their role in traditional medicine, dietary prevention, and gut microbiota shaping.

The phyla *Firmicutes* and *Bacteroidetes*, followed by *Actinobacteria*, *Proteobacteria*, and *Verrucomicrobia*, are predominantly present in healthy mammalian GM. These phyla remain constant, although extensive diversity, both qualitative and quantitative, of the genera is observed in subsequent parts of the intestine [1,3]. In the physiological state, these commensal bacteria or mutualists participate in the fermentation of carbohydrates and positively influence lipid metabolism by providing short-chain fatty acids, inter alia, butyrate [1]. Alterations of the microbiota composition and changes in its optimal functions caused by antibiotics, dietary ingredients, and lifestyle factors are associated with the expansion of pathogenic populations, resulting in various metabolic and immune diseases. It is already known that diet is a particularly important factor in determining the microbiota composition and affecting the development of a properly functioning microbiota. The mode of gut microbiota modification has not been known. For this reason, we decided to find out if the plant-derived compounds might interact with bacterial nucleases. We believe that these enzymes participating in the hydrolysis of nucleic acids can be affected by phytochemicals. Firstly, we decided to perform in silico experiments. According to our knowledge, this is the first study of the interaction between polyphenols and bacterial nucleases.

**Figure 2:**
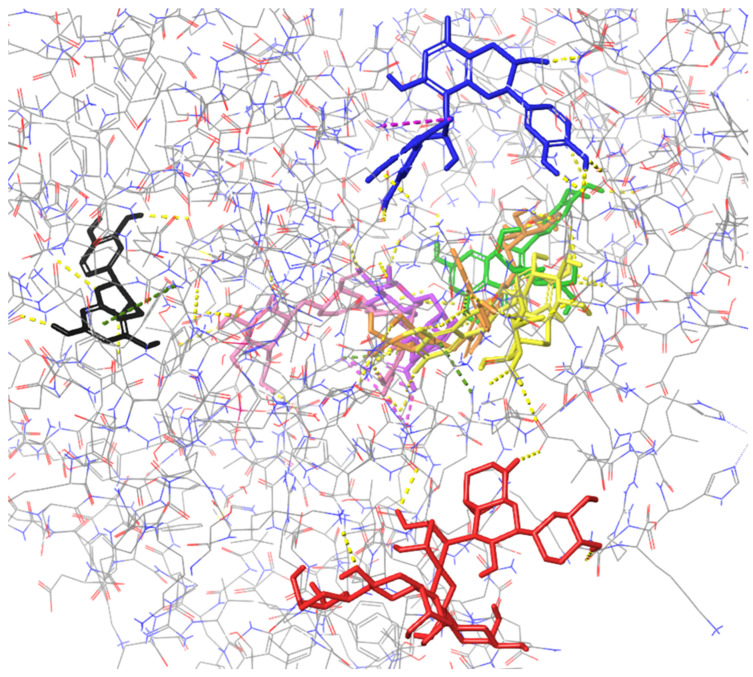
Binding site visualization of the colicin E9. The colors of the compounds are as follows: procyanidin B2 (blue), cornuside (pink), isorhamnetin-3-*O*-*β*-D-glucosyl-7-*O*-*α*-L-rhamnoside (orange), isorhamnetin-3-*O*-rutinoside (yellow), loganic acid (violet), isorhamnetin 3-*O*-*β*-D-glucoside (green), procyanidin C1 (red), epicatechin (black). The coloring of the compounds is consistent for all the studied proteins and corresponds with the font color used for the names of the compounds in the tables.

**Figure 3:**
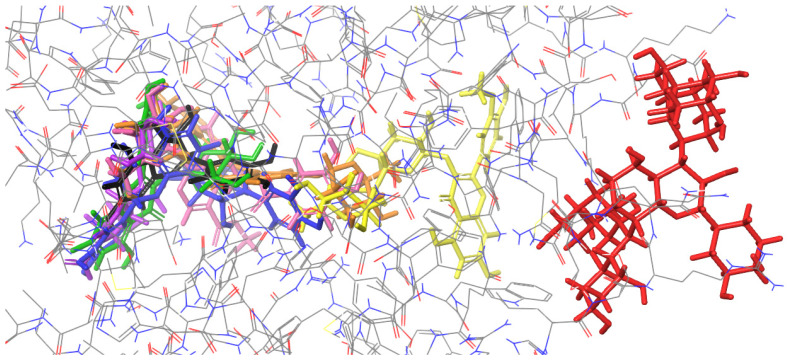
Binding site visualization of the endonuclease 1. The colors of the compounds are as follows: procyanidin B2 (blue), cornuside (pink), isorhamnetin-3-*O*-*β*-D-glucosyl-7-*O*-*α*-L-rhamnoside (orange), isorhamnetin-3-*O*-rutinoside (yellow), loganic acid (violet), isorhamnetin 3-*O*-*β*-D-glucoside (green), procyanidin C1 (red), epicatechin (black). The coloring of the compounds is consistent for all the studied proteins and corresponds with the font color used for the names of the compounds in the tables.

**Figure 4:**
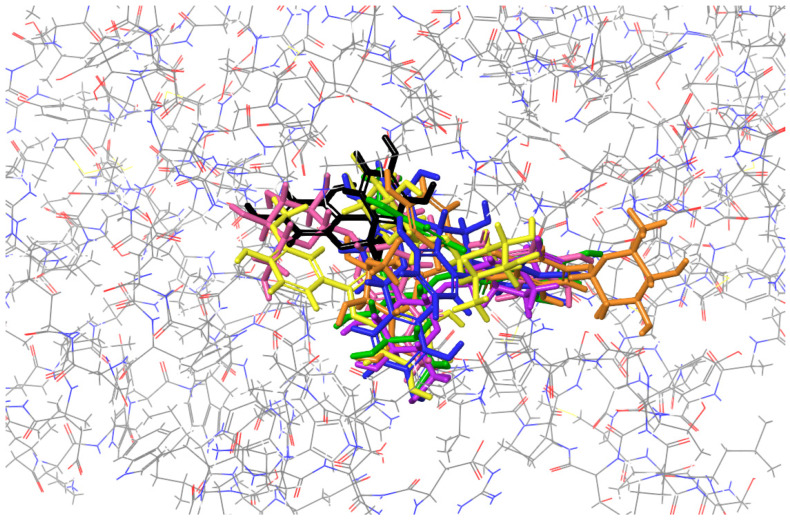
Binding site visualization of the ribonuclease H. The colors of the compounds are as follows: procyanidin B2 (blue), cornuside (pink), isorhamnetin-3-*O*-*β*-D-glucosyl-7-*O*-*α*-L-rhamnoside (orange), isorhamnetin-3-*O*-rutinoside (yellow), loganic acid (violet), isorhamnetin 3-*O*-*β*-D-glucoside (green), epicatechin (black). The coloring of the compounds is consistent for all the studied proteins and corresponds with the font color used for the names of the compounds in the tables.

**Figure 5:**
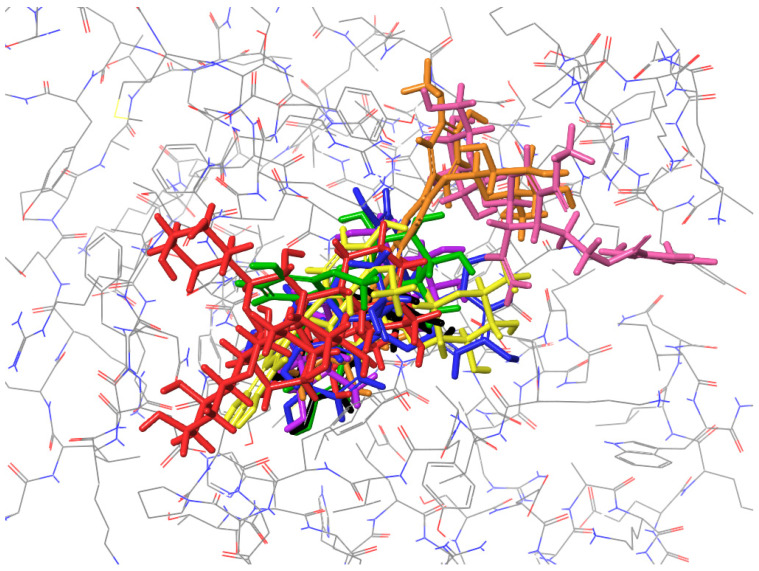
Binding site visualization of the thermonuclease. The colors of the compounds are as follows: procyanidin B2 (blue), cornuside (pink), isorhamnetin-3-*O*-*β*-D-glucosyl-7-*O*-*α*-L-rhamnoside (orange), isorhamnetin-3-*O*-rutinoside (yellow), loganic acid (violet), isorhamnetin 3-*O*-*β*-D-glucoside (green), procyanidin C1 (red), epicatechin (black). The coloring of the compounds is consistent for all the studied proteins and corresponds with the font color used for the names of the compounds in the tables.

The results of molecular docking will be divided into paragraphs, each one describing the results for a particular protein, and presented as follows:

(1) Tables with the results of the molecular docking and MM/GBSA calculations, sorted from the best docking score obtained (Tables S1–S5).

(2) Figures presenting the location of each of the compounds (Figure 2, Figure 3, Figure 4 and Figure 5). To improve the clarity, the H atoms were omitted. Additionally, the coloring of the compounds is consistent for all the studied proteins and corresponds with the font color used for the names of the compounds in the tables.

(3) Tables with the compound–protein interactions formed (Table 1, Table 2, Table 3, Table 4, Table 5 and Table 6).

(4) Discussion of the results.

The 2D compound–protein interaction diagrams are presented in Supplementary Materials (Figures S1–S39).

### 2.1. Molecular Docking to Colicin E9

All the studied compounds have been successfully docked to colicin E9, showing similar affinity, except for procyanidin B2, which was characterized by the best docking score and MM/GBSA values, indicating the strongest interactions with the macromolecule (Figure 2). Contrary to other proteins presented below, for colicin E9, the compounds tend to bind in diverse sites, involving various residues. For cornuside, isorhamnetin-3-*O*-*β*-D-glucosyl-7-*O*-*α*-L-rhamnoside, isorhamnetin-3-*O*-rutinoside, loganic acid, and isorhamnetin 3-*O*-*β*-D-glucoside, a common binding site was observed, and residues such as ASP B: 104, C: 224 were involved in forming the intermolecular interactions (Table 1). Procyanidin C1 and epicatechin bound outside this cavity. Therefore, they were characterized by the worst docking scores. Procyanidin B2 was characterized by the highest affinity and a unique binding site.

**Table 1:** Predicted compound–protein interactions of identified constituents and colicin E9.

	H-Bond	Cation-π	Salt Bridge	Figure
Procyanidin B2 ^1^	ASP D:504, ASN E:670, PHE E:686, LYS E:689, TYR E;699		LYS D:416	Figure S6
Cornuside	ASP B:104, GLU B:112, ASP B:117, LYS C:297, ASN C:272, SER C:274	LYS C:289	LYS B:16, LYS C:289	Figure S8
Isorhamnetin-3-*O*-*β*-D-glucosyl-7-*O*-*α*-L-rhamnoside	ASP B:104, HIE B: 102, ILE B:130, ARG B:132, ASP C:224			Figure S3
Isorhamnetin-3-*O*-rutinoside	SER B:30, ARG B: 132, LYS C:221, ASP C:224, GLY E:627, GLN E:691, LYS E:725, ASP E:729	ARG B:126		Figure S4
Loganic acid	ASP B:104, ASN C:272, THR C:287, GLN C:292,		LYS C:289	Figure S7
Isorhamnetin 3-*O*-*β*-D-glucoside	ASP C:220, LYS C:221, LEU C:223, ASP C:224, PHE C:286			Figure S5
Procyanidin C1	LYS D:404, SER D:508, ASP E:729, GLY E:733			Figure S2
Epicatechin	MET B:1, SER B:3, SER B:108, GLY D:495,	ARG D:496		Figure S9

^1^ The coloring of the compounds is consistent for all the studied proteins and corresponds with the font color used for the names of the compounds in the tables.

### 2.2. Molecular Docking to Endonuclease 1

Most of the docked compounds, namely, cornuside, loganic acid, isorhamnetin-3-*O*-*β*-D-glucosyl-7-*O*-*α*-L-rhamnoside, isorhamnetin 3-*O*-*β*-D-glucoside, procyanidin B2, and epicatechin, bound at the same active site (Figure 3), forming interactions with, among others, LYS 57, VAL 80, TRP 82, and ASN 130 (Table 2). However, the best docking score was obtained for isorhamnetin-3-*O*-rutinoside, which binds outside the aforementioned site. Procyanidin C1 binds at other protein regions, forming only two H-bonds.

**Table 2:** Predicted compound–protein interactions of identified constituents and endonuclease 1.

	H-Bond	Cation-π	Salt Bridge	π–π Stacking	Figure
Isorhamnetin-3-*O*-rutinoside ^1^	ARG 76, TRP 82, GLU 83, HIE 84 ARG 103, ASN 130, GLY 131				Figure S10
Cornuside	LYS 57, GLY 59, VAL 80, TRP 82, ASN 130		ARG 103		Figure S16
Loganic acid	LYS 57, VAL 80, TRP 82		LYS 57		Figure S17
Isorhamnetin-3-*O*-*β*-D-glucosyl-7-*O*-*α*-L-rhamnoside	LYS 57, GLY 59 VAL 80, TRP 82, ASN 130	ARG 79			Figure S11
Procyanidin C1	SER 139, GLN 190				Figure S14
Isorhamnetin 3-*O*-*β*-D-glucoside	GLY 59, VAL 80, GLU 81	LYS 32	LYS 57		Figure S12
Procyanidin B2	SER 29, LYS 32, LYS 57, VAL 80, TRP 82, ARG 103	ARG 79		TRP 82	Figure S13
Epicatechin	LYS 57, VAL 80, GLU 81, TRP 82				Figure S15

^1^ The coloring of the compounds is consistent for all the studied proteins and corresponds with the font color used for the names of the compounds in the tables.

### 2.3. Molecular Docking to Ribonuclease H

Contrary to the previous cases, all the studied compounds bind at the same active site of ribonuclease H (Figure 4). Interestingly, procyanidin B2, which binds exactly in the middle of this active site, was characterized by the best docking score and MM/GBSA value. The list of common residues forming interactions at this active site includes GLN B:102, GLN B:152, VAL B:153, PRO A:97, and GLN A: 152 (Table 3).

**Table 3:** Predicted compound–protein interactions of identified constituents and ribonuclease H.

	H-Bond	Cation-π	Salt Bridge	Figure
Procyanidin B2 ^1^	GLN A: 152, PRO B: 97, ASP B: 102, GLY B: 150, GLN B: 152, VAL B:153		LYS A:96	Figure S21
Cornuside	VAL A: 96, ASN A: 100, GLN A: 152, ASP B: 102, GLY B: 150, GLN B: 152			Figure S24
Isorhamnetin-3-*O*-rutinoside	THR A: 92, GLN A: 152, VAL B: 98, GLN B: 152			Figure S19
Isorhamnetin 3-*O*-*β*-D-glucoside	PRO A: 97, ASP B: 102, VAL B: 153			Figure S18
Isorhamnetin-3-*O*-*β*-D-glucosyl-7-*O*-*α*-L-rhamnoside	PRO A: 97, GLY B: 150			Figure S20
Loganic acid	PRO A: 97, VAL B: 153			Figure S22
Epicatechin	VAL A: 153, GLU A: 154, PRO B: 97	LYS B: 96		Figure S23
Procyanidin C1	The interaction has not been established.			

^1^ The coloring of the compounds is consistent for all the studied proteins and corresponds with the font color used for the names of the compounds in the tables.

### 2.4. Molecular Docking to Thermonuclease

Similarly to ribonuclease H, a common binding site for all compounds, except for isorhamnetin-3-*O*-*β*-D-glucosyl-7-*O*-*α*-L-rhamnoside and cornuside, has been found (Figure 5). A list of common residues forming the compound-protein interactions includes ASP 40, ASP 83, ASN 118, and TYR 115, with the last one forming either H-bond or π–π stacking interactions (Table 4).

**Table 4:** Predicted compound–protein interactions of identified constituents and thermonuclease.

	H-Bond	Salt Bridge	π–π Stacking	Figure
Isorhamnetin-3-*O*-rutinoside ^1^	ASP 40, ASN 118			Figure S32
Epicatechin	LEU 36, ASP 40, TYR 115, ASN 118			Figure S37
Isorhamnetin 3-*O*-*β*-D-glucoside	LEU 38, ASP 40, ASP 83, TYR 115, ASN 118		TYR 115	Figure S33
Loganic acid	ASP 40, ASP 83, TYR 115, ASN 118			Figure S38
Procyanidin B2	ASP 40, LYS 84, ASN 118	ARG 35, ARG 87	TYR 115	Figure S35
Isorhamnetin-3-*O*-*β*-D-glucosyl-7-*O*-*α*-L-rhamnoside	LEU 36, GLU 43, LYS 84, TYR 85, TYR 113			Figure S34
Procyanidin C1	GLN 80, LYS 84			Figure S36
Cornuside	ASP 21, ARG 35, GLU 43, GLU 52, ARG 87	LYS 49	HIE 46	Figure S39

^1^ The coloring of the compounds is consistent for all the studied proteins and corresponds with the font color used for the names of the compounds in the tables.

### 2.5. Molecular Docking to Nuclease SbcCD Subunit C

**Figure 6:**
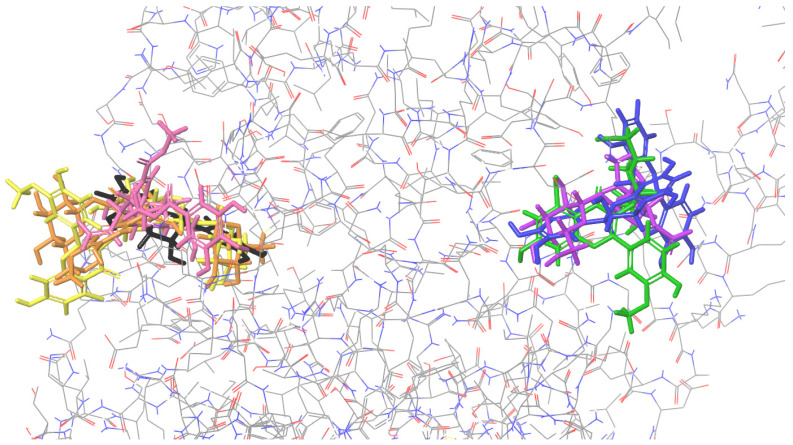
Binding site visualization of the nuclease SbcCD subunit C. The colors of the compounds are as follows: procyanidin B2 (blue), cornuside (pink), isorhamnetin-3-*O*-*β*-D-glucosyl-7-*O*-*α*-L-rhamnoside (orange), isorhamnetin-3-*O*-rutinoside (yellow), loganic acid (violet), isorhamnetin 3-*O*-*β*-D-glucoside (green), epicatechin (black). The coloring of the compounds is consistent for all the studied proteins and corresponds with the font color used for the names of the compounds in the tables.

Docking to nuclease SbcCD subunit C revealed the presence of two sites (Figure 6), at the distinct sites of this macromolecule. Compounds such as isorhamnetin-3-*O*-*β*-D-glucosyl-7-*O*-*α*-L-rhamnoside, epicatechin, isorhamnetin-3-*O*-rutinoside, and cornuside bind to the first one, forming interactions with PRO 152, PHE 156, LYS 157, GLY 915, and ASP 948 (Table 5). The other group, including isorhamnetin 3-*O*-*β*-D-glucoside, procyanidin B2, and loganic acid, binds at the second one, which is stabilized by the H-bonds formed with LEU 174, PHE 175, LYS 179, TYR 860, and GLN 936 (Table 5). No major differences in the docking scores between the compounds docked to the first and second sites have been found. Procyanidin C1 was found not to form a stable interaction with this protein.

**Table 5:** Predicted compound–protein interactions of identified constituents and nuclease SbcCD subunit C.

	H-Bond	Cation-π	Figure
Isorhamnetin-3-*O*-*β*-D-glucosyl-7-*O*-*α*-L-rhamnoside ^1^	PRO 152, PHE 156, ASP 948, GLY 915		Figure S25
Isorhamnetin 3-O-*β*-D-glucoside	PHE 175, LYS 179, TYR 860, GLN 936, SER 937		Figure S27
Epicatechin	PRO 152, GLY 915		Figure S29
Isorhamnetin-3-*O*-rutinoside	PRO 152, GLN 153, PHE 156, LYS 157, GLY 915, ASP 948, GLU 949, GLY 950, GLY 952	LYS 157	Figure S26
Procyanidin B2	LEU 174, ASP 176, LYS 179, TYR 860, GLN 936	LYS 179	Figure S28
Cornuside	GLU 58, GLU 155, LYS 157, GLY 915		Figure S30
Loganic acid	GLY 139, GLN 144, LEU 174, PHE 175, GLN 936		Figure S31
Procyanidin C1	The interaction has not been established.		

^1^ The coloring of the compounds is consistent for all the studied proteins and corresponds with the font color used for the names of the compounds in the tables.

### 2.6. Summary of the Docking Results

Most of the evaluated compounds form interactions with the studied proteins, except for procyanidin C1, which was found not to bind with ribonuclease H nor nuclease SbcCD subunit C. For ribonuclease H and thermonuclease, all compounds were docked to a single, well-defined binding site, while for the other proteins, the compounds occupied different locations. The docking score values obtained for the same protein varied significantly, depending on the compound (Table 6). For example, in the case of ribonuclease H, the docking scores for procyanidin B2 and epicatechin were found to be −14.42 and −6.43, respectively. On the other hand, epicatechin was characterized by a higher affinity than procyanidin B2. This suggests that as the studied compounds differ substantially in their affinities to the chosen proteins, further research on the direct interaction of phytochemicals and selected enzymes in vitro is necessary to confirm the preliminary in silico study. Considering the complex composition of plant extracts, the antimicrobial effect through bacterial enzymes may potentially result from the additive action of multiple components.

**Table 6:** List of the studied compounds, from the one with the highest affinity (1) to the particular protein.

	Colicin E9	Endonuclease 1	Ribonuclease H	Thermonuclease	Nuclease SbcCD Subunit C
**1**	Procyanidin B2 ^1^	Isorhamnetin-3-*O*-rutinoside	Procyanidin B2	Isorhamnetin-3-*O*-rutinoside	Isorhamnetin-3-*O*-*β*-D-glucosyl-7-*O*-*α*-L-rhamnoside
**2**	Cornuside	Cornuside	Cornuside	Epicatechin	Isorhamnetin 3-*O*-*β*-D-glucoside
**3**	Isorhamnetin-3-*O*-*β*-D-glucosyl-7-*O*-*α*-L-rhamnoside	Loganic acid	Isorhamnetin-3-*O*-rutinoside	Isorhamnetin 3-*O*-*β*-D-glucoside	Epicatechin
**4**	Isorhamnetin-3-*O*-rutinoside	Isorhamnetin-3-*O*-*β*-D-glucosyl-7-*O*-*α*-L-rhamnoside	Isorhamnetin 3-*O*-*β*-D-glucoside	Loganic acid	Isorhamnetin-3-*O*-rutinoside
**5**	Loganic acid	Procyanidin C1	Isorhamnetin-3-*O*-*β*-D-glucosyl-7-*O*-*α*-L-rhamnoside	Procyanidin B2	Procyanidin B2
**6**	Isorhamnetin 3-*O*-*β*-D-glucoside	Isorhamnetin 3-*O*-*β*-D-glucoside	Loganic acid	Isorhamnetin-3-*O*-*β*-D-glucosyl-7-*O*-*α*-L-rhamnoside	Cornuside
**7**	Procyanidin C1	Procyanidin B2	Epicatechin	Procyanidin C1	Loganic acid
**8**	Epicatechin	Epicatechin	Procyanidin C1	Cornuside	Procyanidin C1

^1^ The coloring of the compounds is consistent for all the studied proteins and corresponds with the font color used for the names of the compounds in the tables.

### 2.7. Effect of Extracts on Fibroblast and Intestinal Cell Lines’ Viability and Toward Pathogenic and Probiotic Microbial Strain

**Figure 7:**
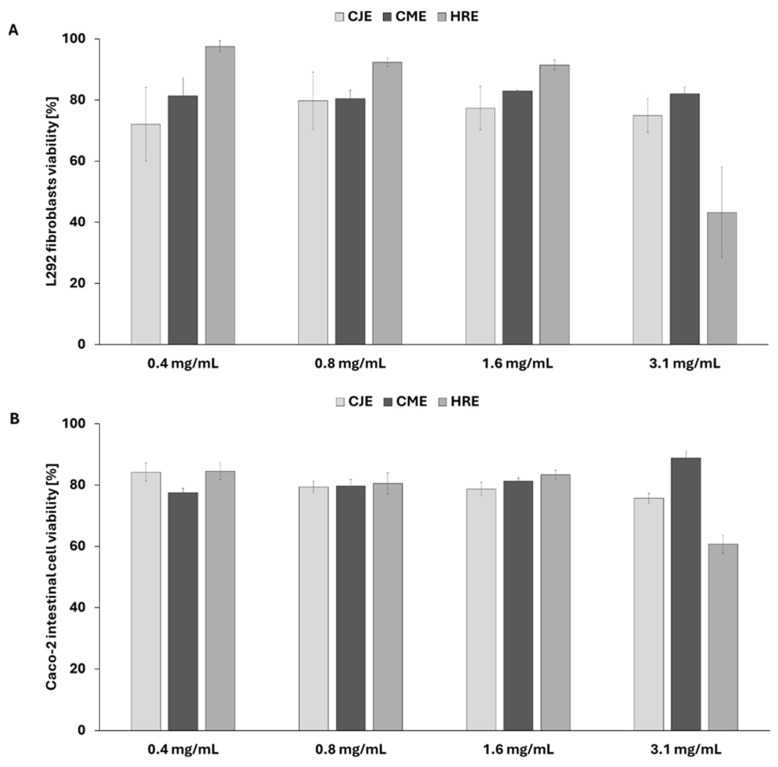
The effect of extracts on fibroblast L929 (**A**) and Caco-2 intestinal cell line (**B**) viability expressed as a percentage of viable control cells. CJE—aqueous extract from fruits of *Chaenomeles japonica*; CME—ethanolic (60%) extract from fruits of *Cornus mas*; HRE—aqueous extract from the fruit of *Hippophaё rhamnoides*.

In this line of investigation, we evaluated the factual in vitro impact of extracts containing compounds tested against Gram-positive *S. aureus* and Gram-negative *E. coli* in silico. For this purpose, *S. aureus* 6538 (methicillin-susceptible *Staphylococcus aureus* MSSA) and *E. coli* 25922 were selected. Additionally, Gram-negative bacteria *Pseudomonas aeruginosa* 27853 and fungus *Candida albicans* 10231, as well as probiotic strain *Lactobacillus reuteri* 55730, were included in the experiment. The method with TTC (tetrazolium chloride solution) used in this study allowed us to evaluate the metabolically active microbial (bacterial and fungal) cells in the biofilm after the treatment with CJE, CME, and HRE. To begin with, we tested the effect of plant extracts on the viability of L929 fibroblast and Caco-2 intestine cell lines (Figure 7). The extracts were considered cytotoxic when the viability of cells dropped below 70%. Only HRE showed a cytotoxic effect in the concentration of 3.1 mg/mL towards both tested cell lines (Figure 7). Therefore, the investigation of the survivability of suspended cells was performed for CJE and CME in the concentration of 3.1 mg/mL, while HRE was tested in the concentration of 1.6 mg/mL.

**Figure 8:**
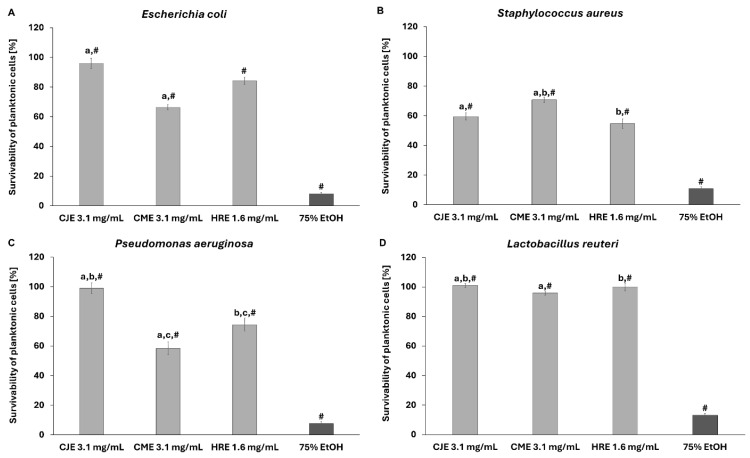
The effect of extracts on suspensions of *E. coli* (**A**), *S. aureus* (**B**), *P. aeruginosa* (**C**), and *L. reuteri* (**D**). The survivability of microbial cells exposed to sterile saline was considered 100%. CJE—aqueous extract from fruits of *Chaenomeles japonica*; CME—ethanolic (60%) extract from fruits of *Cornus mas*; HRE—aqueous extract from the fruit of *Hippophaё rhamnoides*; EtOH—ethanol. The pairs of letters mean the statistical significance (*p* < 0.05) between extracts; # *p* < 0.05 75% EtOH vs. CJE, CME, and HRE.

The applied extracts were not able to completely inhibit the growth of the tested pathogenic strains (*S. aureus*, *E. coli*, *C. albicans*, *P. aeruginosa*) or the probiotic *L. reuteri* strain. Nevertheless, a certain level of growth inhibition was recorded towards pathogenic microbes in the used spectrum of extracts’ concentrations but not against the probiotic strain (Figure 8 and Figure S40).

**Figure 9:**
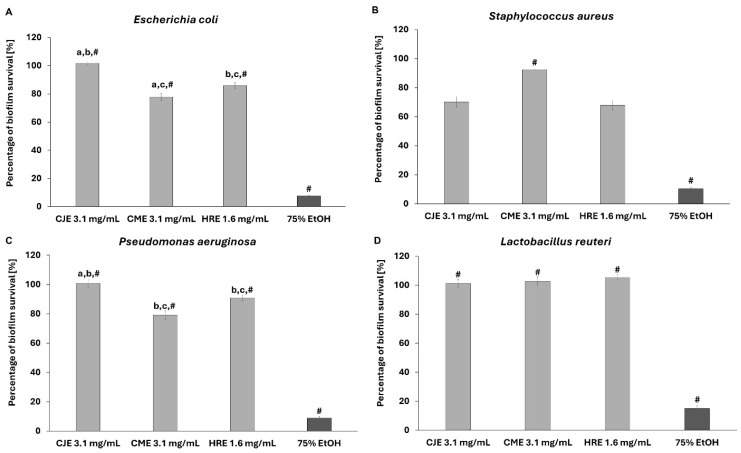
The effect of extracts on the biofilms of *E. coli* (**A**), *S. aureus* (**B**), *P. aeruginosa* (**C**), and *L. reuteri* (**D**). The growth of biofilms exposed to sterile saline was considered 100%. CJE—aqueous extract from fruits of *Chaenomeles japonica*; CME—ethanolic (60%) extract from fruits of *Cornus mas*; HRE—aqueous extract from the fruit of *Hippophaё rhamnoides*; EtOH—ethanol. The pairs of letters mean the statistical significance (*p* < 0.05) between extracts; # *p* < 0.05 75% EtOH vs. CJE, CME, and HRE.

In the next step of the investigation, the impact of the extracts on biofilms formed by pathogenic strains and the probiotic *L. reuteri* strain was investigated (Figure 9 and Figure S40).

Both CJE (3.1 mg/mL) and HRE (1.6 mg/mL) reduced the biofilm absorbance measured after TTC staining by ca. 29.8% and 32%, respectively, in the case of *S. aureus* (Figure 9). On the other hand, CME (3.1 mg/mL) and HRE (1.6 mg/mL) significantly influenced the biofilm of *E. coli* by ca. 22.2% and 14.2%, respectively. Extract of *C. mas* fruit (3.1 mg/mL) affected the biofilm survival of *P. aeruginosa* (Figure 9C) and *C. albicans* (Figure S40) by ca. 20.1% and 23%, respectively. It is worth noting that none of the extracts influenced the survival of the biofilm of *L. reuteri*.

**Figure 10:**
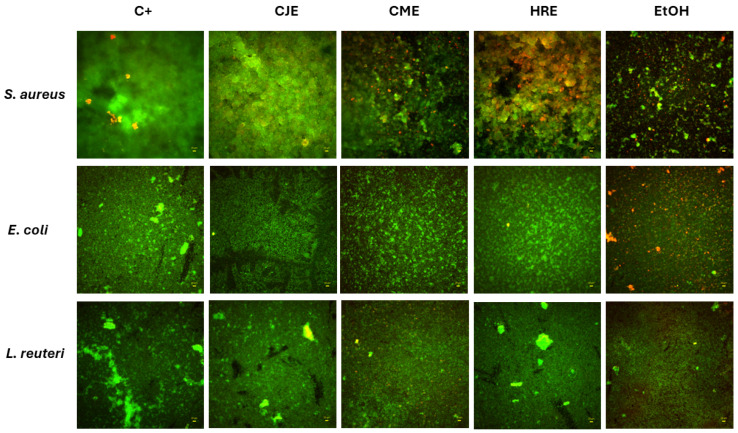
The impact of analyzed extracts and control antimicrobial (ethanol, EtOH) on biofilms of pathogenic *S. aureus*, *E. coli*, and probiotic *L. reuteri*. The green shapes are live, non-damaged biofilm-forming cells; the red shapes are dead/damaged biofilm-forming cells; the black areas are devoid of any types of cells.

To additionally confirm the results presented in Figure 9, the visualization of pathogenic *S. aureus*, *E. coli*, and probiotic *L. reuteri* biofilms exposed to the analyzed extracts was performed through Fluorescence Microscopy (Figure 10).

It is worth noting that microorganisms colonize various ecological niches, in particular, human tissue surfaces, by forming biofilms. In the gastrointestinal tract, single isolated planktonic cells, microorganisms forming biofilms, and biofilm-dispersed bacteria occur. Predominant forms of multicellular communities are supposed to form a biofilm, which is defined as aggregates of microorganisms set in a biopolymer matrix composed of host and microbial compounds, and adherent to food particles, mucus, or epithelia. The biofilm matrix might be of microbial origin or contain non-cellular materials, such as mineral or organic particles like lipopolysaccharide (LPS), which can leak through the injured epithelium [21]. Therefore, the contribution of the host-mucosa biofilm to gut homeostasis in diseases is hypothesized. The positive effect of some nutrients, which have a profound impact on the development of a properly functioning microbiota and interaction with live surfaces, should be considered [1]. On the other hand, lipophilic compounds of plant materials have been considered to disintegrate the outer membrane of bacteria, releasing LPS and increasing the permeability of the cytoplasmic adenosine triphosphate, which indicates the antimicrobial activity of phytochemicals [22]. In some disorders like intestinal bowel disease (IBD), dysbiosis means a reduction of microbial diversity, in addition to an increase in the number of mucosa-associated bacteria, such as an enteropathogenic strain of the B2 phylotype *E. coli* (AIEC). Therefore, adhesion of AIEC and invasion of intestinal epithelium induce the infiltration of immune cells, tumor necrosis factor (TNF)-*α* secretion, and granuloma formation [23]. In recent reports, the impact of Cornelian cherry iridoid-polyphenolic extract and loganic acid on pathogenic *E. coli* strain LF82 growth and adhesion to intestinal epithelial cells in vitro was studied. Moreover, the effect of pretreatment with the extract (20 and 100 mg/kg) and loganic acid (10 and 50 mg/kg) on the intestinal inflammation in 2,4,6-trinitrobenzenesulfonic acid (TNBS)-induced colitis in rats was investigated. The extract of Cornelian cherries inhibited the adherence of both the pathogenic and nonpathogenic *E. coli* strains to intestinal epithelial cells [23].

Polyphenols, as ingredients of vegetables, fruits, and herbal drugs, inhibit non-specifically hydrolytic enzymes included in the digestion process, such as amylases, glucosidases, proteases, and lipases [24]. Most reports concern the interaction between *α*-glucosidase and epicatechin derivatives or proanthocyanidins [25,26,27]. They bind with another site than the active one of this enzyme. Hydrogen bonds or hydrophobic interactions were detected between A- and B-type proanthocyanidin dimers with amino residues of glucosidase [26]. Moreover, derivatives of epicatechin–*α*-amylase interactions were mainly driven by van der Waals and hydrogen bonds, while the proanthocyanidin–*α*-amylase interaction was driven by hydrophobic interaction [28]. Some flavonoid glycosides were studied in the context of interaction with *α*-amylase. The glycosides turned out to be stronger inhibitors than flavonoid aglycons. One of the flavonoids named quercetagetin-7-*O*-*β*-D-glucopyranoside stabilized in the active site of *α*-glucosidase via interactions with the amino acid residues through hydrogen or hydrophobic bonds [29]. Among dietary flavonoids, isorhamnetin-3-*O*-rutinoside, which was included in this study, created hydrogen bonds in active sites of enzymes involved in the insulin signaling pathway. In position 4′ of ring B of flavonoids, conjugative effects were stronger than inductive effects [30]. However, such studies have not been conducted to date as far as nucleases are concerned. There is no doubt that plant-derived products show an effect, but this field was not widely studied.

It is worth noting that secreted RNases are considered hydrolases participating in gut fermentation and degradation of high-molecular-weight RNAs from food [31]. Polyphenols-rich artichoke extract reduced cell proliferation by increasing the DNA damage response mediated by flap endonuclease 1 (FEN1) down-regulation [32]. So far, only a few reports highlighted the potential inhibition of nucleases such as 5′-nucleotidase and hFEN1 by phenolic compounds [8,9]. Based on molecular docking studies, it was suggested that polyphenols such as flavonoids bind to other functional groups than an active site of RNase A [33]. It was found that polyphenols might be involved in hydrogen bond formation, leading to the perturbation of the proper orientation of the members of the catalytic triad (HIS 12, LYS 41, and HIS 119) required for substrate binding. Probably, the activity of RNase is diminished with the interaction of polyphenols with residues such as LYS 37 and ASP 38 or ARG 10-ARG 33, which particularly form hydrogen bonds necessary for proper orientation of *α*-helix and ribonucleolytic activity [33]. Bearing in mind the adverse effects of ANG and the inhibition of angiogenesis by drinking tea, the effects of green tea polyphenols, such as gallic acid, (-)-epicatechin gallate, and (-)-epigallocatechin gallate, on angiogenin (ANG)-induced angiogenesis and RNase A were established [13,14,15]. Moreover, some consecutive basic amino acid residues were established for the binding of ANG4 to bacteria, which is required for the increase in membrane permeability and bacterial cell disruption [34]. However, the research in this field is limited. Some interactions between flavonoids and nucleic acids have been only considered [10,11,12].

The effect of increasing the number of phenolic hydroxyl groups of catechin derivatives on enhancing the inhibition process compared to the parent compounds catechin and epicatechin was proven to be substantial for inhibitory activity [16]. In our study, we did not confirm this statement. We considered the affinity of diverse compounds characterized by different numbers of hydroxyl groups. Taking into account that the extracts of Japanese quince contain both dimers and trimers of proanthocyanidins, such as procyanidin B2 and C1, respectively, we included them in the molecular docking studies to compare their affinity due to increased numbers of hydroxyl groups and the molecular weight of the compound. Even though procyanidin C1 possesses more hydroxyl moieties, it binds at other regions of the endonuclease 1 and colicin E9 than other compounds. Thanks to the high number of hydroxyl groups, it can form only two H-bonds with endonuclease 1 (Figure S14) and thermonuclease (Figure S36). It is known that a simple molecule of catechin is the basic unit of proanthocyanidins, but procyanidin C1 and epicatechin, which is quite like catechin structure, were characterized by the worst docking scores in colicin E9 docking. Procyanidin B2 showed the highest affinity to colicin E9 and a unique binding site. It is worth noting that we did not obtain any interactions of procyanidin C1 with ribonuclease H and nuclease SbcCD subunit C. It is worth noting that among all the tested compounds, cornuside exerted a quite relevant affinity to all enzymes of *E. coli*, such as colicin E9, endonuclease 1, and ribonuclease H. The in vitro tests showed significantly lower survival of planktonic cells and biofilm of *E. coli* after the treatment with CME containing cornuside compared to other extracts. A similar observation could be obtained for other pathogenic strains, such as *P. aeruginosa* and *C. albicans* treated with CME. On the other hand, flavonoids of HRE, contrary to procyanidin B2 from CJE, showed the highest affinity to enzymes of *S. aureus* (Table 6). However, the influence of both HRE and CJE on plankton and biofilm of this bacteria in vitro was comparable. Therefore, it seems that we could not explain this phenomenon by the potential inhibition of thermonuclease and nuclease SbcCD subunit C of *S. aureus*. Searching for further modes of inhibition is necessary, as well as the direct effect of compounds on enzymes in vitro has to be tested.

## 3. Materials and Methods

### 3.1. Structures Preparation

Three-dimensional crystal structures of colicin E9 from *Escherichia coli* (PDB ID: 1FSJ), ribonuclease H from *Escherichia coli* (PDB ID: 4Z0U), and thermonuclease from *Staphylococcus aureus* (PDB ID: 1STN) were retrieved from the RCBS PDB database [35]. Three-dimensional crystal structures of endonuclease 1 from *Escherichia coli* (PDB ID: P25736) and nuclease SbcCD subunit C from *Staphylococcus aureus* (PDB ID: A6QGP8) were retrieved from the AlphaFold database [35,36].

The structures were processed before docking using the Protein Preparation Wizard to remove unwanted water molecules and metal ions. This procedure also simplifies multimeric complexes, creates disulfide bonds, assigns bond orders properly, adjusts ionization states, and fixes the orientation of misoriented groups. Hydrogen atoms were added to the protein structures, and standard protonation states at pH 7.4 were used. The preprocessed structures were then optimized to generate geometrically stable structures under the OPLS 2005 force field. The prepared protein structures were used for further modeling.

### 3.2. Molecular Docking

#### 3.2.1. Active Site Identification and Grid Generation

Due to the absence of cocrystalized molecules, SiteMap suite in Schrödinger software was exploited for accurate identification and evaluation of probable binding sites in the studied proteins. A cubic search box was defined, with the grid size set large enough to fully accommodate all predicted by SiteMap protein–compound binding sites. Receptor grids were generated with the default parameters for the van der Waals scaling factor (1.00) and charge cutoff (0.25) to soften the energy potential for nonpolar parts of the receptor, while other atoms were free of scaling, without any constraints nor excluded volumes, employing the OPLS 2005 force field. Molecular docking calculations were performed using Schrӧdinger Maestro 12.8. version (Schrӧdinger, LLC, New York, NY, USA, 2023).

#### 3.2.2. Compounds Preparation

Structures of compounds used in this work, including isorhamnetin-3-*O*-*β*-D-glucosyl-7-*O*-*α*-L-rhamnoside (ID: 72188972), isorhamnetin-3-*O*-rutinoside (ID: 5481663), isorhamnetin 3-*O*-*β*-D-glucoside (ID: 5318645), procyanidin B2 (ID: 122738), procyanidin C1 (ID: 169853), epicatechin (ID: 72276), loganic acid (ID: 89640), and cornuside (ID: 131348), were retrieved from the PubChem database [37].

The studied compounds were prepared for docking using the LigPrep application from the Schrödinger Suite: protonation states were determined at pH 7.4 ± 2.0, followed by the generation of possible tautomers. For the geometry optimization, the OPLS2005 forcefield was used. Other settings of LigPrep remained at default values

#### 3.2.3. Glide XP–Compound Docking

Flexible protein–compound docking was performed using the Grid-based Ligand Docking with Energetics (Glide) module and extra precision (XP) scheme, with the default parameters for the van der Waals scaling factor (0.80) and charge cutoff (0.15), using the flexible compounds’ sampling. During the docking, the default 20 poses for the initial Glide docking stage were retained, performing the post-docking minimization. The Glide score (GScore) was calculated as GScore = 0.065 × vdW + 0.130 × Coul + Lipo + Hbond + Metal + BuryP + RotB + Site, wherein vdW: van der Waals energy; Coul: Coulomb energy; Lipo: Lipophilic term; Hbond: Hydrogen-bonding; Metal: Metal-binding term; BuryP: Buried Polar groups’ penalty; RotB: Penalty for rotatable bonds that have been frozen; and Site: Active site polar interactions. Emodel combines GlideScore, the nonbonded interaction energy, and for flexible docking, the excess internal energy of the generated compound conformation.

#### 3.2.4. MM-GBSA Calculations

Calculation of binding free energy (ΔG_bind_) values was exploited to estimate in silico compounds’ binding affinities. For accurate calculation of binding free energies, the Molecular Mechanics/Generalized Born Surface Area (MM/GBSA) rescoring method was used [38]. For this purpose, the Prime MM/GBSA module was utilized.

MM/GBSA rescoring was performed for initial compound-docked poses with the best scoring functions. The free energy changes during protein–compound interactions were calculated with the use of the OPLS 2005 force field and the VSGB solvent model.

Binding free energy values were calculated according to the following equation:

MM/GBSA ΔG_bind_ = G_complex(optimized)_ − (G_protein(optimized)_ + G_peptide(optimized)_) (1)

The free energy of each state, i.e., of complex, protein, and peptide, was estimated by accounting for molecular mechanics energies, solvation energies, and entropic terms as follows:

G = G_int_ + G_Coulomb_ +G_vdW_ + G_GB_ + G_lipo_ − TS (2)

where G_int_, G_Coulomb_, and G_vdW_ are standard MM energy terms for bond (covalent, angle and dihedral), Coulomb (electrostatic), and van der Waals interactions; G_GB_ and G_lipo_ are polar and non-polar (lipophilic) contributions to the solvation free energies; while T is an absolute temperature, and S is an entropy value. Polar contribution (G_GB_) was calculated using the generalized Born model, while non-polar contribution (G_lipo_) was estimated based on the solvent-accessible surface area (SASA).

### 3.3. In Vitro Studies of Extracts Rich in Tested Compounds

#### 3.3.1. Plant Materials and Extract Preparation

Fruits of *Chaenomeles japonica* var. *alpina* and *Cornus mas* were collected in October 2018 and September 2017, respectively, in the Botanical Garden of the Center for Biological Diversity Conservation in Powsin (Polish Academy of Sciences, Poland) (52°06′17″ N, 21°05′42″ E). Vouchers of the species (20180821_CJ, 20160914_CM) have been deposited in the Department of Pharmaceutical Biology at the Medical University of Warsaw (Poland). Fruits of *Hippophaë rhamnoides* (Zakład Konfekcjonowania Ziół FLOS, Mokrsko, Poland; batch no. 1058) were purchased in an herbal store. The fruits of CJ and HR were extracted three times with boiling water in a 1:40 (*m*/*v*) ratio for 15 min. A portion of powdered fruits of CM was macerated three times with aqueous ethanol (60%, *v*/*v*) in a 1:10 (*m*/*v*) ratio for 24 h each time. The collected extracts were concentrated under reduced pressure (Rotavapor R-3, Buchi, Switzerland) and lyophilized (Telstar Cryodos 50, Terrassa, Spain).

#### 3.3.2. Chromatographic Conditions

Chromatographic analysis was performed on a UHPLC-3000 RS system (Dionex, Germering, Germany), with DAD and an AmaZon SL ion trap mass spectrometer with an ESI interface (Bruker Daltonik GmbH, Bremen, Germany). Separations were performed on a Kinetex XB-C18 (150 × 2.1 mm, 1.7 μm) column (Phenomenex Inc., Torrance, CA, USA). The column oven temperature was maintained at 25 °C. For preliminary phytochemical analyses of the extracts and fractions, mobile phase A was 0.1% HCOOH in water, and mobile phase B was 0.1% HCOOH in acetonitrile. The gradient program was as follows: 0–60 min. 4–26% B; 60–90 min. 26–95% B (equilibration). The flow rate was 0.2 mL/min. UV spectra were recorded in the 200–800 nm range, and chromatograms were acquired at 240, 280, 325, or 520 nm. The LC eluate was introduced directly into the ESI interface without splitting. The details were previously described [4]. The most abundant constituents of extracts were identified based on comparing mass spectra with literature data and retention times with the isolated compounds [5,20].

#### 3.3.3. Evaluation of Extracts’ Cytotoxicity Towards Fibroblast and Caco-2 Cell Line

The Neutral Red (NR) cytotoxicity assay [39] was performed on L929 fibroblast and Caco-2 intestinal cell lines (ATCC, Manassas, VA, USA) treated with an extract obtained according to the method previously described. Briefly, after 24 h of incubation of cells with extracts, the medium was removed, and 100 μL of the NR solution (40 μg/mL; Sigma-Aldrich, Darmstadt, Germany) was introduced to the wells. Cells were incubated with NR for 2 h at 37 °C. Next, 150 μL of a de-stain solution (50% ethanol 96%, 49% deionized water, 1% glacial acetic acid; POCH, Gliwice, Poland) was added to extract NR from the cells within 30 min. in a microplate shaker (MTS4, IKA-Labortechnik, Staufen, Germany). The absorbance was measured spectrophotometrically in a microplate reader (Multi-scan GO, Thermo Fisher Scientific, Waltham, MA, USA) at 540 nm. The absorbance value of cells not treated with extracts was considered 100% of the potential cellular growth (positive control, control of growth), and the survival percentage [%] was calculated and displayed relative to this baseline. The extracts were tested in the concentration range from 0.4 to 3.1 mg/mL to check their potential cytotoxic effect. According to ISO 10993 [40], part 5, the extracts were considered non-toxic when the viability of cells was above 70%. Therefore, the extracts in the non-toxic concentrations were further investigated.

#### 3.3.4. Evaluation of Extracts’ Activity Towards Pathogens and Probiotic Strain

The research was performed on five reference strains from the American Type Culture Collection (ATCC), namely, *Staphylococcus aureus* 6538 (methicillin-susceptible *Staphylococcus aureus* MSSA), *Escherichia coli* 25922, *Lactobacillus reuteri* 55730, *Pseudomonas aeruginosa* 27853, and *Candida albicans* 10231. All tested strains originated from the Strains Collection of the Department of Pharmaceutical Microbiology and Parasitology, Medical University of Wroclaw, Poland. The standard estimation of the Minimal Inhibitory Concentration in the microtiter plate was performed as described earlier [41]. The microbial solutions non-exposed to extracts but to sterile saline served as a growth control setting; the microbial solutions exposed to 75% ethanol served as a test usability control setting. Because no MIC values understood as 100% of microbial growth inhibition were achieved within tested concentrations, the percentage [%] level of reduction was measured as described earlier [42]. The extracts of CJ and CM were tested in the concentration of 3.1 mg/mL, whereas HRE was tested in the concentration of 1.6 mg/mL due to the toxic effect (towards eukaryotic cells) in the higher concentrations. Each sample was tested in six repeats.

#### 3.3.5. Spectrophotometric Evaluation of Extracts Activity Against Biofilm Formed by Pathogens and Probiotic Strain

The initial stage of the method, involving the biofilm inoculation in the wells of 96-well microtiter plate wells and the introduction of antimicrobials, was performed as previously described [43]. The volume of 100 µL 0.1% tetrazolium chloride solution (2,3,5-triphenyl-2H-tetrazolium chloride, TTC) (PanReac AppliChem, Darmstadt, Germany) was prepared in the Tryptic Soy Broth (TSB) and added to each well (VWR, Radnor, PA, USA) with adhered biofilm and incubated for 2 h at 37 °C. Colorless TTC is transformed into red formazan crystals by metabolically active cells. Next, the red crystals of formazan were dissolved in 100 µL of methanol (Chempur, Piekary Slaskie, Poland). The plate was shaken at 450 rpm for 30 min. (Schuttler MTS-4, IKA, Königswinter, Germany). Next, the solution was transferred into the new plate to measure the absorbance at a 490 nm wavelength (MultiScan Go Spectrophotometer, Thermo Fischer Scientific, Waltham, MA, USA). The extracts of CJ and CM were tested in the concentration of 3.1 mg/mL, whereas HRE was tested in the concentration of 1.6 mg/mL due to the toxic effect (towards eukaryotic cells) in the higher concentrations. The microbial biofilms exposed to 75% ethanol served as a test usability control setting. Each sample was tested in six repeats.

#### 3.3.6. Microscopic Evaluation of Extracts Activity Against Biofilm Formed by Pathogens and Probiotic Strain

The biofilms were cultured and exposed to extracts and 75% ethanol, as described in 2.3.5. Next, the medium was removed from the wells of the microtiter plate, and the biofilms were dyed with the FilmTracer™ LIVE/DEAD™ Biofilm Viability Kit (Thermo Fischer Scientific, Waltham, MA, USA) and visualized, as described before [42], using a fluorescence microscope Lumascope 620 (Etaluma, Carlsbad, CA, USA). A laser line (confocal microscopy) at wavelength 488 nm (500–530 nm emission) was applied to visualize SYTO-9, whereas a wavelength at 552 nm (575–627 nm emission) was applied to visualize propidium iodide (PI) in a sequential mode. The orange/red color showed damaged cells thanks to the PI staining, and the green color of the SYTO-9 staining represented non-damaged cells. The microbial biofilms exposed to 75% ethanol served as a test usability control setting.

## 4. Conclusions

A molecular docking approach allowed us to provide insight into the potential interaction of some secondary metabolites of plant-derived dietary supplements and gut microbiota through bacterial nuclease inhibition. Common binding sites for selected phytochemicals, with some exceptions, were observed for most enzymes, such as endonuclease 1, ribonuclease H, and thermonuclease. Procyanidin C1, despite many hydroxyl groups, probably due to the molecule size, did not allow us to obtain the interactions. On the other hand, procyanidin B2 and isorhamnetin derivatives, as well as cornuside, showed the highest affinity to all tested bacterial enzymes. The extracts, in particular, Cornelian cherry, which are their source, exhibited in vitro inhibition of planktonic cells and biofilm of *Escherichia coli*, while extracts of Japanese quince and sea buckthorn more efficiently influenced *Staphylococcus aureus*. The putative interaction of secondary metabolites of these extracts with bacterial nucleases might partly explain the mode of action of a phenolic-rich diet. However, further investigations with the isolated metabolites and bacterial enzymes will be needed to confirm the predicted bioactivities.

## Data Availability

Data is contained within the article or Supplementary Material.

## Supplementary Materials

The following supporting information can be downloaded at: https://www.mdpi.com/article/10.3390/ijms26041757/s1.

## References

[1] Dudek-Wicher R.K., Junka A., Bartoszewicz M. (2018). The influence of antibiotics and dietary components on gut microbiota. Gastroenterol. Rev..

[2] Aboobacker P.A., Ragunathan L., Sanjeevi T., Manoharan A., Sasi A.C., Chandran V., Kannaiyan K., Samuel M.S. (2023). Synthetic biology’s latest trends in antimicrobial resistance and biofilm. J. Pure Appl. Microbiol..

[3] Pereira F.C., Berry D. (2017). Microbial nutrient niches in the gut. Environ. Microbiol..

[4] Siegień J., Buchholz T., Popowski D., Granica S., Osińska E., Melzig M.F., Czerwińska M.E. (2021). Pancreatic lipase and *α*-amylase inhibitory activity of extracts from selected plant materials after gastrointestinal digestion in vitro. Food Chem..

[5] Świerczewska A., Buchholz T., Melzig M.F., Czerwińska M.E. (2019). In vitro α-amylase and pancreatic lipase inhibitory activity of *Cornus mas* L. and *Cornus alba* L. fruit extracts. J. Food Drug Anal..

[6] Zhang L., Reha-Krantz L.J., Maloy S., Hughes K. (2013). Nuclease. Brenner’s Encyclopedia of Genetics.

[7] Hwang I.Y., Tan M.H., Koh E., Ho C.L., Poh C.L., Chang M.W. (2014). Reprogramming Microbes to Be Pathogen-Seeking Killers. ACS Synth. Biol..

[8] Kavutcu M., Melzig M.F. (1999). In vitro effects of selected flavonoids on the 5′-nucleotidase activity. Pharmazie.

[9] Ma L., Cao X., Wang H., Lu K., Wang Y., Tu C., Dai Y., Meng Y., Li Y., Yu P. (2019). Discovery of myricetin as a potent inhibitor of human flap endonuclease 1, which potentially can be used as sensitizing agent against HT-29 human colon cancer cells. J. Agric. Food Chem..

[10] Kanakis C.D., Nafisi S., Rajabi M., Shadaloi A., Tarantilis P.A., Polissiou M.G., Bariyanga J., Tajmir-Riahi H.A. (2009). Structural analysis of DNA and RNA interactions with antioxidant flavonoids. Spectroscopy.

[11] Srivastava S., Somasagara R.R., Hegde M., Nishana M., Tadi S.K., Srivastava M., Choudhary B., Raghavan S.C. (2016). Quercetin, a natural flavonoid interacts with DNA, arrests cell cycle and causes tumor regression by activating mitochondrial pathway of apoptosis. Sci. Rep..

[12] Costa G., Rocca R., Moraca F., Talarico C., Romeo I., Ortuso F., Alcaro S., Artese A. (2016). A comparative docking strategy to identify polyphenolic derivatives as promising antineoplastic binders of G-quadruplex DNA c-myc and bcl-2 sequences. Mol. Inf..

[13] Panda A., Karhadkar S., Acharya B., Banerjee A., De S., Dasgupta S. (2021). Enhancement of angiogenin inhibition by polyphenol-capped gold nanoparticles. Biopolymers.

[14] Ghosh K.S., Debnath J., Pathak T., Dasgupta S. (2008). Using proton nuclear magnetic resonance to study the mode of ribonuclease A inhibition by competitive and noncompetitive inhibitors. Bioorg. Med. Chem. Lett..

[15] Ghosh K.S., Maiti T.K., Debnath J., Dasgupta S. (2007). Inhibition of Ribonuclease A by polyphenols present in green tea. Proteins.

[16] Dutta S., Basak A., Dasgupta S. (2010). Synthesis and ribonuclease A inhibition activity of resorcinol and phloroglucinol derivatives of catechin and epicatechin: Importance of hydroxyl groups. Bioorg. Med. Chem..

[17] Shinozuka K., Kikuchi Y., Nishino C., Mori A., Tawata S. (1988). Inhibitory effect of flavonoids on DNA-dependent DNA and RNA polymerases. Experientia.

[18] Turkiewicz I.P., Wojdyło A., Tkacz K., Nowicka P. (2022). UPLC/ESI-Q-TOF-MS analysis of (poly)phenols, tocols and amino acids in *Chaenomeles* leaves versus in vitro anti-enzyme activities. Ind. Crops Prod..

[19] Olędzka A., Cichocka K., Woliński K., Melzig M.F., Czerwińska M.E. (2022). Potentially bio-accessible metabolites from an extract of *Cornus mas* fruit after gastrointestinal digestion in vitro and gut microbiota ex vivo treatment. Nutrients.

[20] Laskowska A.K., Wilczak A., Skowrońska W., Michel P., Melzig M.F., Czerwińska M.E. (2022). Fruits of *Hippophaë rhamnoides* in human leukocytes and Caco-2 cell monolayer models—A question about their preventive role in lipopolysaccharide leakage and cytokine secretion in endotoxemia. Front. Pharmacol..

[21] Motta J.-P., Wallace J.L., Buret A.G., Deraison C., Vergnolle N. (2021). Gastrointestinal biofilms in health and disease. Nat. Rev. Gastroenterol. Hepatol..

[22] Nohynek L.J., Alakomi H.L., Kähkönen M.P., Heinonen M., Helander I.M., Oksman-Caldentey K.M., Puupponen-Pimiä R.H. (2006). Berry phenolics: Antimicrobial properties and mechanisms of action against severe human pathogens. Nutr. Cancer.

[23] Szandruk-Bender M., Rutkowska M., Merwid-Ląd A., Wiatrak B., Szeląg A., Dzimira S., Sobieszczańska B., Krzystek-Korpacka M., Kucharska A.Z., Matuszewska A. (2020). Cornelian cherry iridoid-polyphenolic extract improves mucosal epithelial barrier integrity in rat experimental colitis and exerts antimicrobial and antiadhesive activities in vitro. Oxid. Med. Cell Longev..

[24] Hanhineva K., Törrönen R., Bondia-Pons I., Pekkinen J., Kolehmainen M., Mykkänen H., Poutanen K. (2010). Impact of dietary polyphenols on carbohydrate metabolism. Int. J. Mol. Sci..

[25] Wang Y., Xu X., Chen J., Ye X., Pan H., Chen S. (2022). Improving regulation of polymeric proanthocyanidins and tea polyphenols against postprandial hyperglycemia via acid-catalyzed transformation. J. Agric. Food Chem..

[26] Wei M., Chai W.M., Yang Q., Wang R., Peng Y. (2017). Novel insights into the inhibitory effect and mechanism of proanthocyanidins from *Pyracantha fortuneana* fruit on *α*-glucosidase. J. Food Sci..

[27] Han L., Zhang L., Ma W., Li D., Shi R., Wang M. (2018). Proanthocyanidin B(2) attenuates postprandial blood glucose and its inhibitory effect on alpha-glucosidase: Analysis by kinetics, fluorescence spectroscopy, atomic force microscopy and molecular docking. Food Funct..

[28] Jiang C., Chen Y., Ye X., Wang L., Shao J., Jing H., Jiang C., Wang H., Ma C. (2021). Three flavanols delay starch digestion by inhibiting *α*-amylase and binding with starch. Int. J. Biol. Macromol..

[29] Tian J.-L., Si X., Wang Y.-H., Gong E.-S., Xie X., Zhang Y., Li B., Shu C. (2021). Bioactive flavonoids from *Rubus corchorifolius* inhibit *α*-glucosidase and *α*-amylase to improve postprandial hyperglycemia. Food Chem..

[30] Jia Y., Ma Y., Cheng G., Zhang Y., Cai S. (2019). Comparative study of dietary flavonoids with different structures as *α*-glucosidase inhibitors and insulin sensitizers. J. Agric. Food Chem..

[31] Mironova N.L., Zenkova M.A. (2018). Vertebrate RNase 1 homologs: Potential regulators of extracellular RNAs. Biotarget.

[32] Mileo A.M., Di Venere D., Mardente S., Miccadei S. (2020). Artichoke polyphenols sensitize human breast cancer cells to chemotherapeutic drugs via a ROS-mediated downregulation of flap endonuclease 1. Oxid. Med. Cell Longev..

[33] Tripathy D.R., Panda A., Dinda A.K., Dasgupta S. (2021). Positional preferences in flavonoids for inhibition of ribonuclease A: Where “—OH” where?. Proteins.

[34] Sultana M.F., Suzuki M., Yamasaki F., Kubota W., Takahashi K., Abo H., Kawashima H. (2022). Identification of crucial amino acid eesidues for antimicrobial activity of angiogenin 4 and its modulation of gut microbiota in mice. Front. Microbiol..

[35] Berman H.M., Westbrook J., Feng Z., Gilliland G., Bhat T.N., Weissig H., Shindyalov I.N., Bourne P.E. (2000). The Protein Data Bank. Nucleic Acids Res..

[36] Varadi M., Anyango S., Deshpande M., Nair S., Natassia C., Yordanova G., Yuan D., Stroe O., Wood G., Laydon A. (2021). AlphaFold Protein Structure Database: Massively expanding the structural coverage of protein-sequence space with high-accuracy models. Nucleic Acids Res..

[37] Kim S., Thiessen P.A., Bolton E.E., Chen J., Fu G., Gindulyte A., Han L., He J., He S., Shoemaker B.A. (2015). PubChem Substance and Compound databases. Nucleic Acids Res..

[38] Genheden S., Ryde U. (2015). The MM/PBSA and MM/GBSA methods to estimate ligand-binding affinities. Expert. Opin. Drug Discov..

[39] Kryszak B., Biernat M., Tymowicz-Grzyb P., Junka A., Brożyna M., Worek M., Dzienny P., Antończak A., Szustakiewicz K. (2023). The effect of extrusion and injection molding on physical, chemical, and biological properties of PLLA/HAp whiskers composites. Polymer.

[40] (2009). Biological Evaluation of medical Devices. Part 5: Tests for In Vitro Cytotoxicity.

[41] Brożyna M., Paleczny J., Kozłowska W., Ciecholewska-Juśko D., Parfieńczyk A., Chodaczek G., Junka A. (2022). Chemical composition and antibacterial activity of liquid and volatile phase of essential oils against planktonic and biofilm-forming cells of *Pseudomonas aeruginosa*. Molecules.

[42] Paleczny J., Junka A.F., Krzyżek P., Czajkowska J., Kramer A., Benkhai H., Żyfka-Zagrodzińska E., Bartoszewicz M. (2023). Comparison of antibiofilm activity of low-concentrated hypochlorites vs polyhexanide-containing antiseptic. Front. Cell Infect. Microbiol..

[43] Paleczny J., Brożyna M., Dudek-Wicher R., Dydak K., Oleksy-Wawrzyniak M., Madziała M., Bartoszewicz M., Junka A. (2022). The medium composition impacts *Staphylococcus aureus* biofilm formation and susceptibility to antibiotics applied in the treatment of bone infections. Int. J. Mol. Sci..

