# Supplementary material for: The Potential Impact of Edible Fruit Extracts on Bacterial Nucleases in Preliminary Research—In Silico and In Vitro Insight
Source: Int J Mol Sci. 2025 Feb 19;26(4):1757. doi: 10.3390/ijms26041757 (PMC11855197; doi:10.3390/ijms26041757)
Supplement: Supplementary file 1 [file ijms-26-01757-s001.zip › ijms-3463143-supplementary.pdf]

The potential impact of edible fruit extracts on bacterial nucleases in preliminary research – *in silico* and *in vitro* insight

Łukasz Szeleszczuk[1,*], Malwina Brożyna[2], Bartłomiej Dudek[2], Marcin Czarnecki [3], Adam Junka[2], and Monika E. Czerwińska[4,5,*]

[1] Department of Organic and Physical Chemistry, Medical University of Warsaw, Banacha 1 Str., 02-097 Warsaw, Poland
[2] Platform for Unique Models Application, Department of Pharmaceutical Microbiology and Parasitology, Faculty of Pharmacy, Wroclaw Medical University, Borowska 211 Str., 50-556 Wroclaw, Poland
[3] Department of Infectious Diseases, Liver Diseases and Acquired Immune Deficiencies, Wroclaw Medical University, Koszarowa 5 Str., 51-149 Wroclaw, Poland; marcin.czarnecki@umw.edu.pl
[4] Department of Biochemistry and Pharmacogenomics, Medical University of Warsaw,
Banacha 1, 02-097 Warsaw, Poland
[5] Centre for Preclinical Research, Medical University of Warsaw, 1B Banacha Str., 02-097 Warsaw, Poland

Corresponding author:
Łukasz Szeleszczuk, e-mail address lukasz.szeleszczuk@wum.edu.pl; Monika E. Czerwińska, e-mail address: monika.czerwinska@wum.edu.pl

Table S1. Results of the molecular docking and MM/GBSA calculations for colicin E9. All of the values are in kcal/mol.

	Docking score	ΔG	CE	CB	HB	LE	PP	GB	SA
Procyanidin B2	-9.31	-69.43	-36.12	8.65	-6.54	-13.96	-2.33	33.59	-52.72
Cornuside	-9.18	-56.75	-175.94	8.08	-5.09	-11.11	-2.16	182.41	-52.96
Isorhamnetin-3-*O*-*β*-D-glucosyl-7-*O*-*α*-L-rhamnoside	-8.93	-55.13	-163.77	3.21	-5.26	-9.22	-2.43	174.38	-52.04
Isorhamnetin-3-*O*-rutinoside	-8.67	-52.25	-133.26	7.35	-5.10	-15.40	-4.06	137.08	-38.86
Loganic acid	-8.60	-51.39	-38.88	6.76	-3.97	-9.88	-6.55	42.17	-41.05
Isorhamnetin 3-*O*-*β*-D-glucoside	-8.30	-50.77	-36.18	8.27	-3.87	-11.82	0.00	33.47	-40.64
Procyanidin C1	-8.11	-50.65	-34.64	8.09	-4.05	-14.65	-1.35	20.85	-24.90
Epicatechin	-5.97	-48.55	-171.83	5.74	-6.82	-10.57	-3.23	177.61	-39.46

CE: Coulomb energy; CB: covalent binding energy; HB: hydrogen bonding correction; LE: lipophilic energy; PP: pi-pi packing correction; GB: generalized Born electrostatic solvation energy; SA: surface area electrostatic solvation energy.

Table S2. Results of the molecular docking and MM/GBSA calculations for endonuclease 1. All of the values are in kcal/mol.

	Docking score	ΔG	CE	CB	HB	LE	PP	GB	SA
Isorhamnetin-3-*O*-rutinoside	-10.33	-48.33	-39.65	12.19	-3.75	-7.86	-0.40	43.85	-52.71
Cornuside	-9.47	-46.36	-146.55	13.69	-5.34	-12.87	-3.34	151.88	-43.83
Loganic acid	-8.23	-43.35	-17.67	8.86	-0.96	-16.04	0.00	24.12	-41.66
Isorhamnetin-3-*O*-*β*-D-glucosyl-7-*O*-*α*-L-rhamnoside	-7.92	-42.05	-215.25	3.55	-3.11	-16.35	-2.63	229.20	-37.46
Procyanidin C1	-7.43	-41.77	-140.83	1.39	-2.79	-9.51	-2.45	144.06	-31.63
Isorhamnetin 3-*O*-*β*-D-glucoside	-7.15	-41.63	-22.00	8.14	-2.57	-17.84	0.00	23.05	-30.41
Procyanidin B2	-6.05	-41.40	-23.27	9.20	-2.74	-11.45	-1.46	32.37	-44.06
Epicatechin	-5.77	-38.50	-22.57	19.05	-3.39	-15.27	-1.88	30.91	-45.35

CE: Coulomb energy; CB: covalent binding energy; HB: hydrogen bonding correction; LE: lipophilic energy; PP: pi-pi packing correction; GB: generalized Born electrostatic solvation energy; SA: surface area electrostatic solvation energy.

Table S3. Results of the molecular docking and MM/GBSA calculations for ribonuclease H. All of the values are in kcal/mol.

	Docking score	ΔG	CE	CB	HB	LE	PP	GB	SA
Procyanidin B2	-14.42	-32.90	-162.76	7.46	-2.95	-6.67	-4.06	188.83	-52.73
Cornuside	-12.01	-32.57	-57.02	24.81	-6.66	-17.39	-0.42	55.50	-31.40
Isorhamnetin-3-*O*-rutinoside	-11.85	-31.45	49.60	2.13	-2.97	-6.27	0.00	-47.23	-26.70
Isorhamnetin 3-*O*-*β*-D-glucoside	-10.88	-31.19	-22.56	7.42	-2.00	-15.95	-0.38	30.69	-28.42

Isorhamnetin-3-*O*-*β*-D-glucosyl-7-*O*-*α*-L-rhamnoside	-9.37	-30.52	-304.90	9.95	-3.40	-7.27	-2.13	316.82	-39.59
Loganic acid	-9.30	-30.11	-312.17	4.43	-3.79	-12.59	-2.81	340.10	-43.28
Epicatechin	-6.43	-30.02	-31.06	11.51	-4.83	-6.88	-1.40	47.37	-44.73
Procyanidin C1	The interaction has not been established.								

CE: Coulomb energy; CB: covalent binding energy; HB: hydrogen bonding correction; LE: lipophilic energy; PP: pi-pi packing correction; GB: generalized Born electrostatic solvation energy; SA: surface area electrostatic solvation energy.

Table S4. Results of the molecular docking and MM/GBSA calculations for thermonuclease. All of the values are in kcal/mol.

	**Docking score**	**ΔG**	**CE**	**CB**	**HB**	**LE**	**PP**	**GB**	**SA**
Isorhamnetin-3-*O*-rutinoside	-10.44	-27.55	-85.07	6.97	-3.23	-8.84	-0.44	101.81	-38.76
Epicatechin	-9.35	-26.93	-60.60	13.54	-3.21	-10.99	0.00	68.93	-34.59
Isorhamnetin 3-*O*-*β*-D-glucoside	-9.28	-24.76	-173.26	6.49	-4.70	-5.84	-0.81	186.45	-33.09
Loganic acid	-8.12	-23.37	-322.48	5.93	-5.07	-4.33	-1.10	333.91	-30.22
Procyanidin B2	-8.08	-21.74	-51.62	6.48	-7.58	-2.98	0.00	68.97	-35.01
Isorhamnetin-3-*O*-*β*-D-glucosyl-7-*O*-*α*-L-rhamnoside	-7.63	-20.71	-130.18	6.97	-5.65	-4.86	-1.09	138.01	-23.92
Procyanidin C1	-7.55	-20.45	7.63	6.78	-3.84	-8.10	-1.06	14.53	-36.39
Cornuside	-6.38	11.34	-188.72	8.16	-5.63	-5.83	-1.83	244.08	-38.89

CE: Coulomb energy; CB: covalent binding energy; HB: hydrogen bonding correction; LE: lipophilic energy; PP: pi-pi packing correction; GB: generalized Born electrostatic solvation energy; SA: surface area electrostatic solvation energy.

Table S5. Results of the molecular docking and MM/GBSA calculations for nuclease SbcCD subunit C. All of the values are in kcal/mol.

	**Docking score**	**ΔG**	**CE**	**CB**	**HB**	**LE**	**PP**	**GB**	**SA**
Isorhamnetin-3-*O*-*β*-D-glucosyl-7-*O*-*α*-L-rhamnoside	-8.99	-38.44	-176.54	7.10	-5.52	-6.79	0.00	168.56	-25.25
Isorhamnetin 3-*O*-*β*-D-glucoside	-8.32	-37.86	-184.14	6.38	-5.00	-6.59	-0.02	186.36	-34.85
Epicatechin	-7.21	-36.96	-114.34	6.34	-3.49	-5.83	0.00	108.54	-28.18
Isorhamnetin-3-*O*-rutinoside	-6.93	-36.10	-121.35	7.12	-3.17	-10.57	-1.85	132.30	-38.57
Procyanidin B2	-6.38	-36.09	-33.42	7.97	-3.81	-10.16	-2.25	39.41	-33.83
Cornuside	-6.32	-35.76	-566.83	9.84	-5.51	-7.15	-0.97	572.86	-38.00
Loganic acid	-5.46	-35.69	-97.92	5.44	-3.52	-7.16	-0.32	105.92	-38.12
Procyanidin C1	The complex has not been obtained.								

CE: Coulomb energy; CB: covalent binding energy; HB: hydrogen bonding correction; LE: lipophilic energy; PP: pi-pi packing correction; GB: generalized Born electrostatic solvation energy; SA: surface area electrostatic solvation energy.

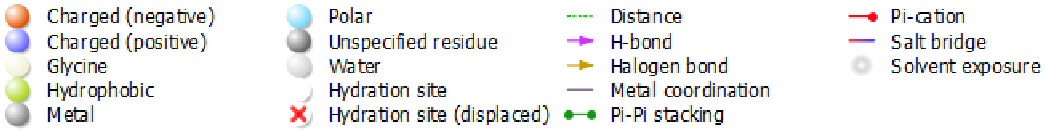

Figure S1. Legend for protein-compound interaction diagrams, presented below.

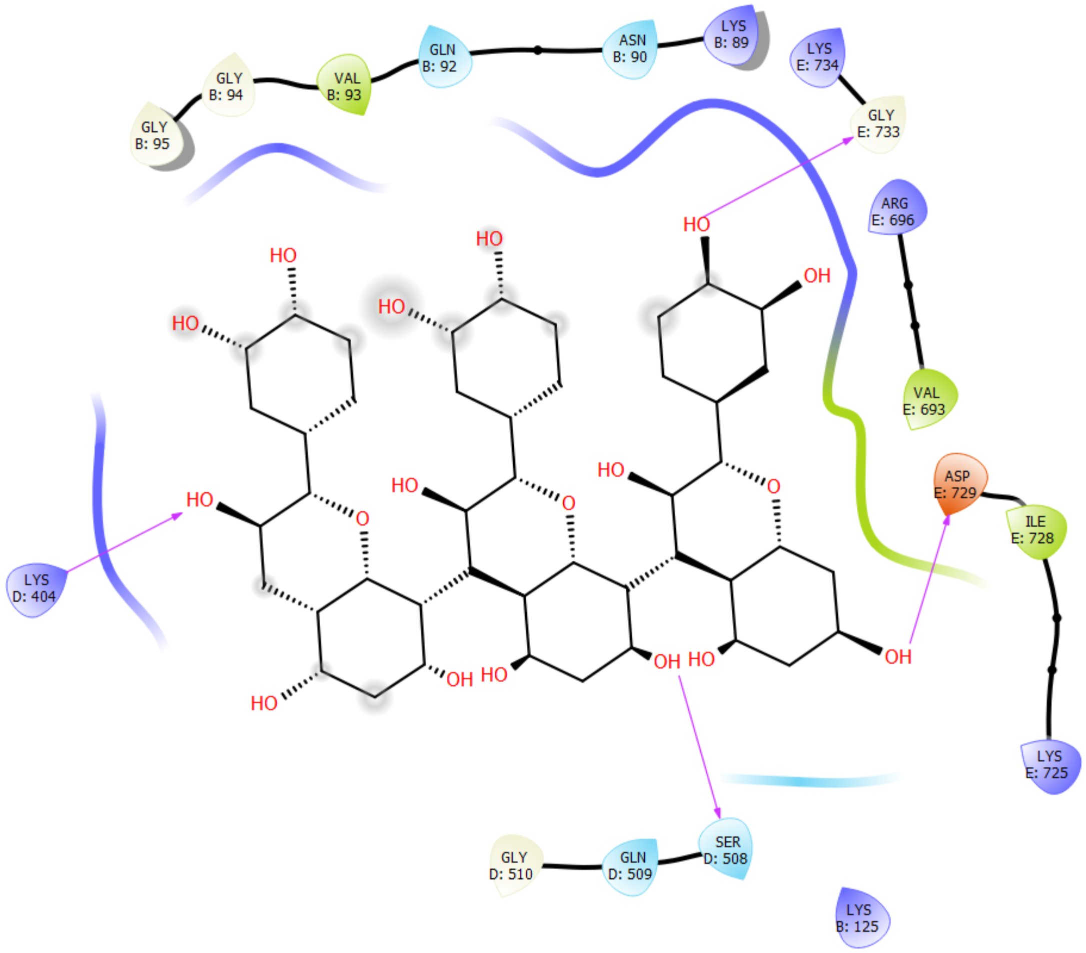

Figure S2. Protein-compound interaction diagram formed between procyanidin C1 and colicin E9.

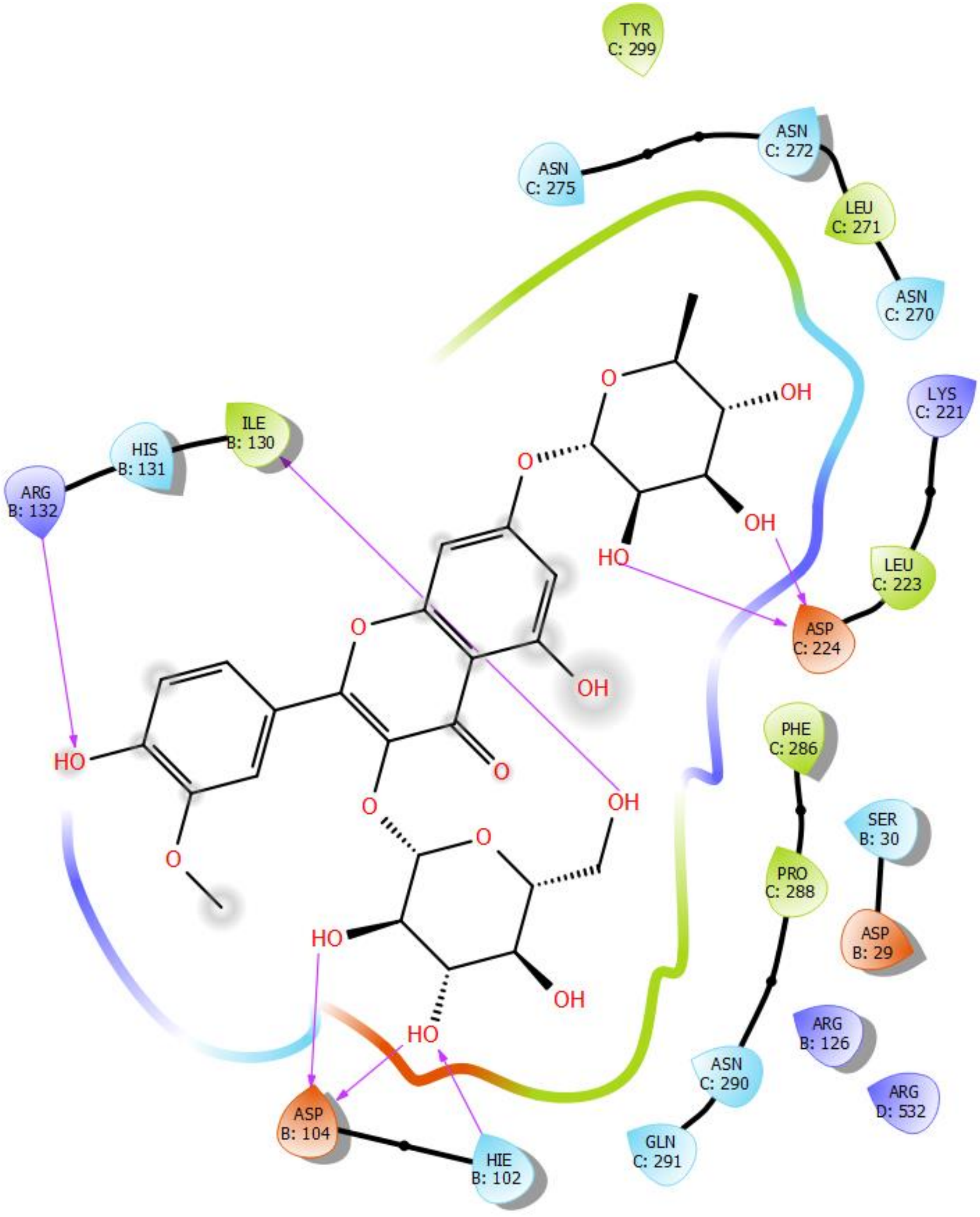

Figure S3. Protein-compound interaction diagram formed between isorhamnetin-3-*O*-*β*-D-glucosyl-7-*O*-*α*-L-rhamnoside and colicin E9.

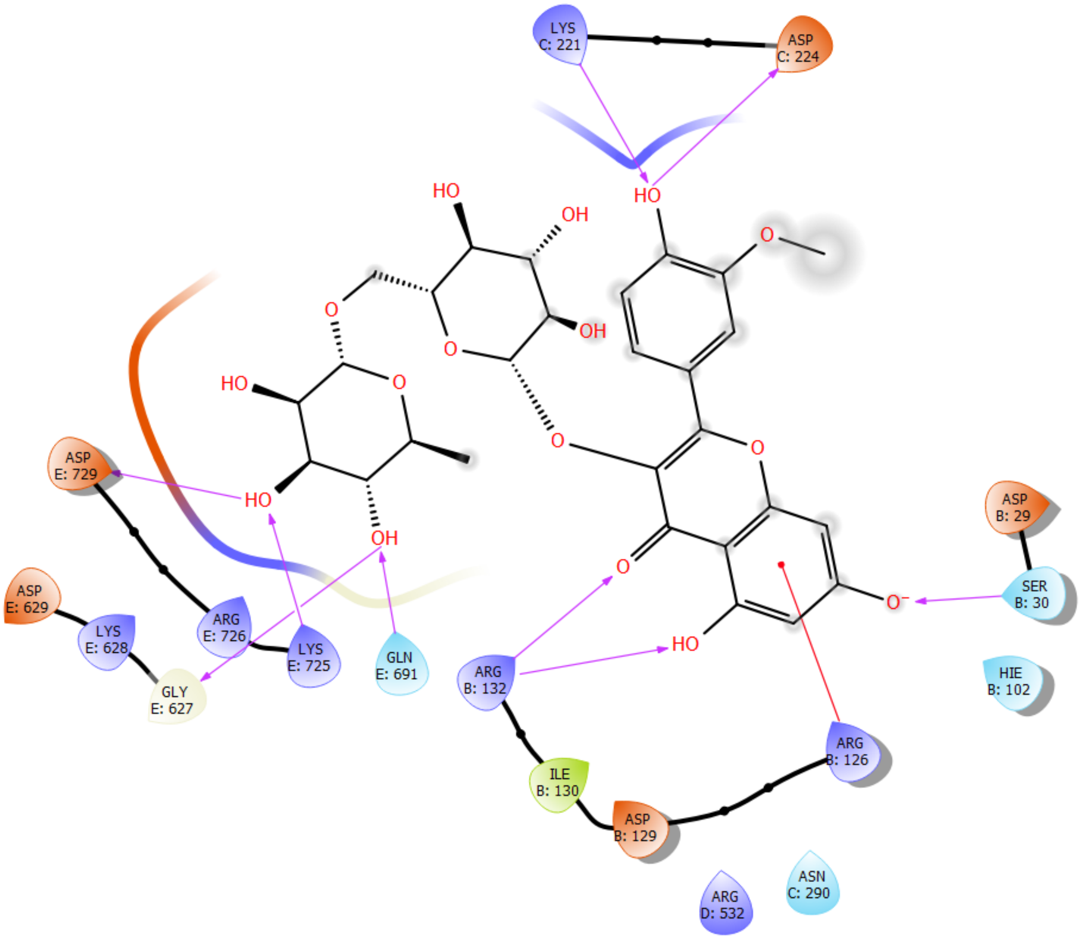

Figure S4. Protein-compound interaction diagram formed between isorhamnetin-3-*O*-rutinoside and colicin E9.

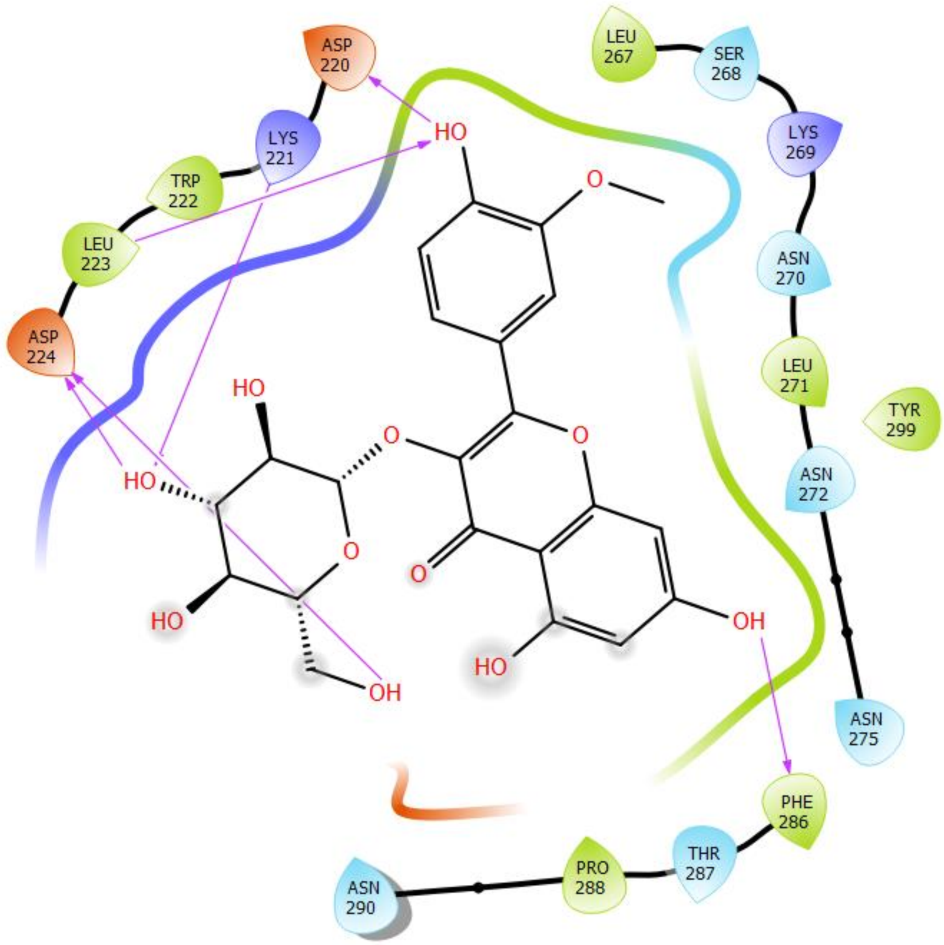

Figure S5. Protein-compound interaction diagram formed between isorhamnetin 3-*O*-*β*-D-glucoside and colicin E9.

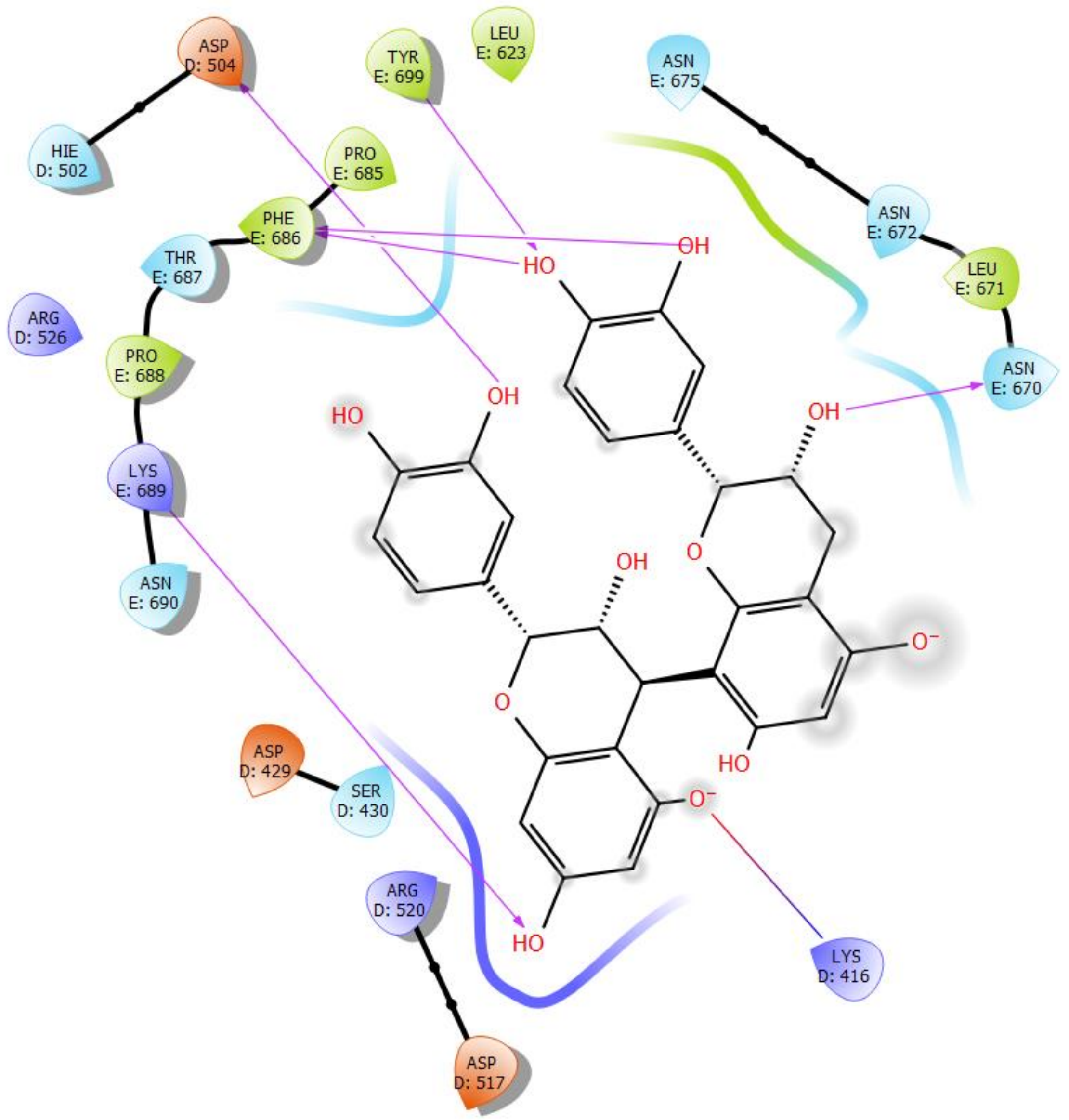

Figure S6. Protein-compound interaction diagram formed between procyanidin B2 and colicin E9.

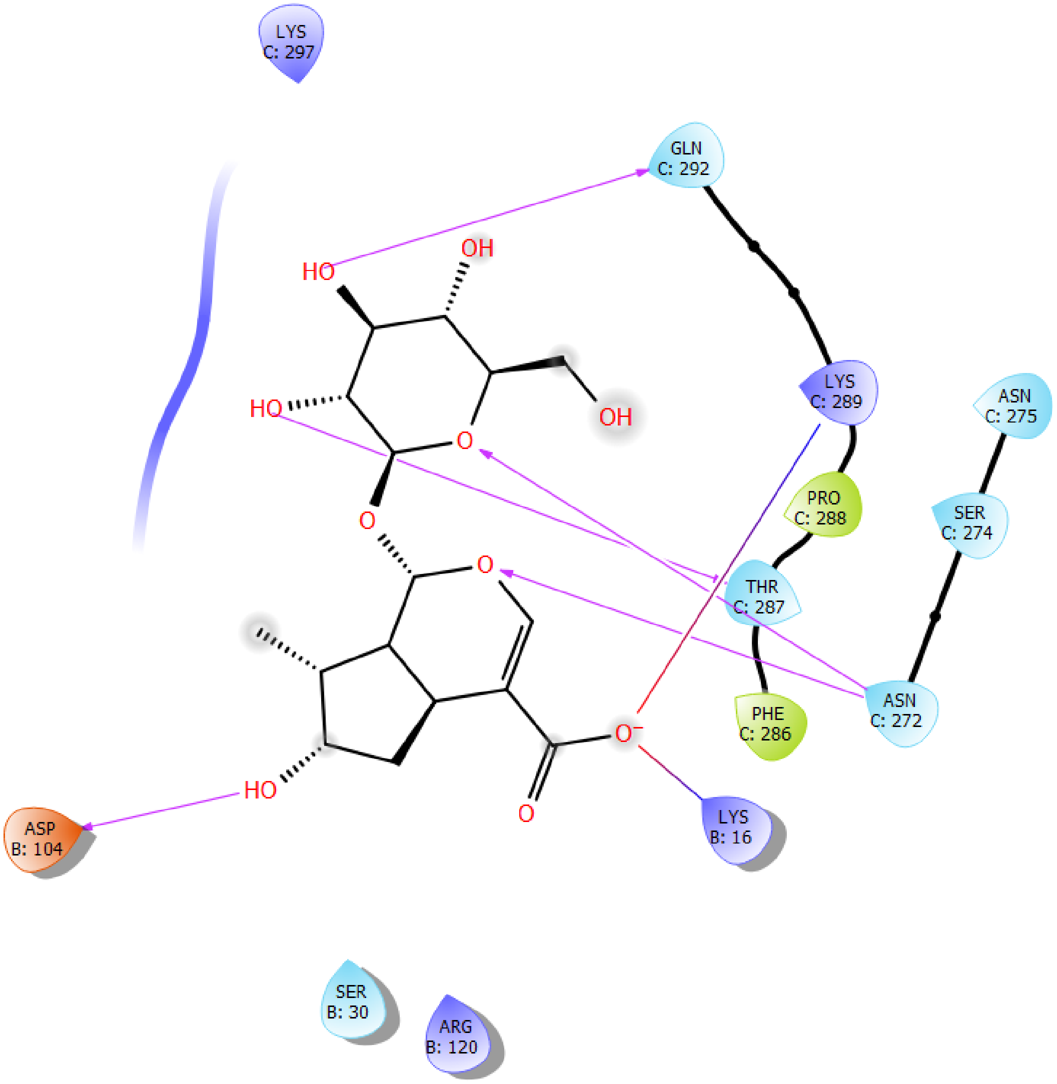

Figure S7. Protein-compound interaction diagram formed between loganic acid and colicin E9.

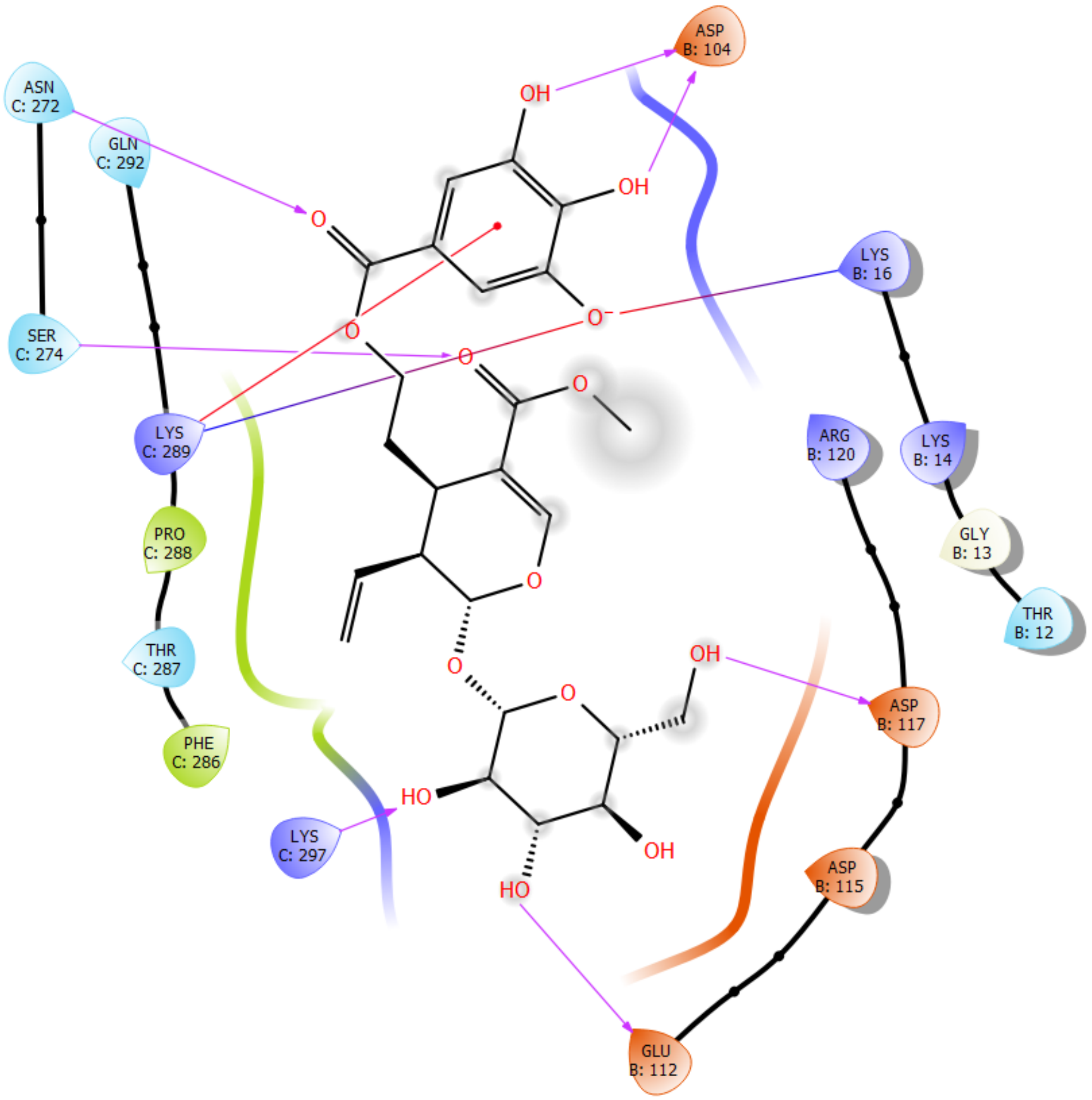

Figure S8. Protein-compound interaction diagram formed between cornuside and colicin E9.

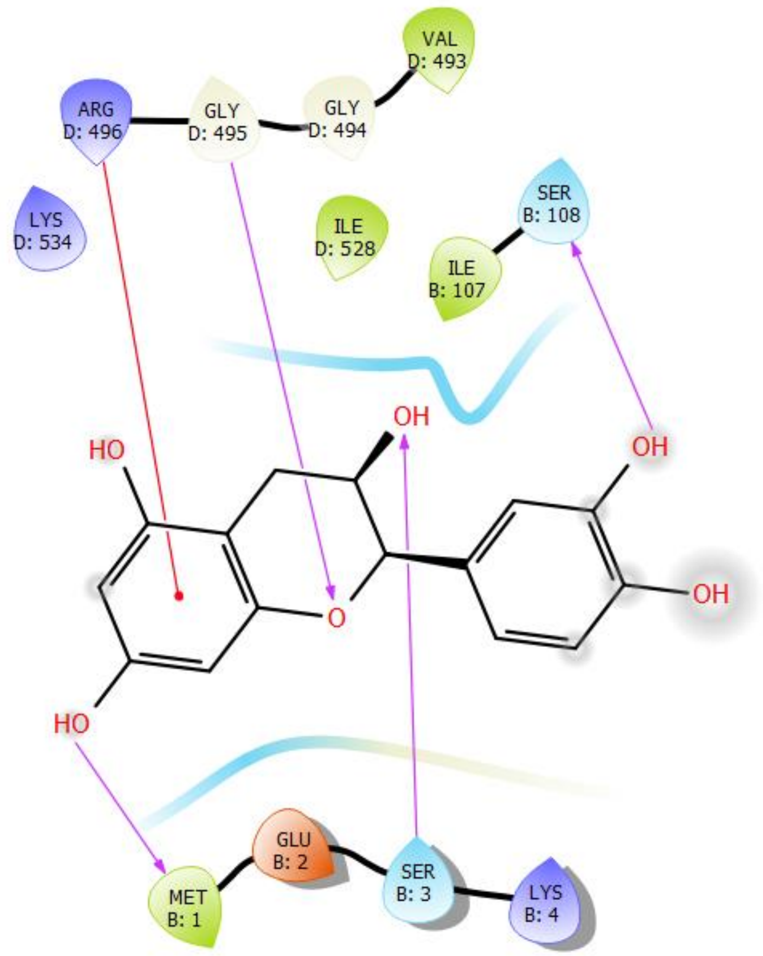

Figure S9. Protein-compound interaction diagram formed between epicatechin and colicin E9.

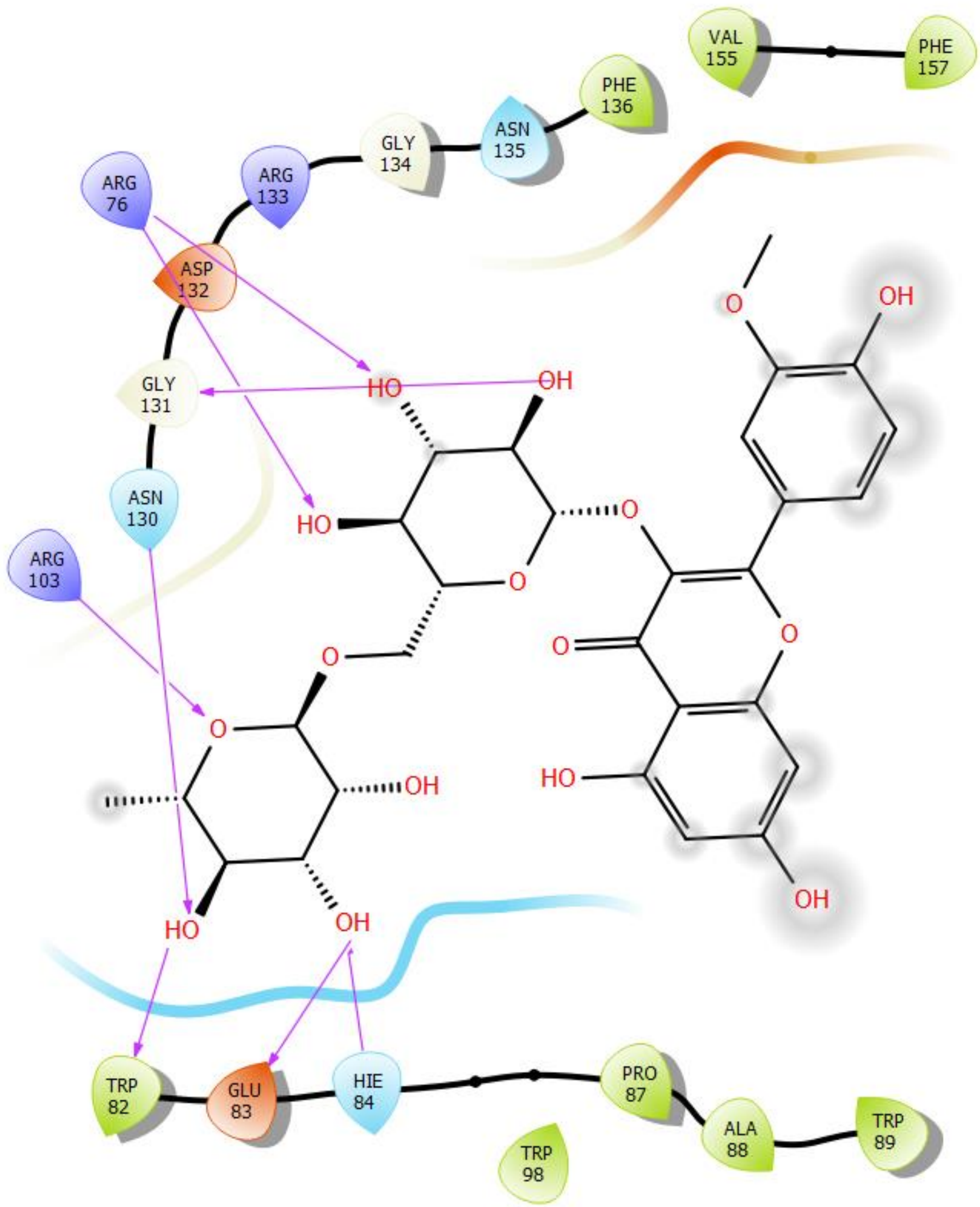

Figure S10. Protein-compound interaction diagram formed between isorhamnetin-3-*O*-rutinoside and endonuclease 1.

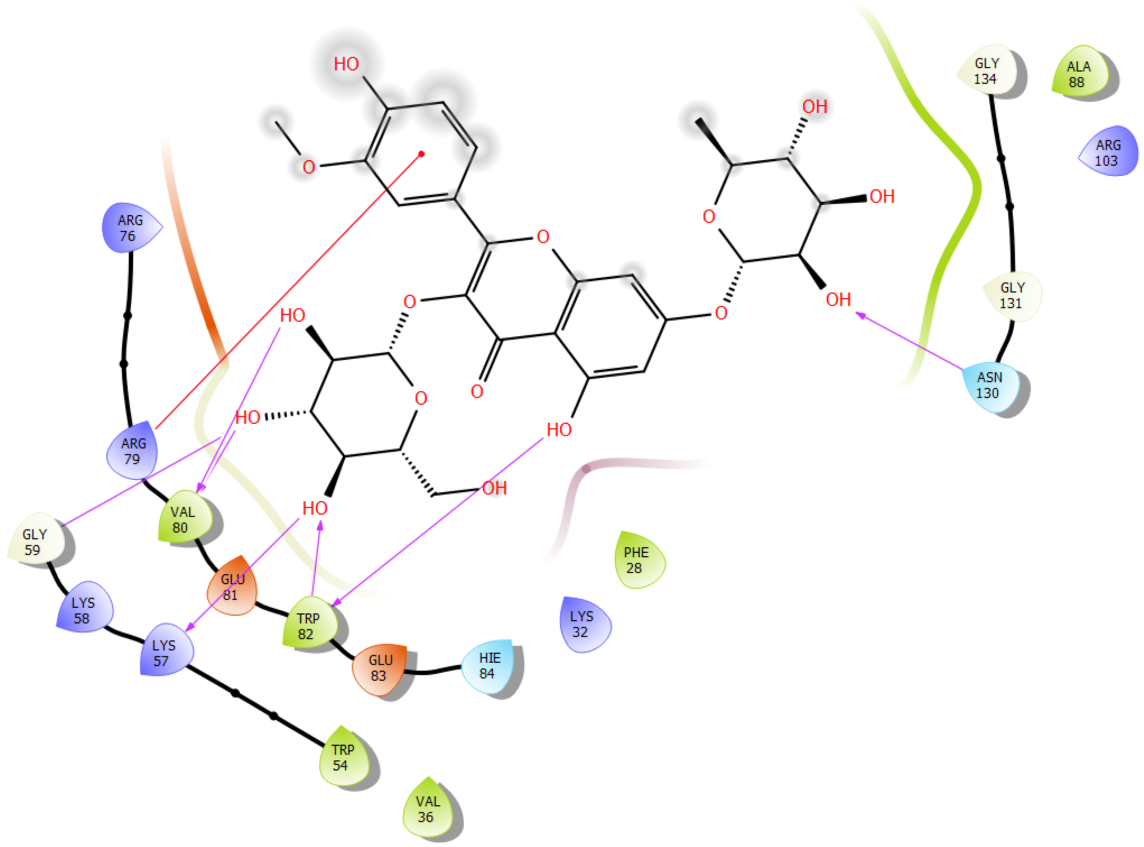

Figure S11. Protein-compound interaction diagram formed between isorhamnetin-3-*O*-*β*-D-glucosyl-7-*O*-*α*-L-rhamnoside and endonuclease 1.

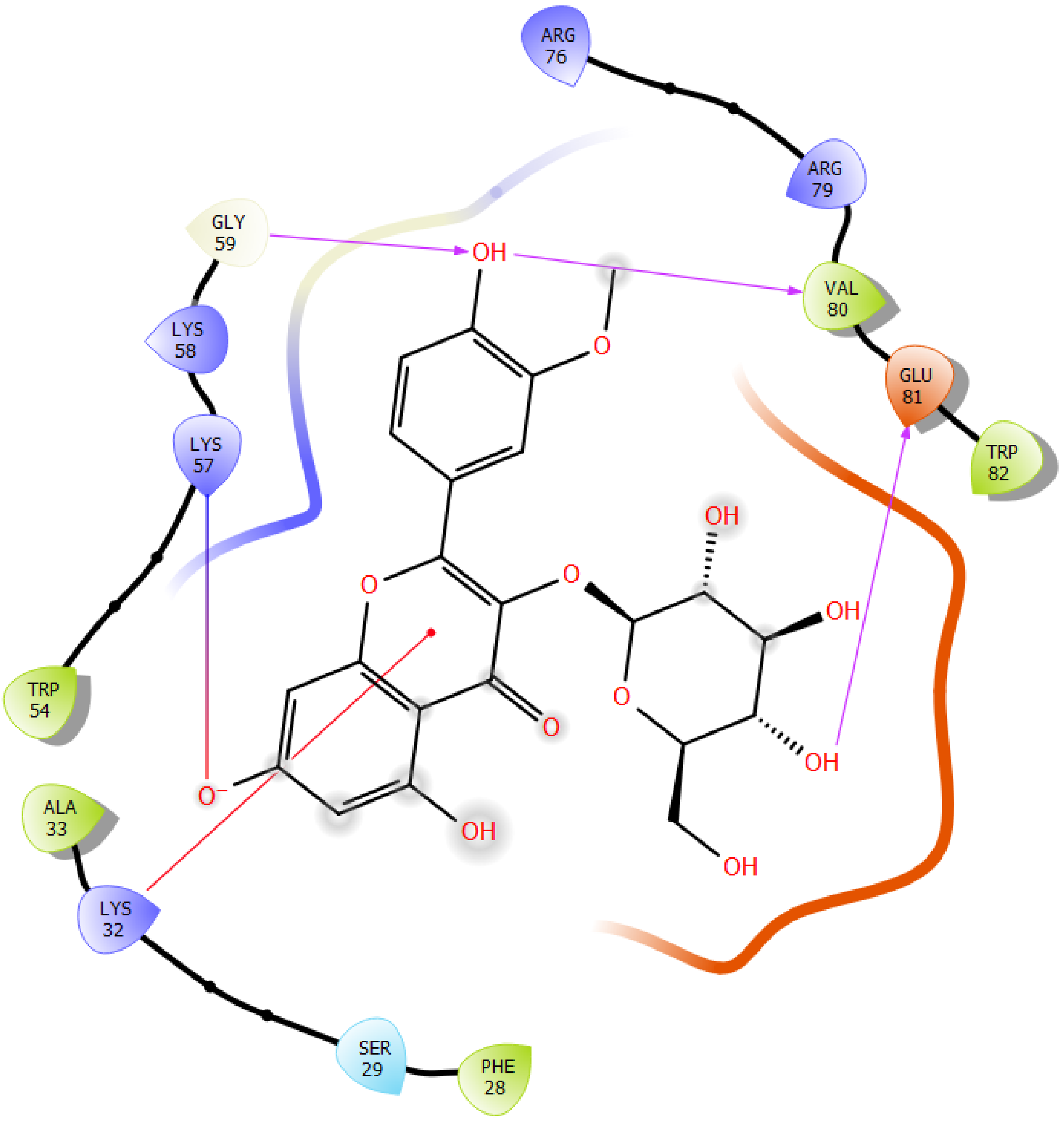

Figure S12. Protein-compound interaction diagram formed between isorhamnetin 3-*O*-*β*-D-glucoside and endonuclease 1.

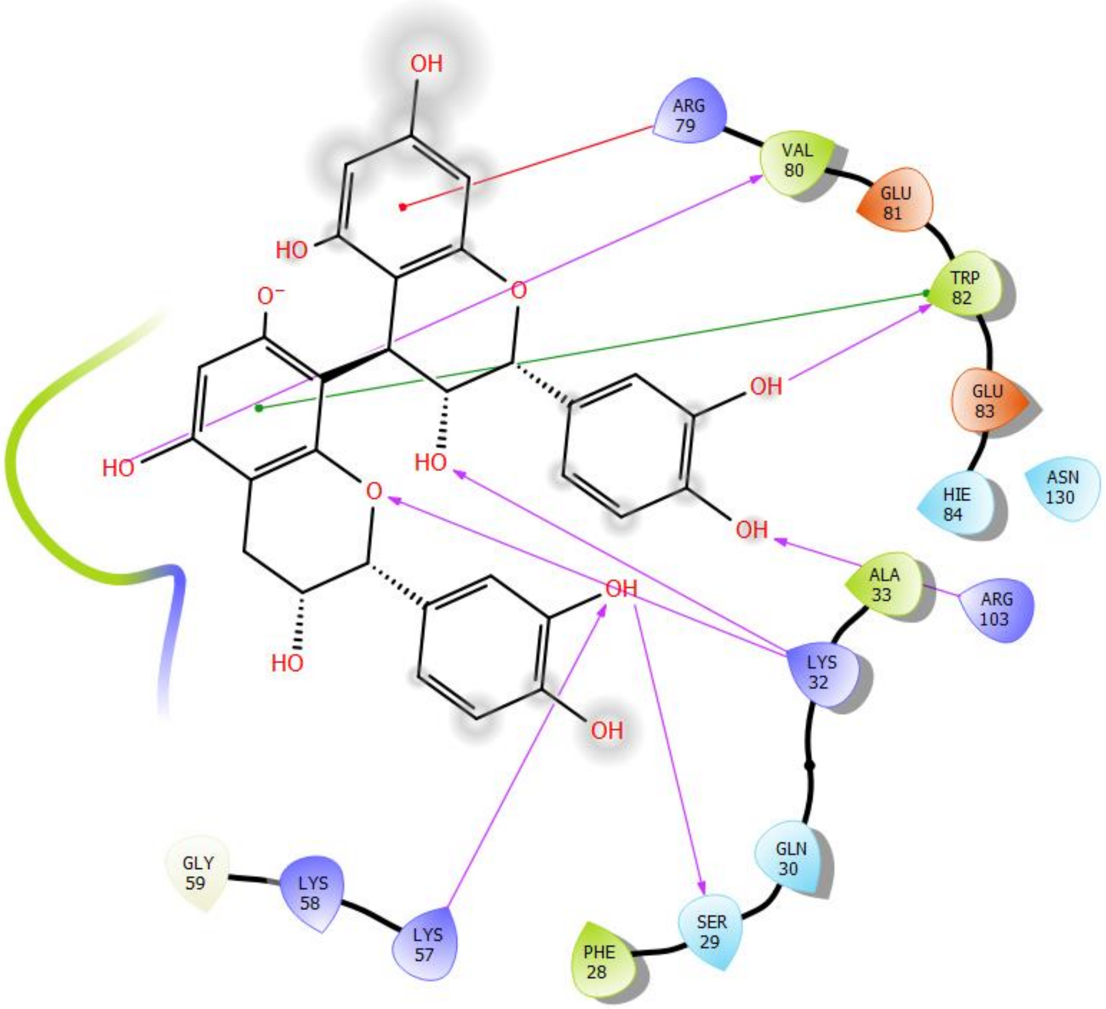

Figure S13. Protein-compound interaction diagram formed between procyanidin B2 and endonuclease 1.

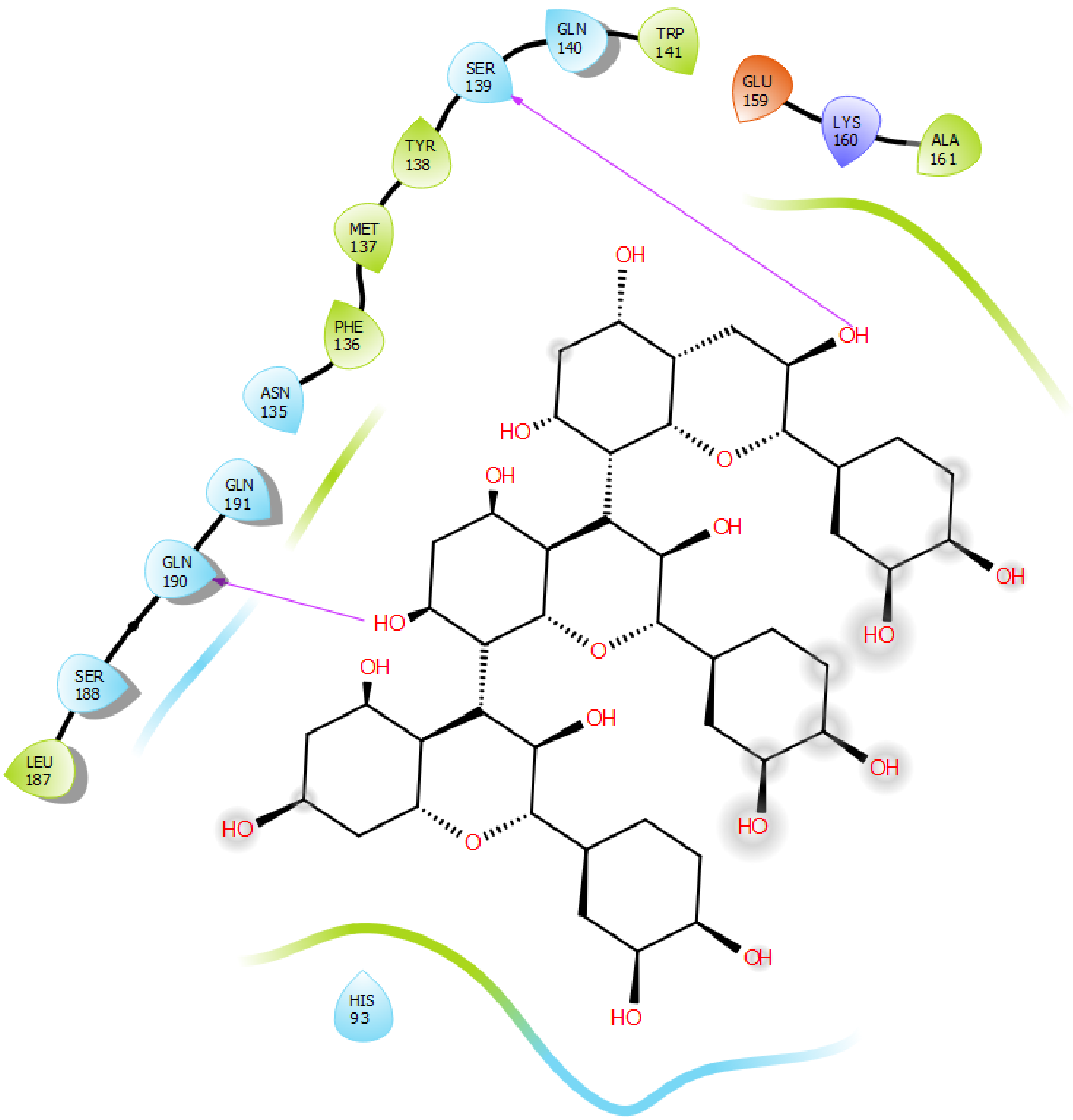

Figure S14. Protein-compound interaction diagram formed between procyanidin C1 and endonuclease 1.

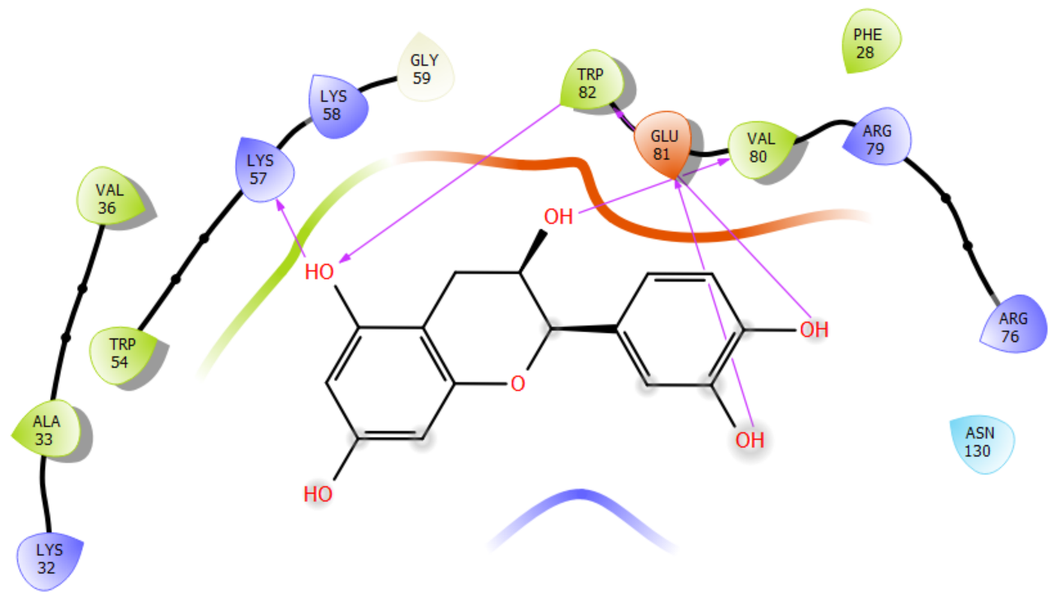

Figure S15. Protein-compound interaction diagram formed between epicatechin and endonuclease 1.

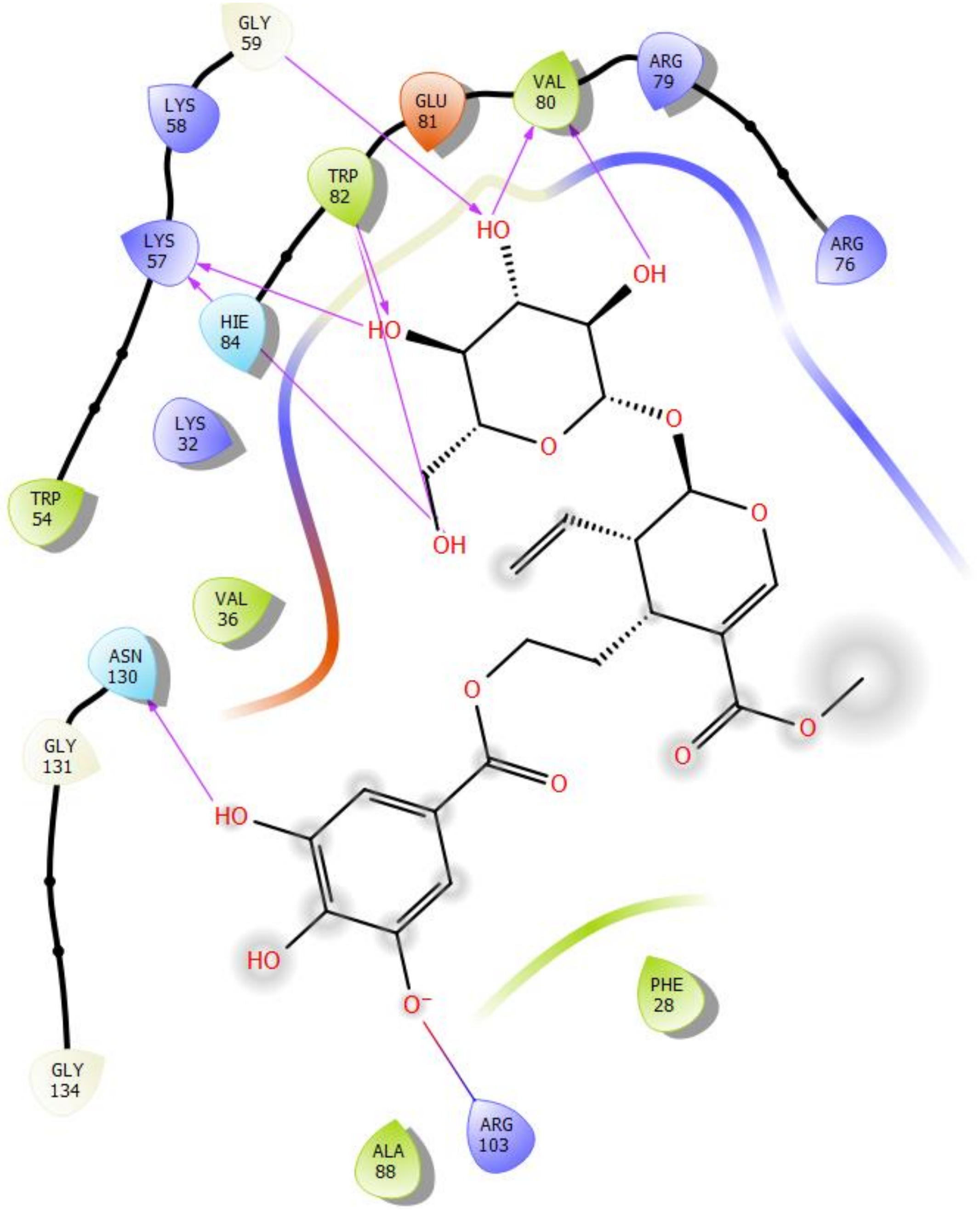

Figure S16. Protein-compound interaction diagram formed between cornuside and endonuclease 1.

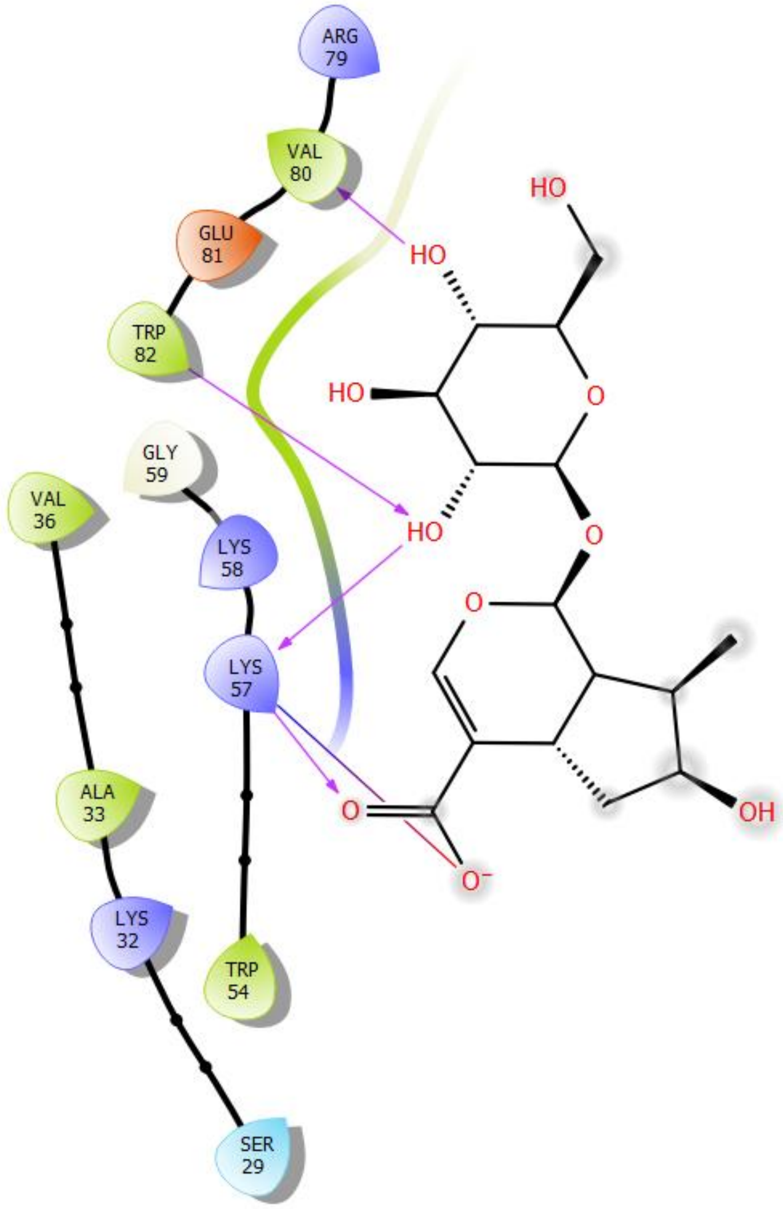

Figure S17. Protein-compound interaction diagram formed between loganic acid and endonuclease 1.

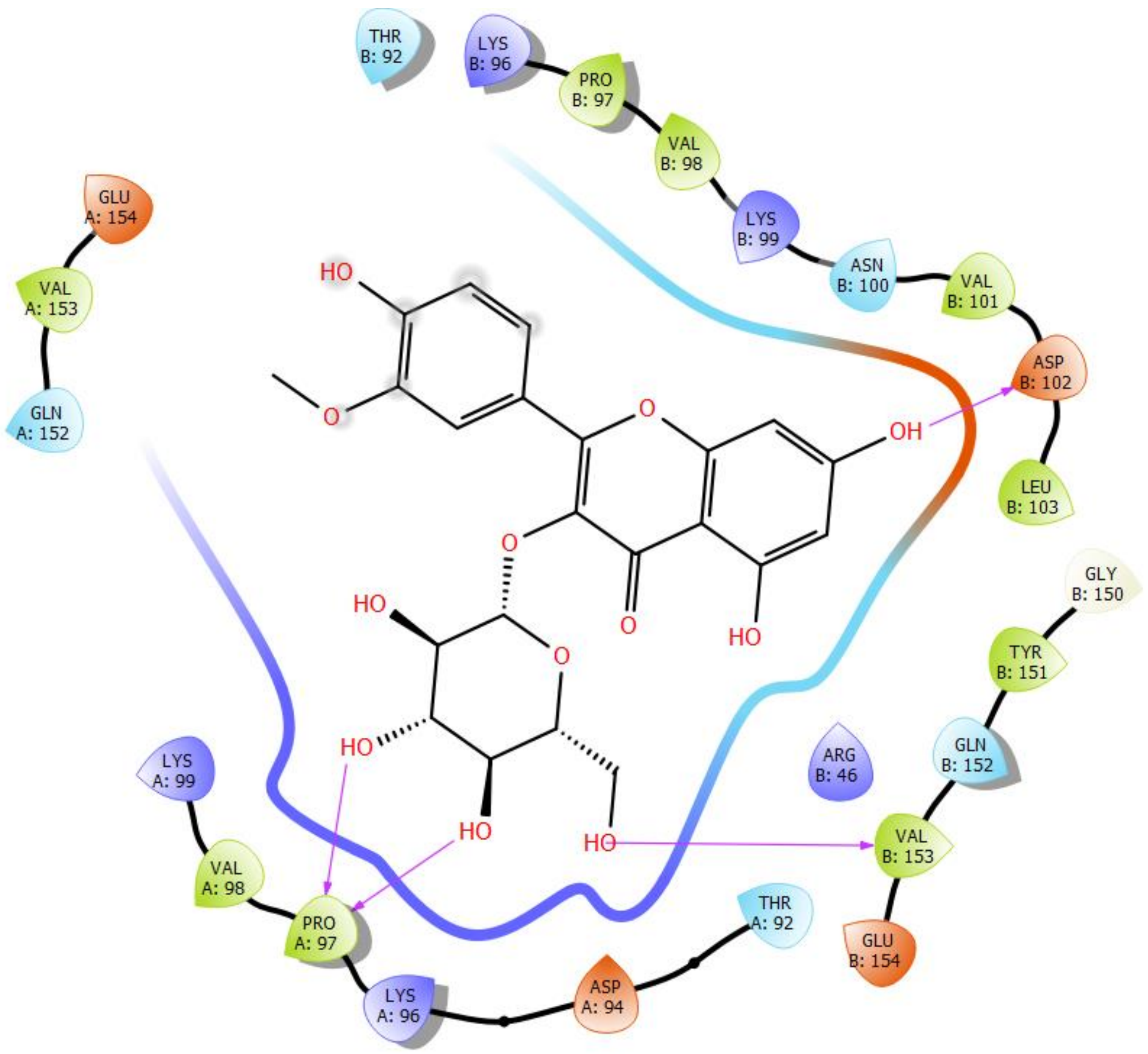

Figure S18. Protein-compound interaction diagram formed between isorhamnetin 3-*O*-*β*-D-glucoside and ribonuclease H.

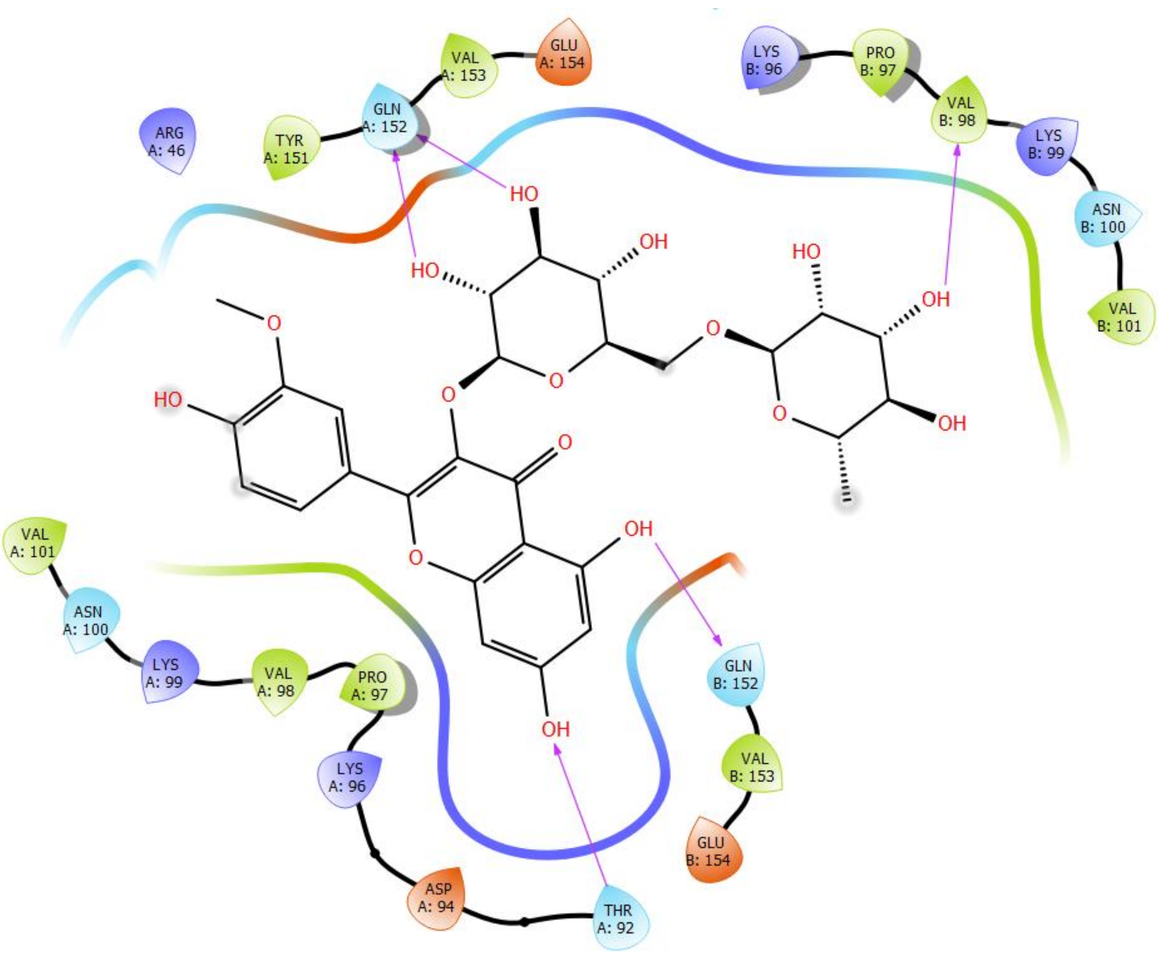

Figure S19. Protein-compound interaction diagram formed between isorhamnetin-3-*O*-rutinoside and ribonuclease H.

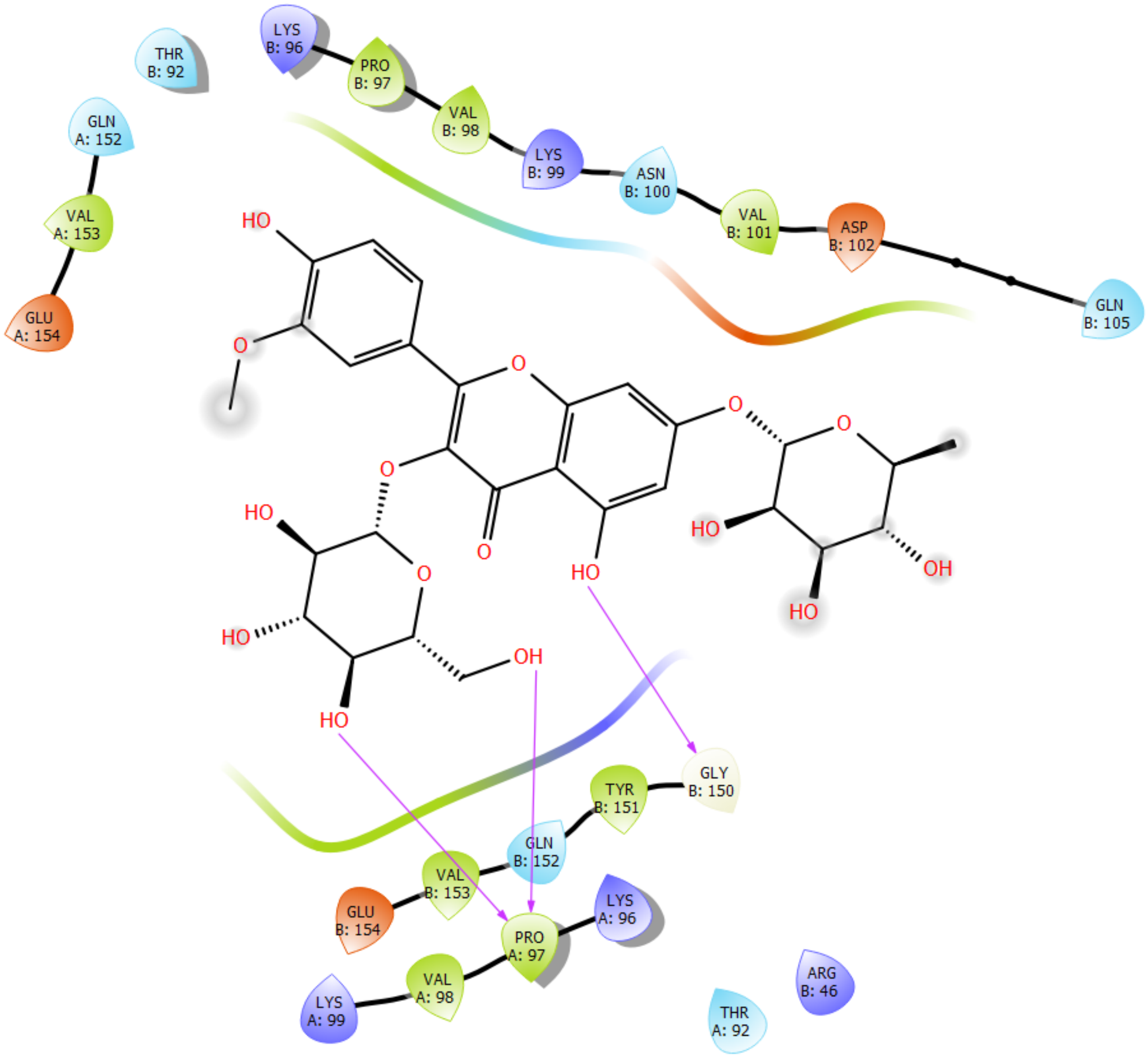

Figure S20. Protein-compound interaction diagram formed between isorhamnetin-3-*O*-*β*-D-glucosyl-7-*O*-*α*-L-rhamnoside and ribonuclease H.

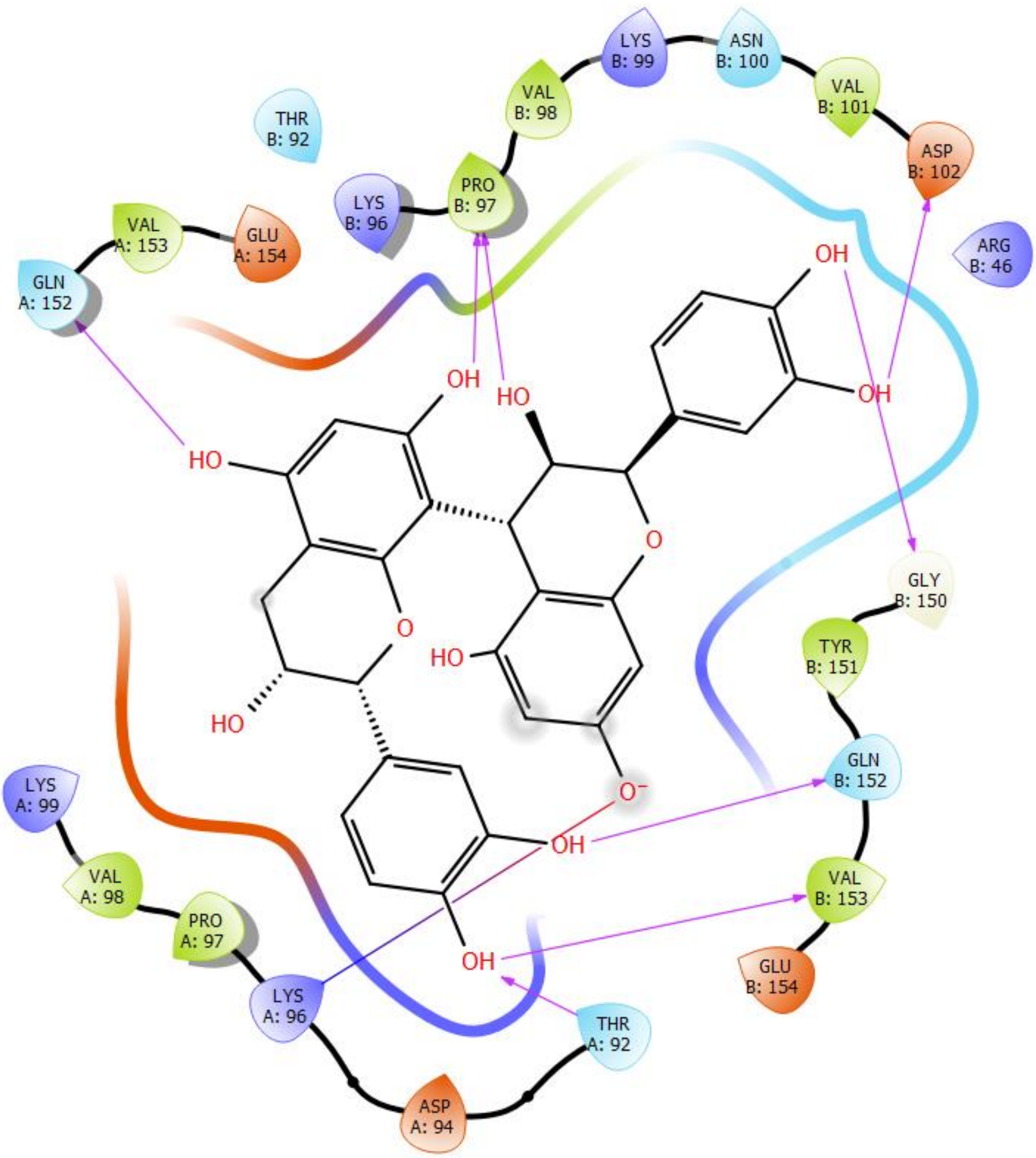

Figure S21. Protein-compound interaction diagram formed between procyanidin B2 and ribonuclease H.

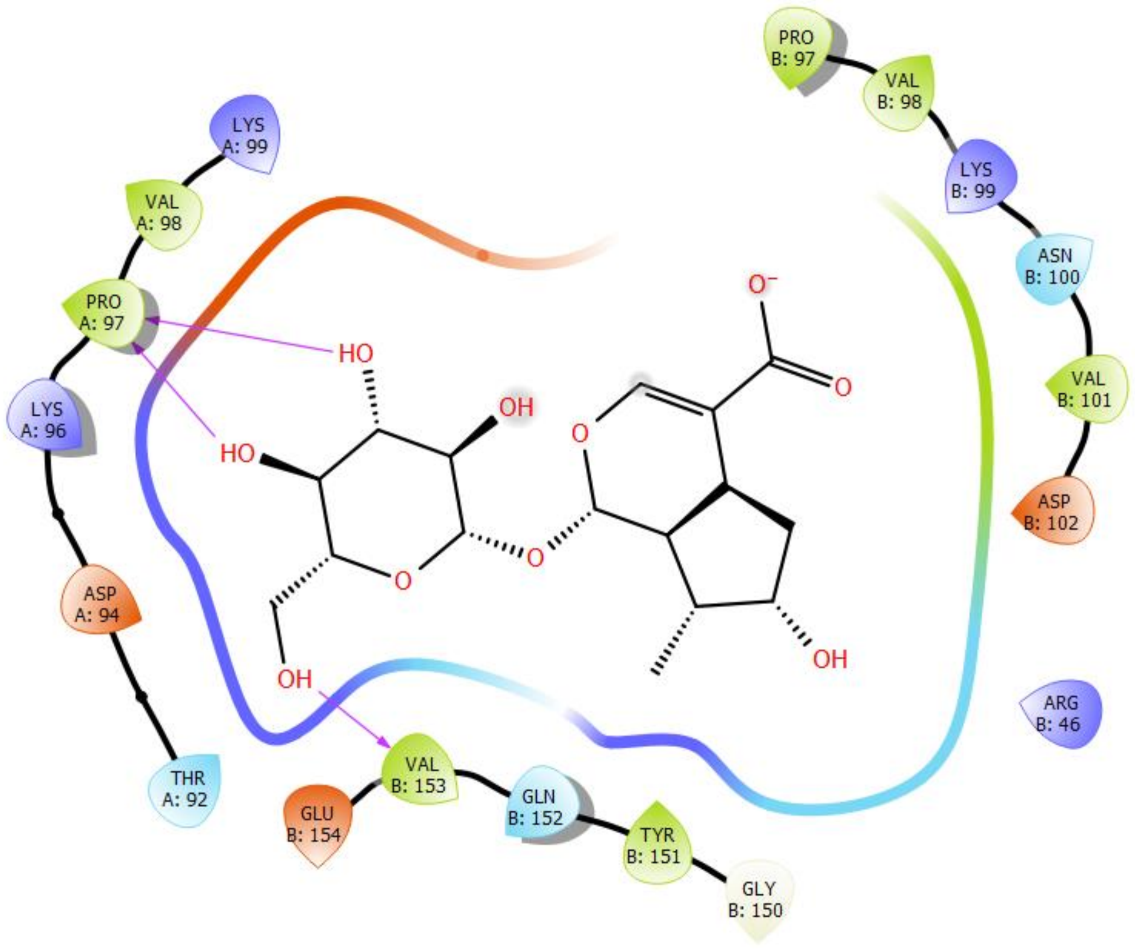

Figure S22. Protein-compound interaction diagram formed between loganic acid and ribonuclease H.

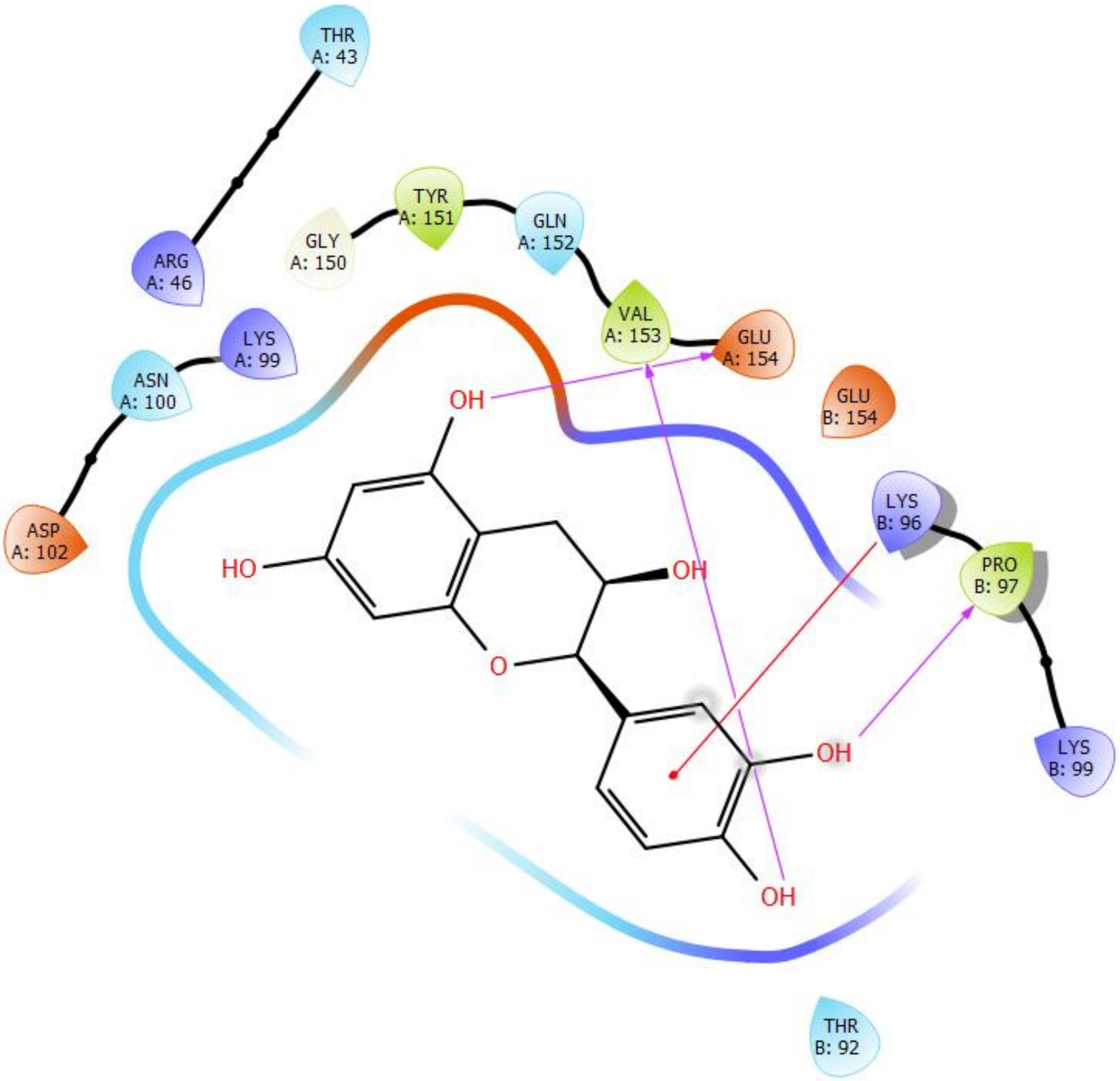

Figure S23. Protein-compound interaction diagram formed between epicatechin and ribonuclease H.

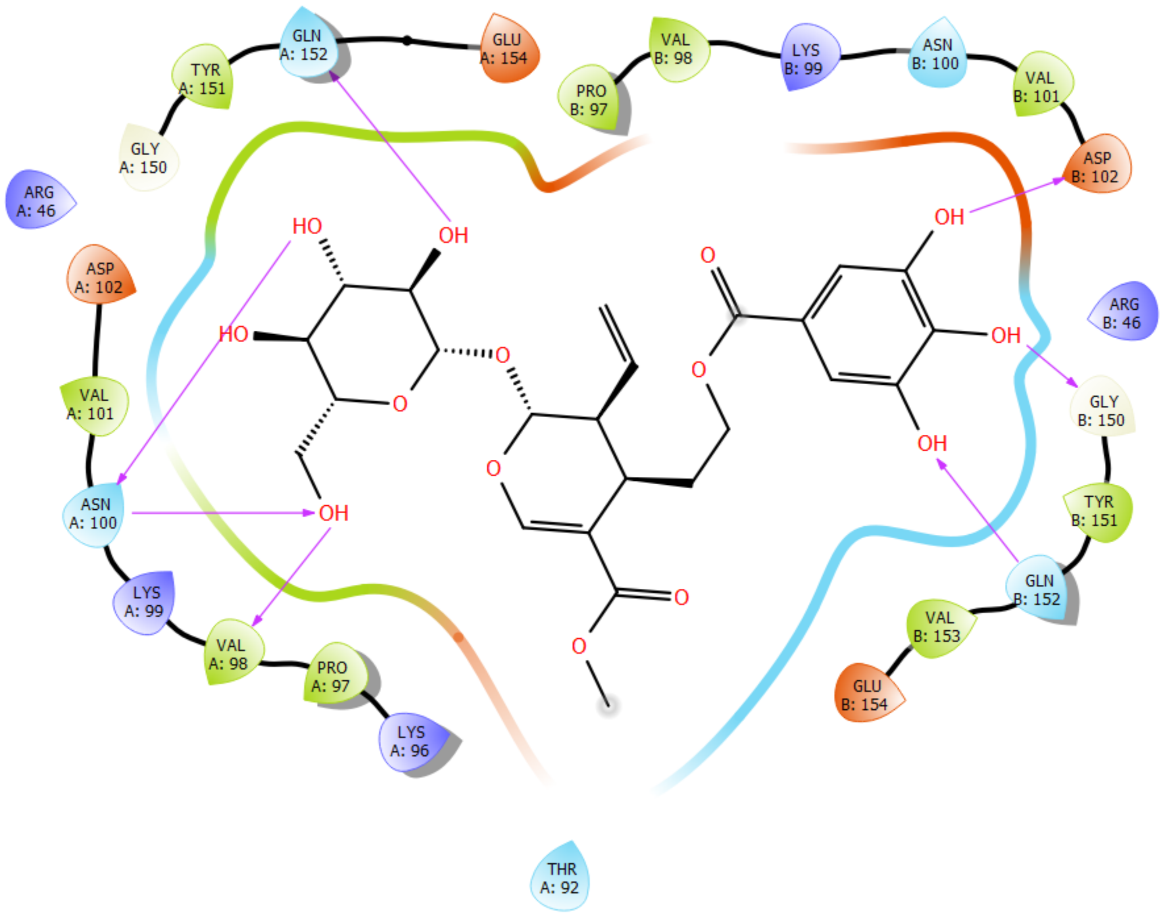

Figure S24. Protein-compound interaction diagram formed between cornuside and ribonuclease H.

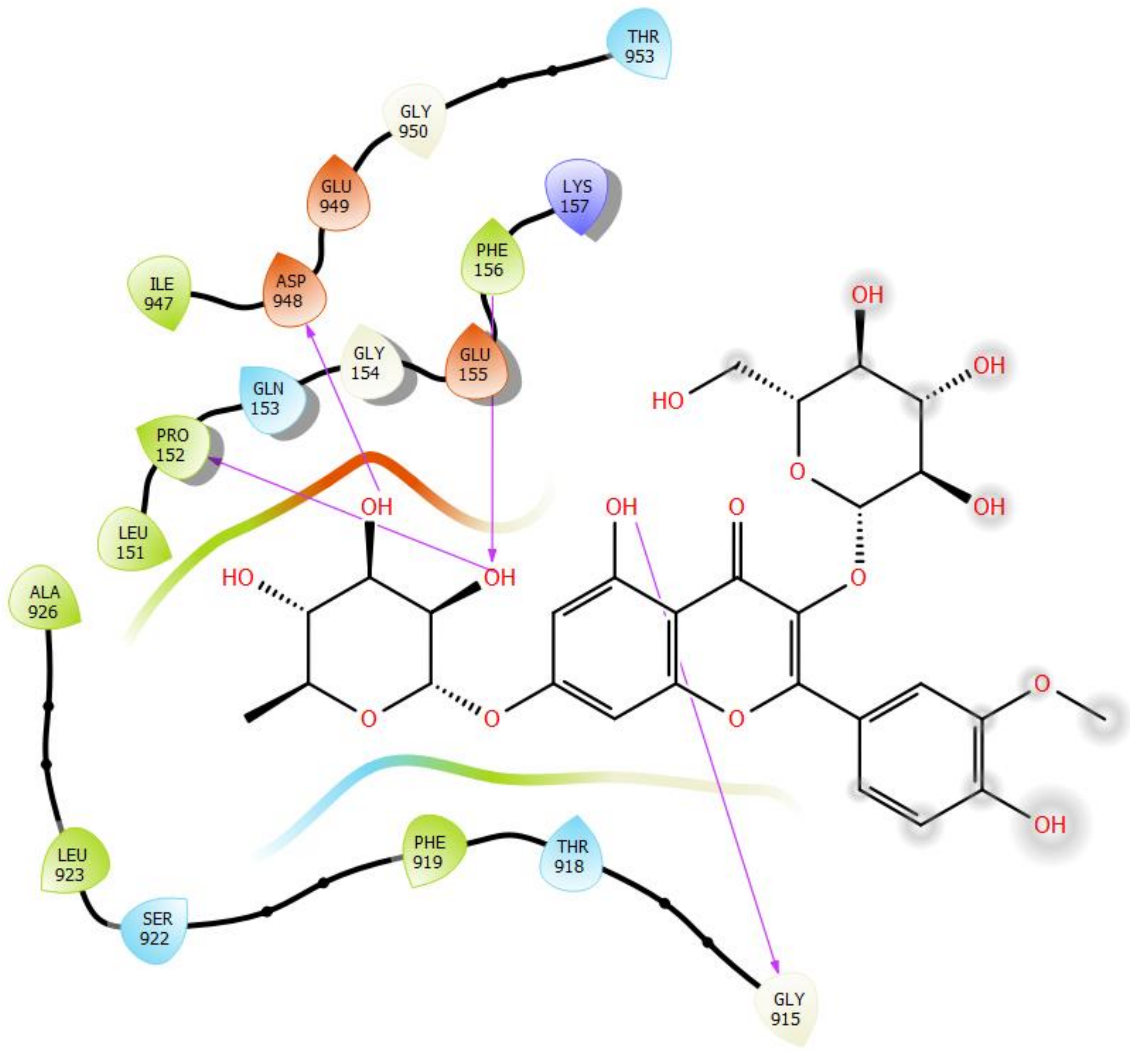

Figure S25. Protein-compound interaction diagram formed between isorhamnetin-3-*O*-*β*-D-glucosyl-7-*O*-*α*-L-rhamnoside and nuclease SbcCD subunit C.

.

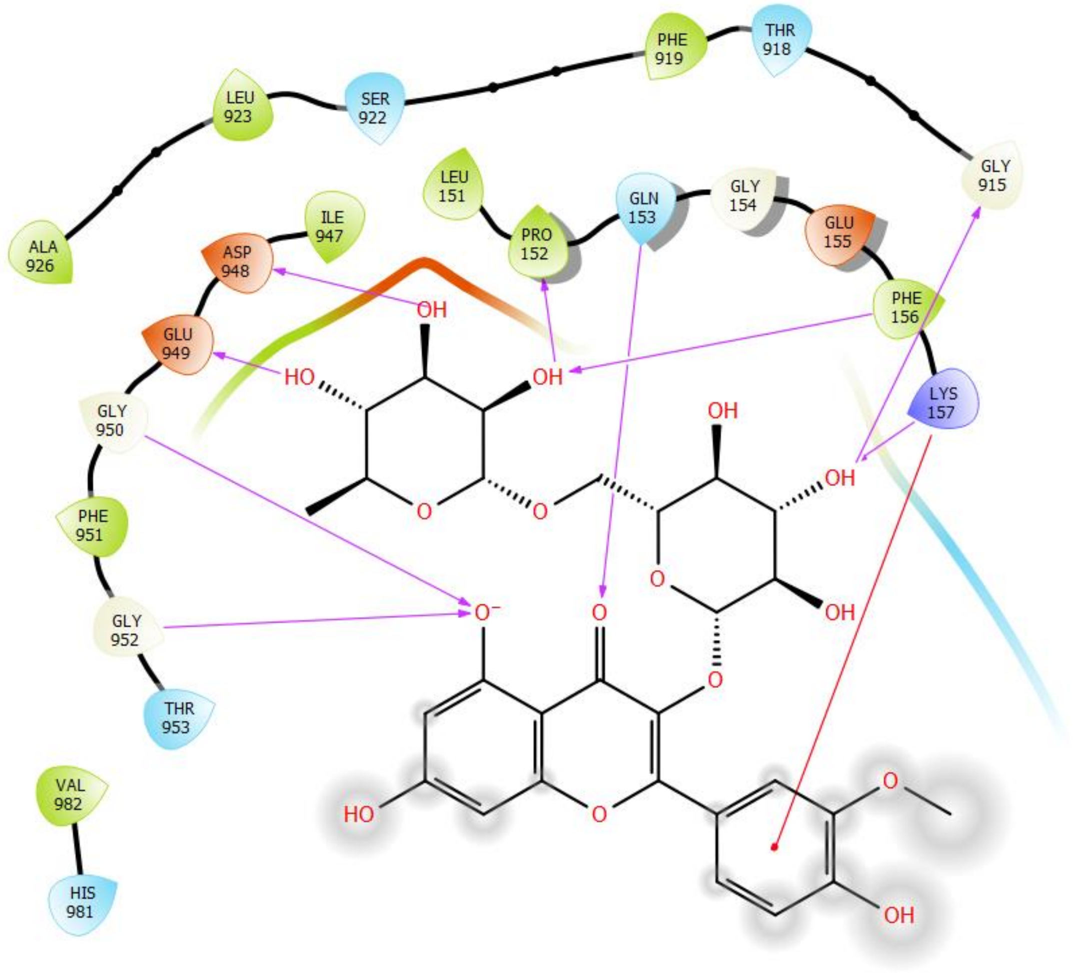

Figure S26. Protein-compound interaction diagram formed between isorhamnetin-3-*O*-rutinoside and nuclease SbcCD subunit C.

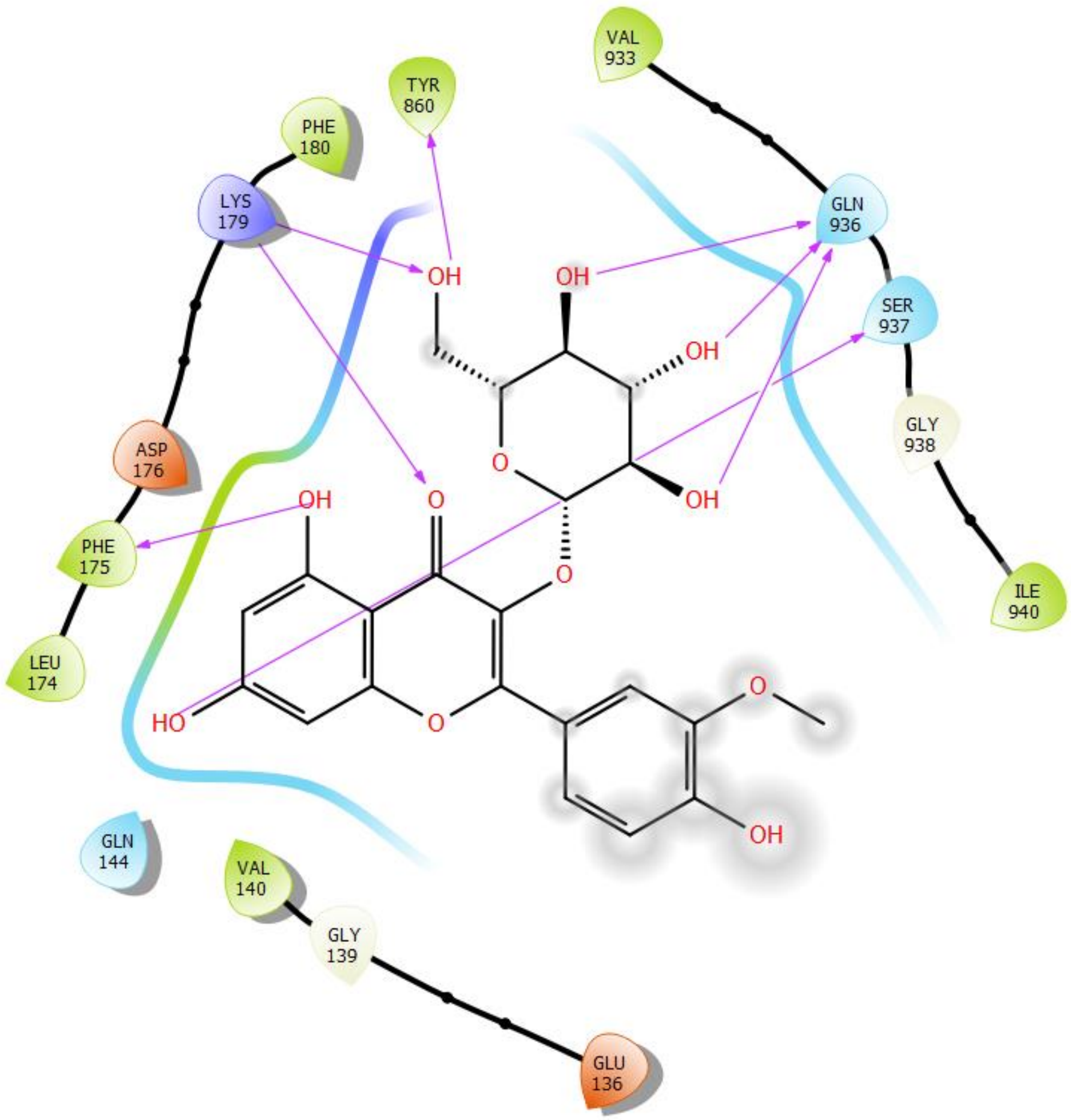

Figure S27. Protein-compound interaction diagram formed between isorhamnetin 3-*O*-*β*-D-glucoside and nuclease SbcCD subunit C.

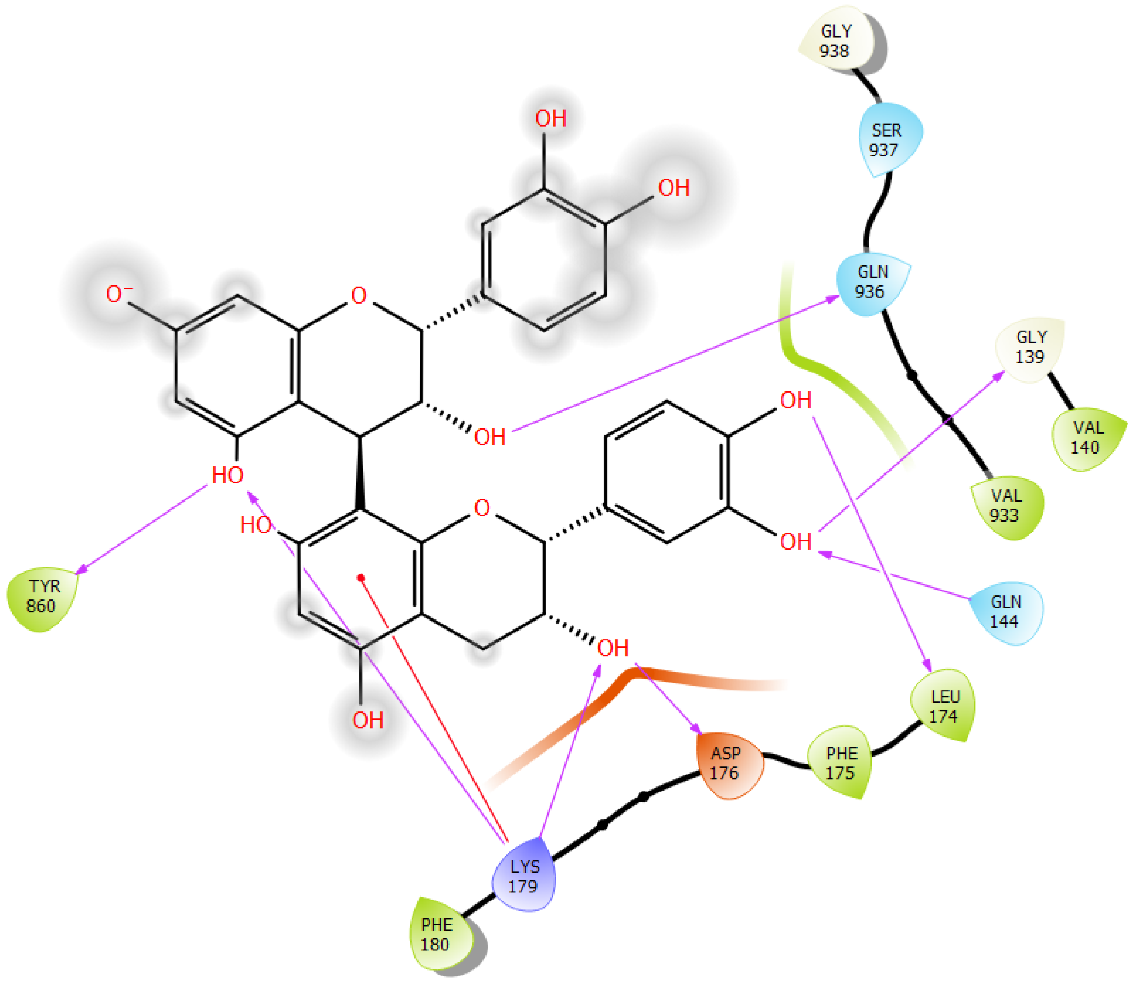

Figure S28. Protein-compound interaction diagram formed between procyanidin B2 and nuclease SbcCD subunit C.

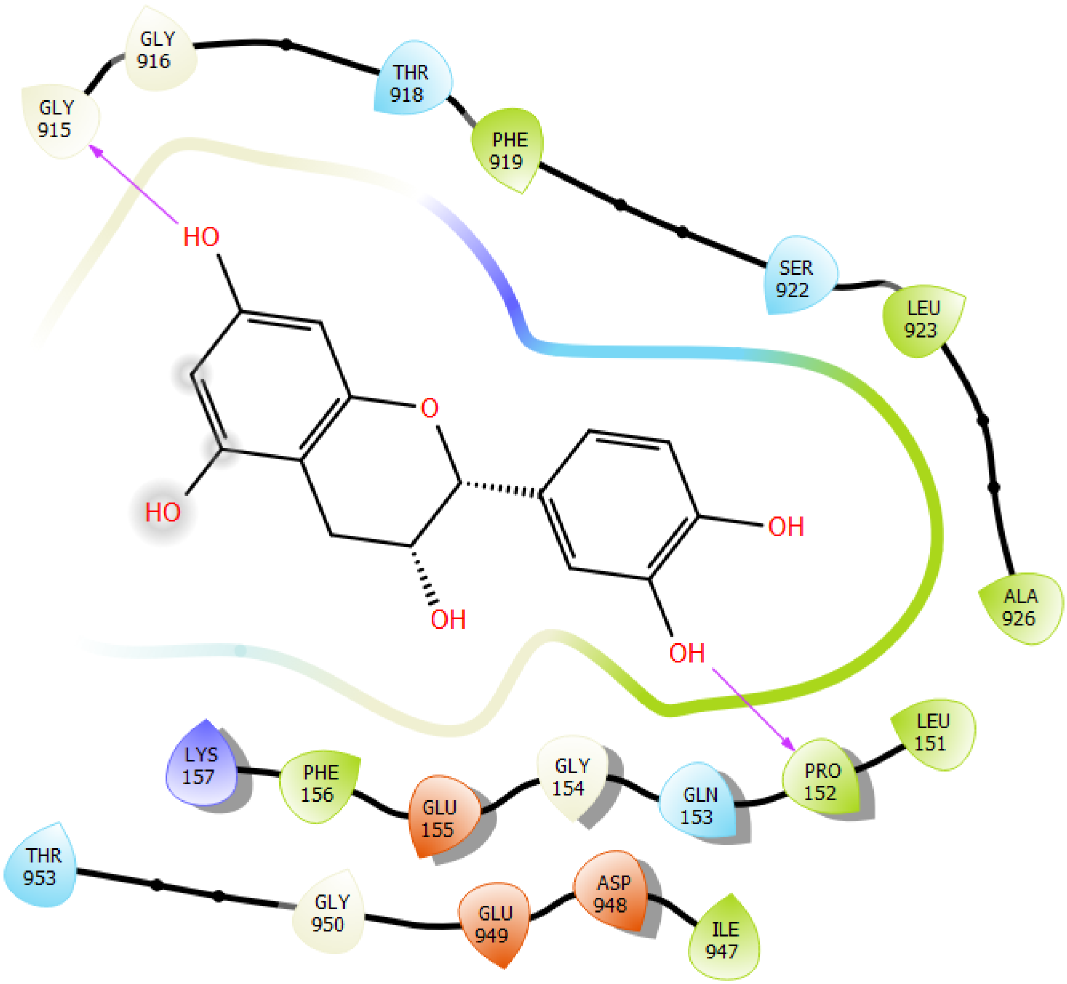

Figure S29. Protein-compound interaction diagram formed between epicatechin and nuclease SbcCD subunit C.

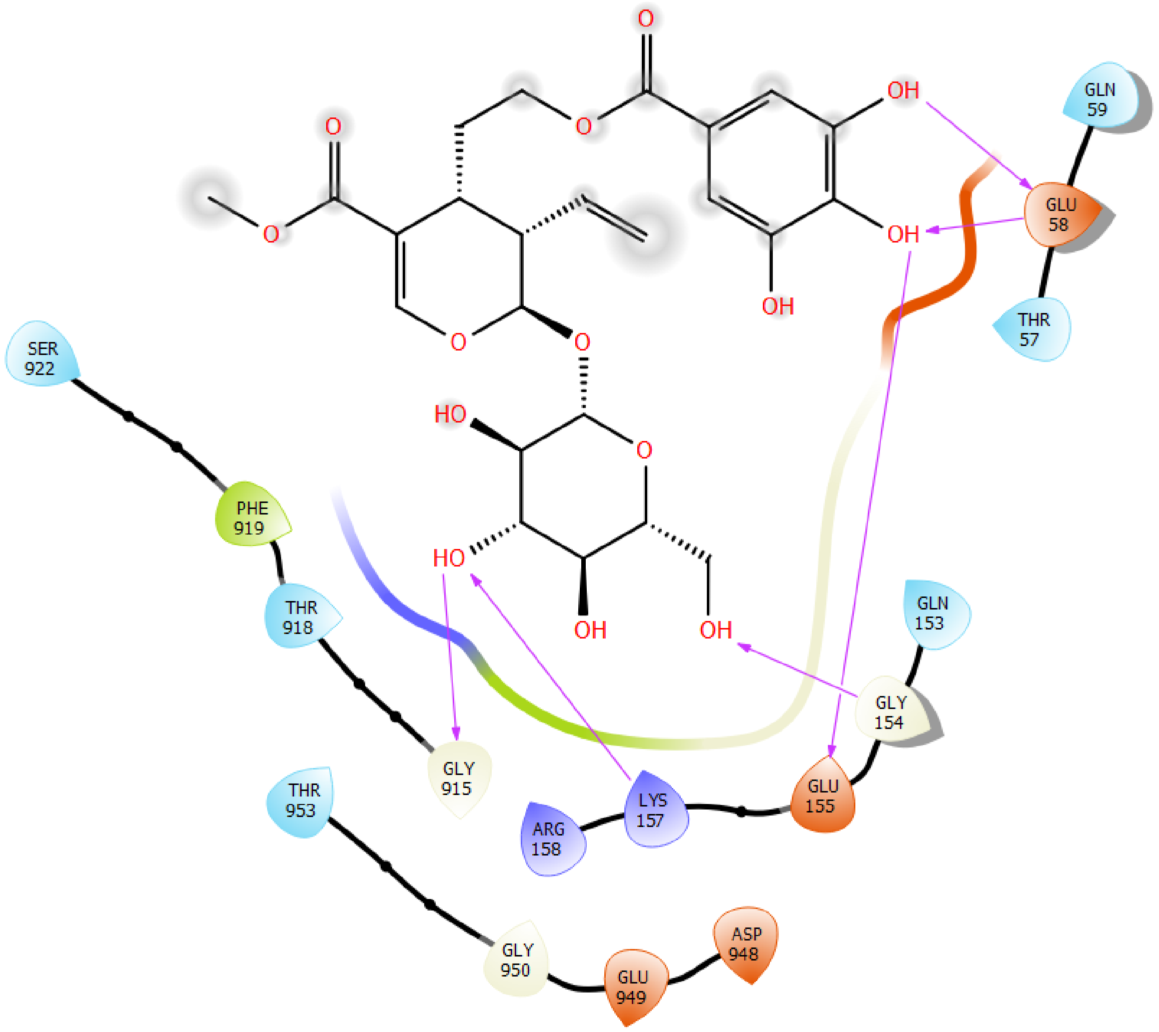

Figure S30. Protein-compound interaction diagram formed between cornuside and nuclease SbcCD subunit C.

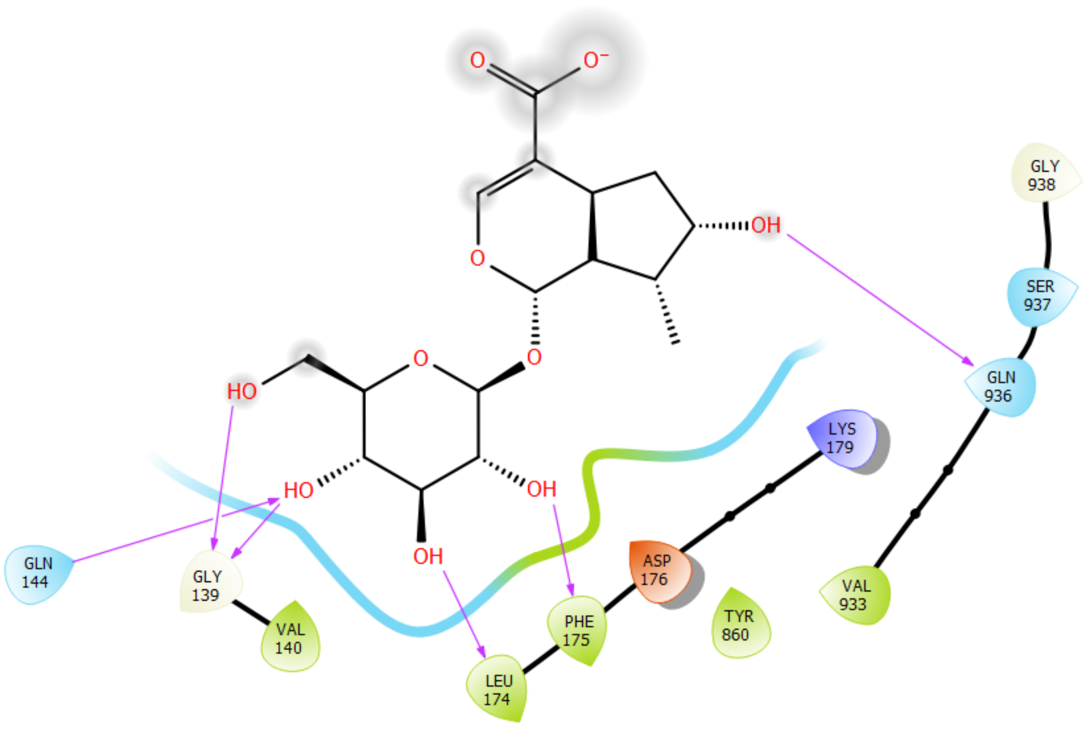

Figure S31. Protein-compound interaction diagram formed between loganic acid and nuclease SbcCD subunit C.

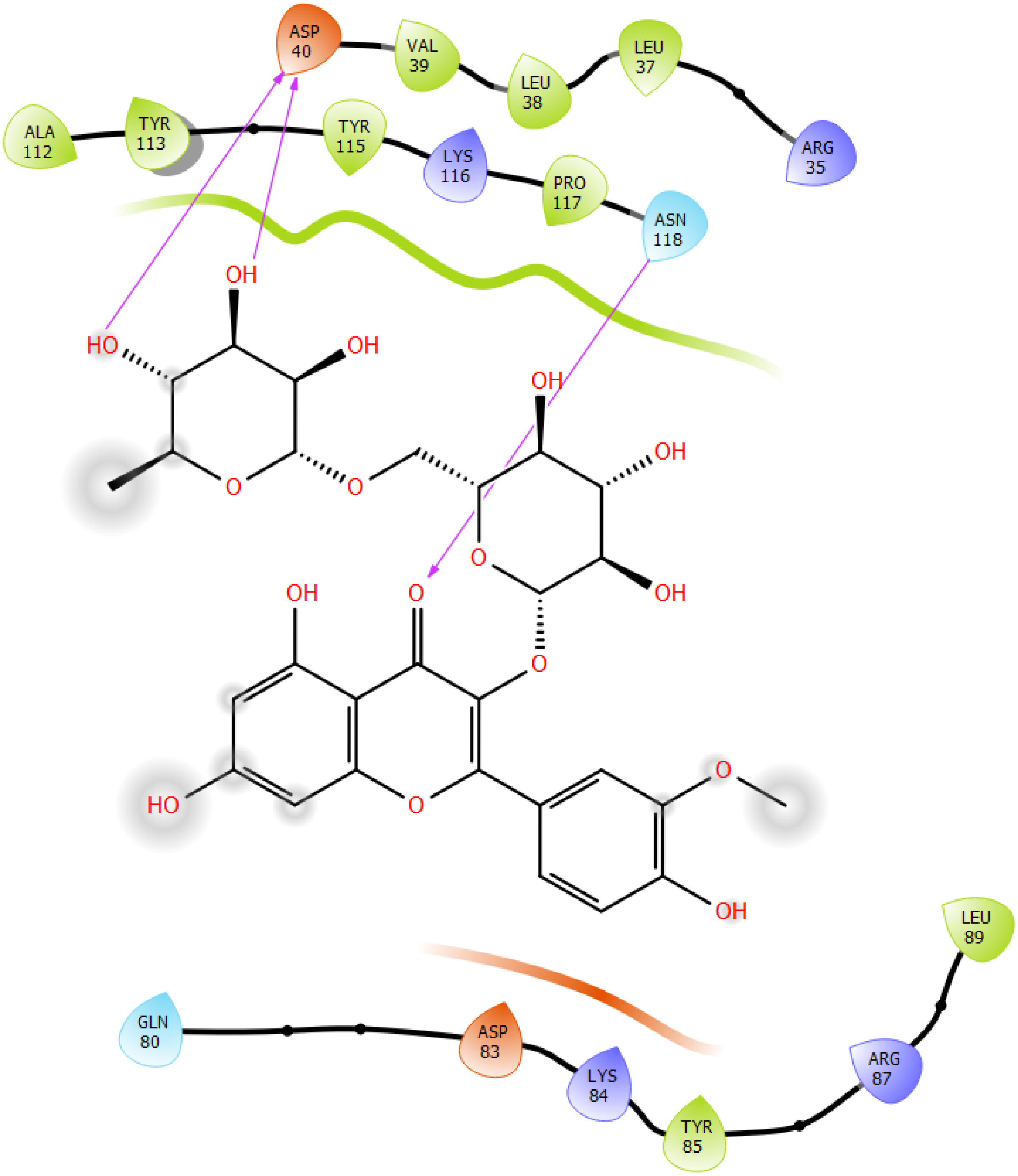

Figure S32. Protein-compound interaction diagram formed between isorhamnetin-3-*O*-rutinoside and thermonuclease.

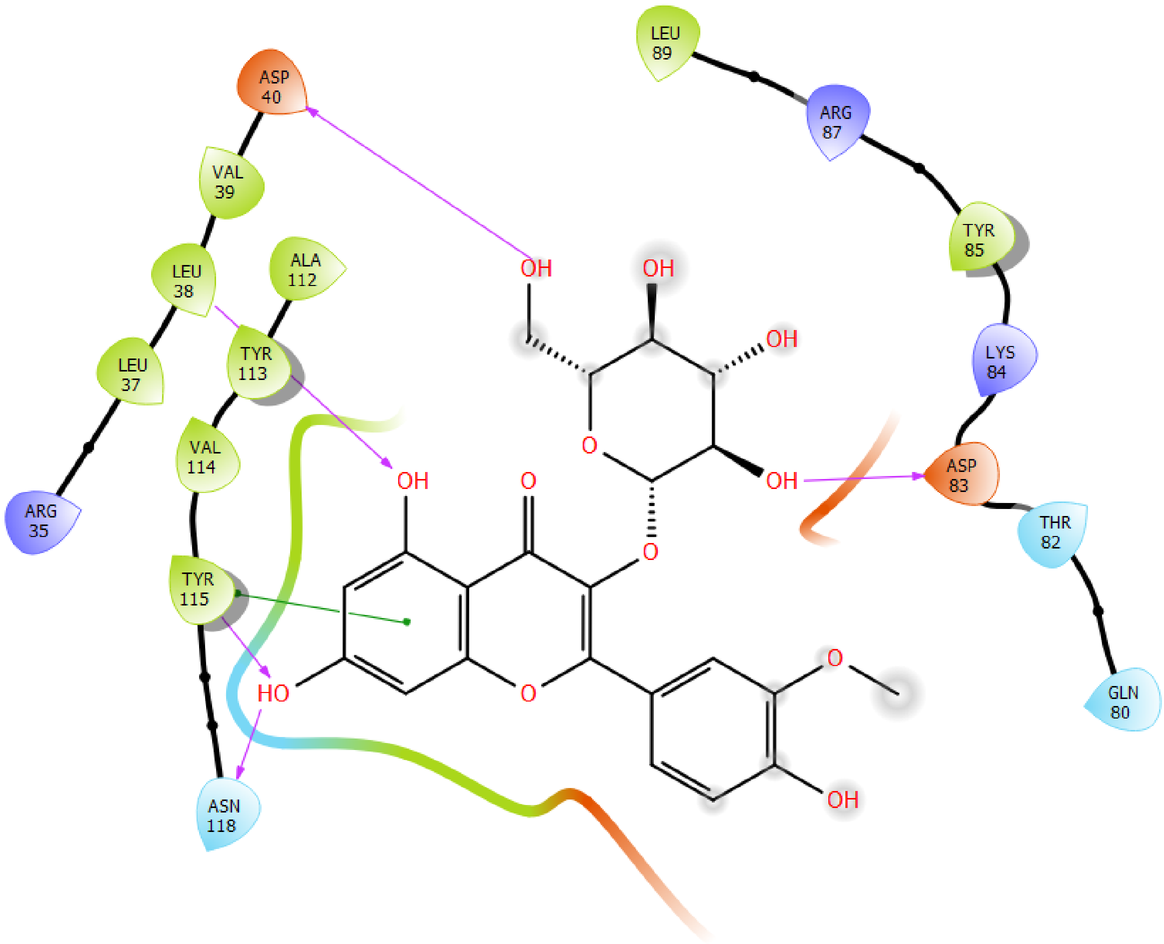

Figure S33. Protein-compound interaction diagram formed between isorhamnetin 3-*O*-*β*-D-glucoside and thermonuclease.

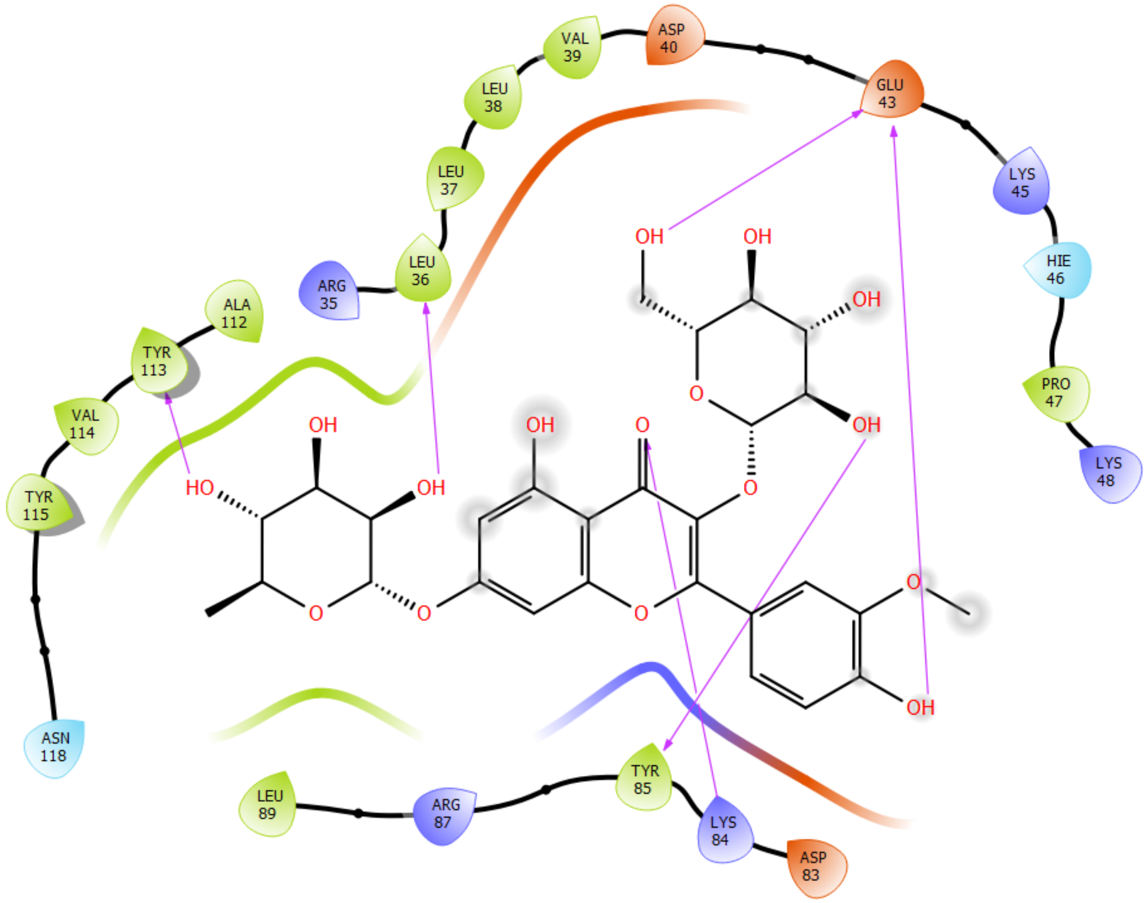

Figure S34. Protein-compound interaction diagram formed between isorhamnetin-3-*O*-*β*-D-glucosyl-7-*O*-*α*-L-rhamnoside and thermonuclease.

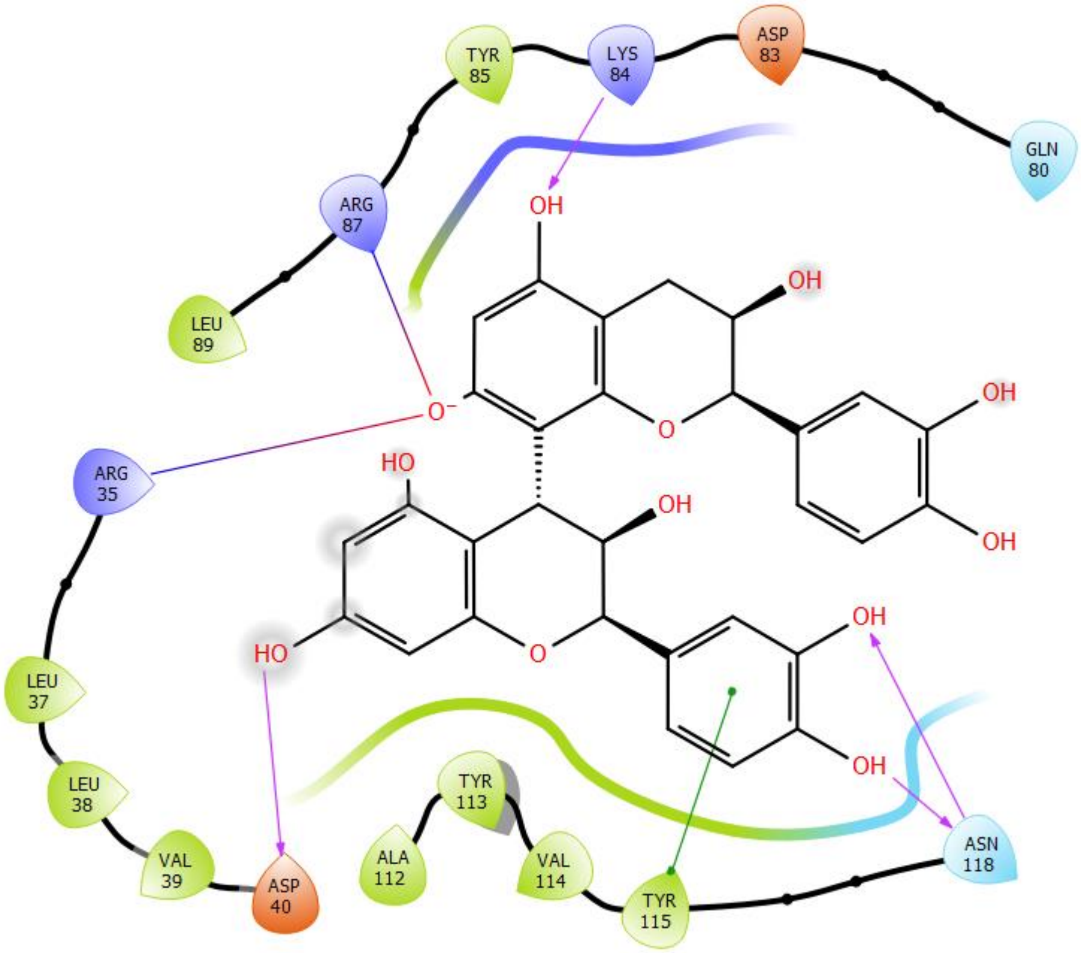

Figure S35. Protein-compound interaction diagram formed between procyanidin B2 and thermonuclease.

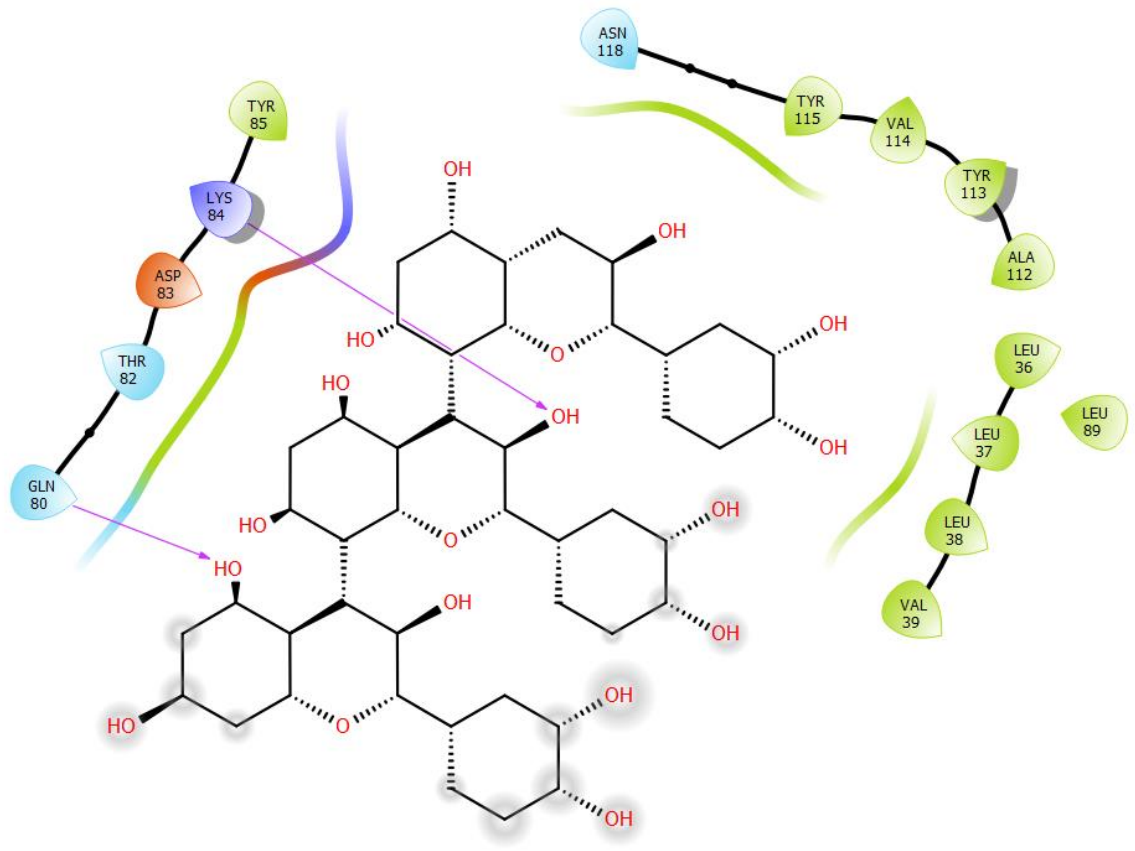

Figure S36. Protein-compound interaction diagram formed between procyanidin C1 and thermonuclease.

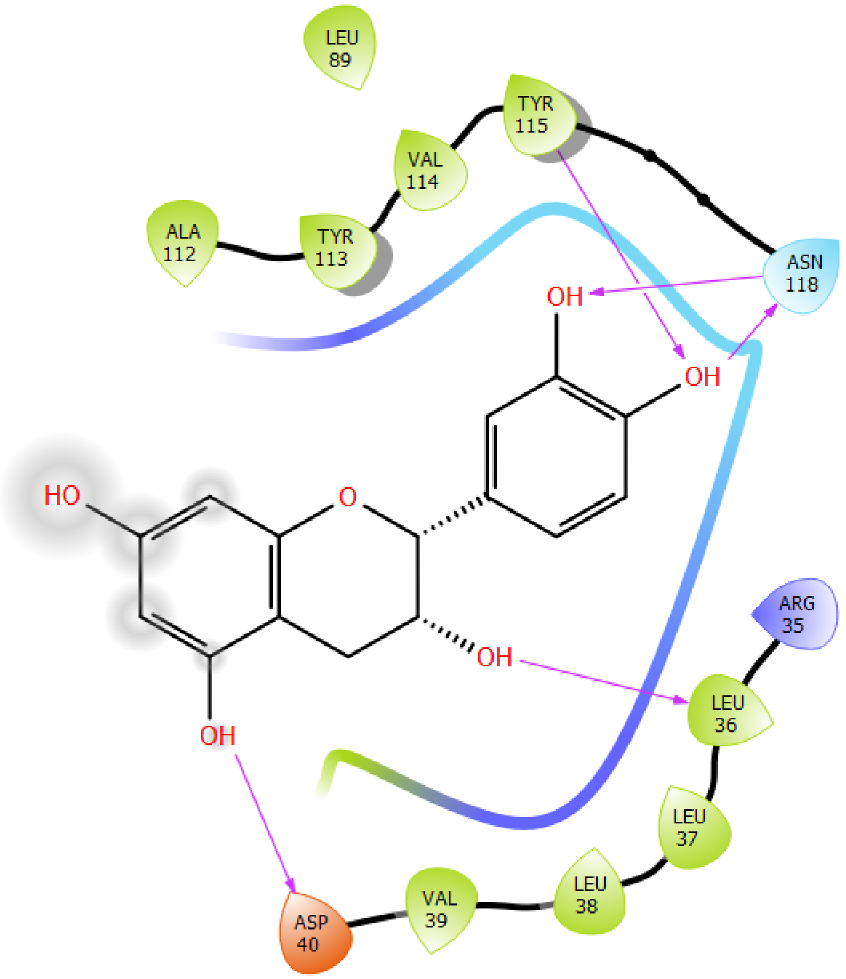

Figure S37. Protein-compound interaction diagram formed between epicatechin and thermonuclease.

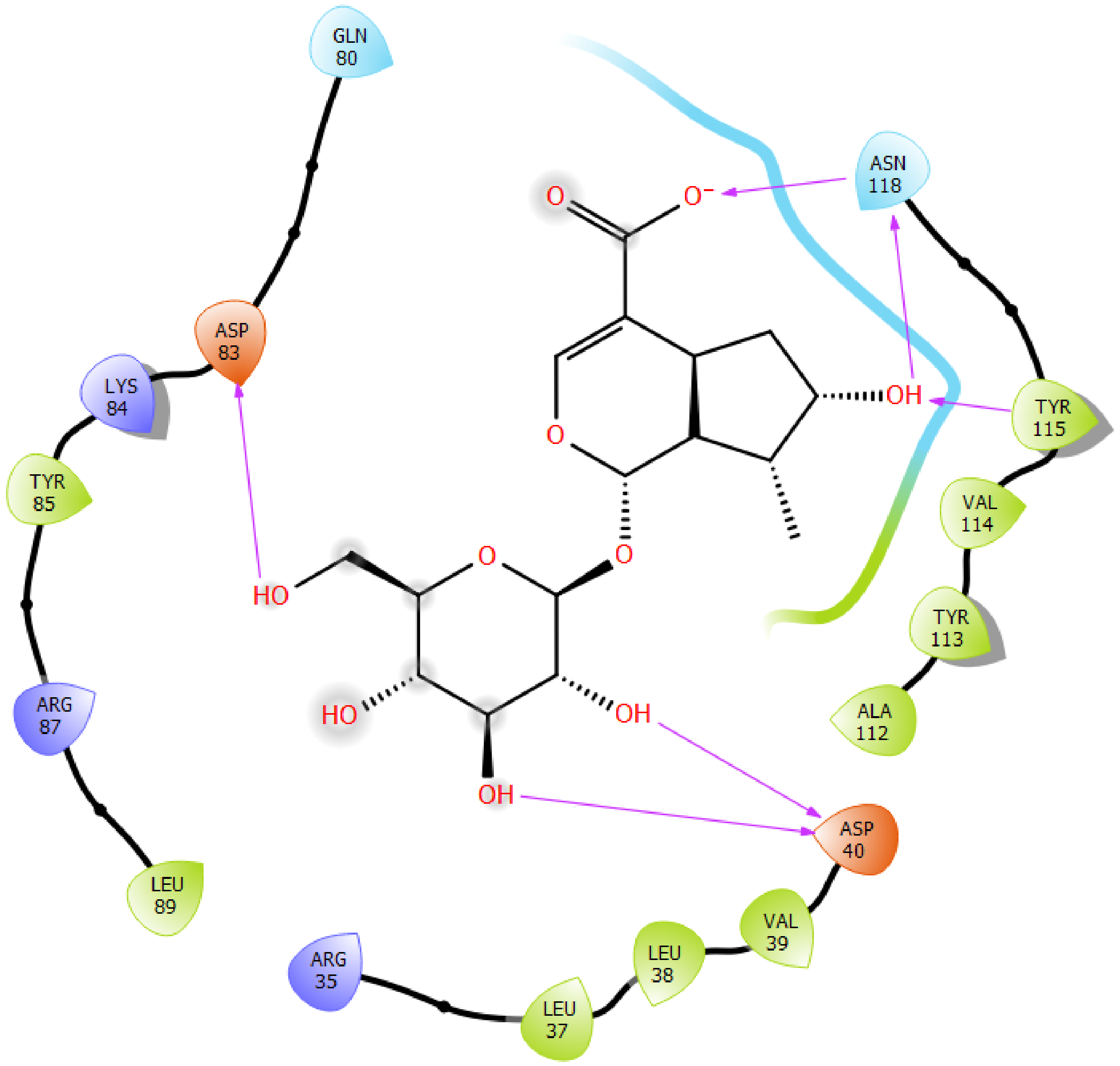

Figure S38. Protein-compound interaction diagram formed between loganic acid and thermonuclease.

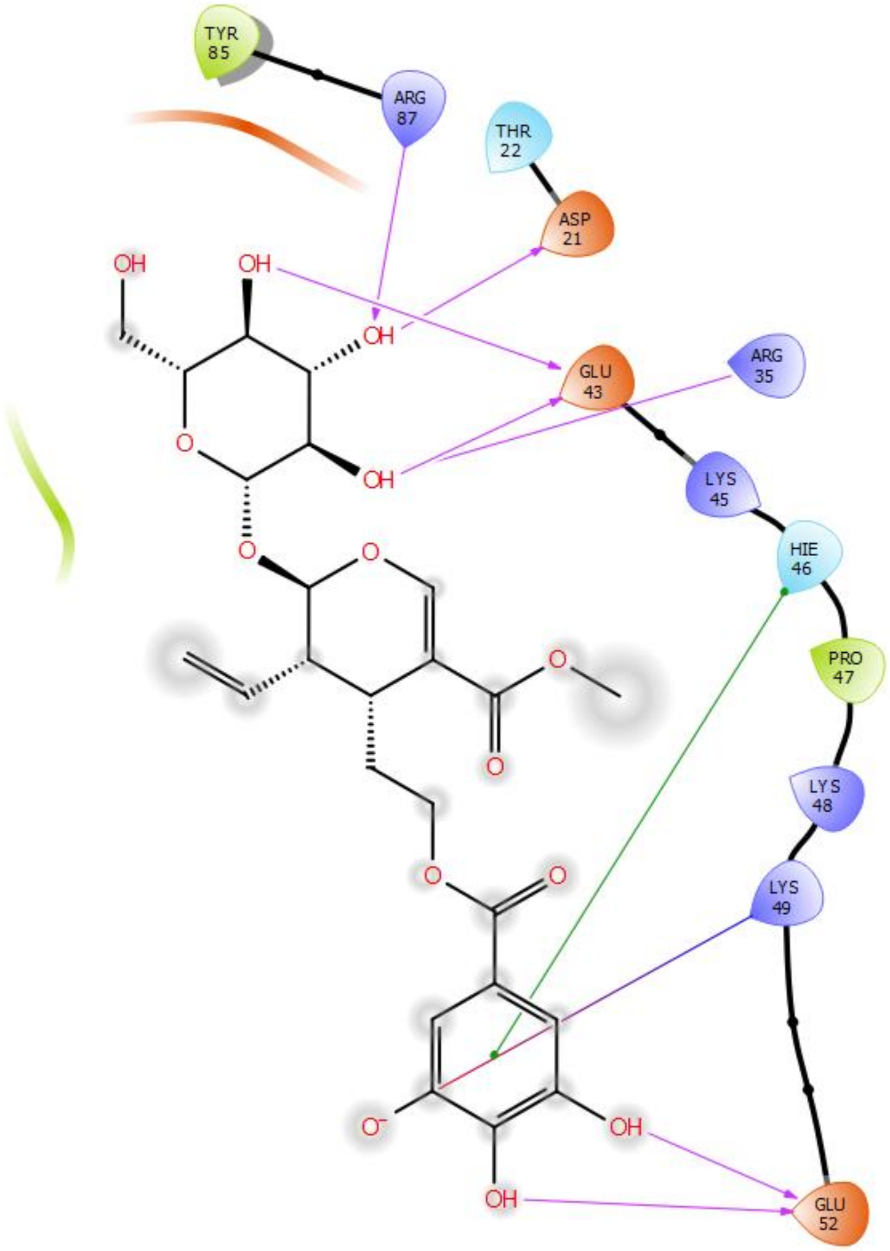

Figure S39. Protein-compound interaction diagram formed between cornuside and thermonuclease.

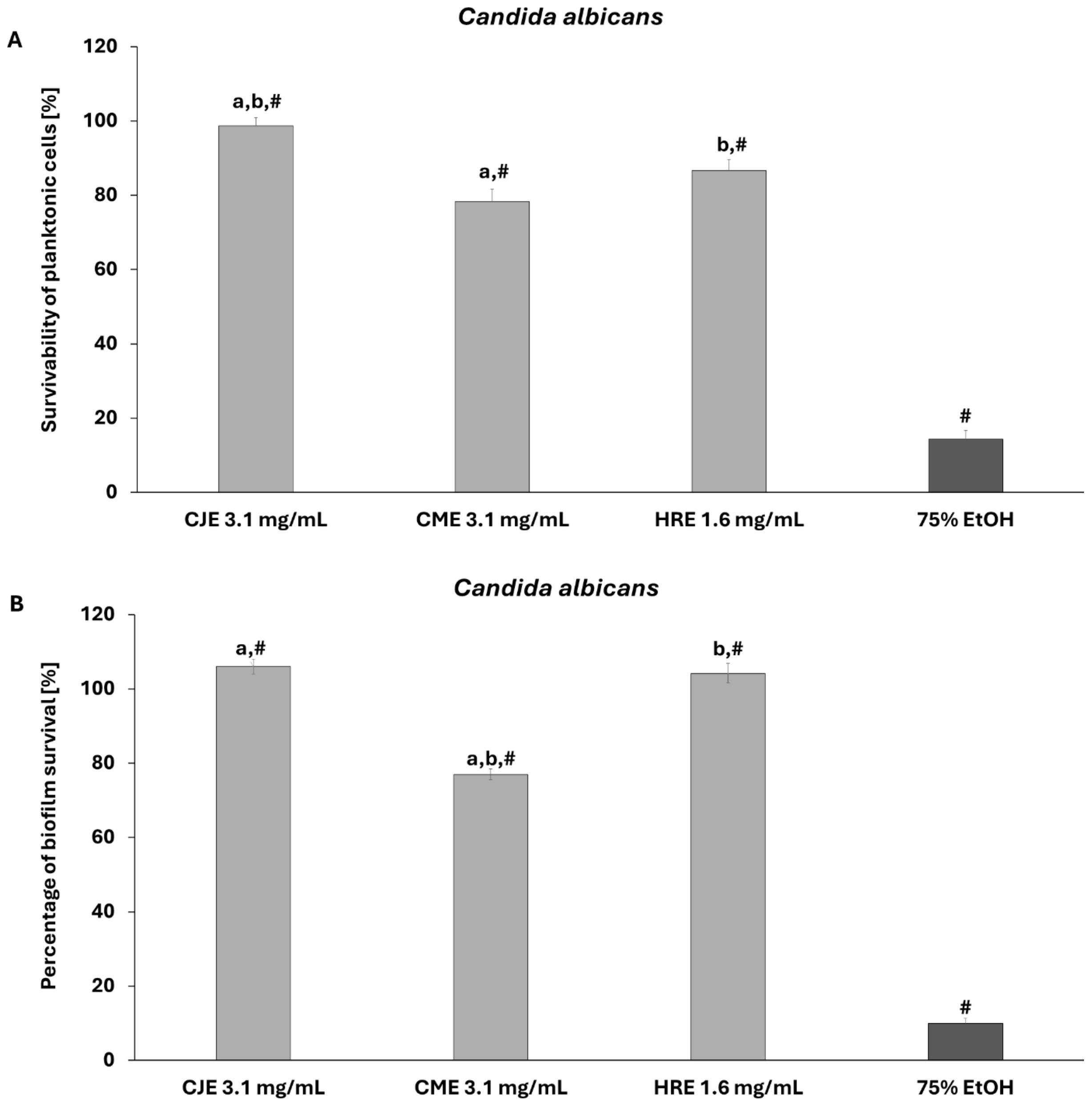

Figure S40. The effect of extracts on survival of planktonic cells (A) or biofilm (B) formed by *C. albicans*. The survivability of microbial cells exposed to sterile saline was considered 100%. CJE – aqueous extract from fruits of *Chaenomeles japonica*; CME – ethanolic (60%) extract from fruits of *Cornus mas*; HRE – aqueous extract from the fruit of *Hippophaë rhamnoides*. The pairs of letters mean the statistical significance ($P<0.05$) between extracts; #$P<0.05$ 75% EtOH vs. CJE, CME, and HRE.
